# Novel Amylin‐Based Therapies for Weight Management in Adults With Overweight or Obesity Without Diabetes: A Network Meta‐Analysis

**DOI:** 10.1002/edm2.70247

**Published:** 2026-05-22

**Authors:** A. B. M. Kamrul‐Hasan, Ibrahim Khalil, Kunal Mahajan, Deep Dutta, Mainak Banerjee, Joseph M. Pappachan

**Affiliations:** ^1^ Department of Endocrinology Mymensingh Medical College Mymensingh Bangladesh; ^2^ Department of Medicine Dhaka Medical College and Hospital Dhaka Bangladesh; ^3^ Department of Cardiology Himachal Heart Institute Mandi Himachal Pradesh India; ^4^ Department of Endocrinology CEDAR Superspeciality Healthcare New Delhi India; ^5^ Department of Endocrinology, Rabindranath Tagore International Institute of Cardiac Sciences RN Tagore Hospital Kolkata West Bengal India; ^6^ Department of Endocrinology Ramakrishna Mission Seva Pratishthan (RKMSP) & Vivekananda Institute of Medical Sciences (VIMS) Kolkata West Bengal India; ^7^ Department of Endocrinology and Metabolism Lancashire Teaching Hospitals NHS Trust Preston UK; ^8^ Faculty of Science Manchester Metropolitan University Manchester Greater Manchester UK; ^9^ Department of Endocrinology, Kasturba Medical College, Manipal Manipal Academy of Higher Education Manipal Karnataka India

**Keywords:** amycretin, amylin receptor agonists, cagrilintide, CagriSema, eloralintide, obesity

## Abstract

**Background:**

Long‐acting amylin‐based therapies (ABTs) are emerging anti‐obesity agents; we sought to compare their effects on weight and anthropometric outcomes in adults with overweight/obesity without diabetes, evaluate gastrointestinal (GI) safety, and rank agents and doses within a network meta‐analysis (NMA) framework.

**Methods:**

We conducted a frequentist random‐effects NMA of randomized controlled trials comparing novel ABTs with placebo or active comparators in R. Primary outcome was the percent change in body weight from baseline. Secondary outcomes included absolute weight changes, anthropometric measures, and overall and specific GI adverse events (AEs). Treatments (including dose strata) were compared with placebo within a single network and ranked using P scores.

**Results:**

Six trials (*N* = 4642; 12–68 weeks) were included. Compared with placebo, high dose (HiD) subcutaneous amycretin produced the largest reduction in percent body weight (mean difference −23.95%; P score 1.00), followed by HiD eloralintide (−18.01%; P score 0.89) and HiD CagriSema (−17.18%; P score 0.85), all exceeding semaglutide 2.4 mg (−11.45%) and liraglutide 3.0 mg (−6.4%). Almost similar patterns were observed for absolute weight, body mass index, waist circumference and categorical weight‐loss thresholds. GI AEs, nausea, vomiting and constipation were more common with HiD ABTs, especially oral amycretin and CagriSema, while diarrhoea mainly increased with semaglutide 2.4 mg. Only HiD CagriSema increased AE‐related discontinuation.

**Conclusions:**

Novel ABTs, such as HiD amycretin, CagriSema and eloralintide, may induce substantial short‐ to medium‐term weight loss but may also increase GI AEs; given sparse, low‐certainty data, these findings are preliminary and require confirmation in larger trials.

**Trial Registration:**

The meta‐analysis was registered in PROSPERO (CRD420261340457). The review protocol summary can be accessed at the PROSPERO website (https://www.crd.york.ac.uk/PROSPERO/view/CRD420261340457)

## Graphical abstract

This network meta‐analysis of 6 RCTs (4642 participants) found that novel amylin‐based therapies (particularly high‐dose subcutaneous amycretin and eloralintide) produced substantial weight loss in adults with overweight or obesity without diabetes, outperforming placebo.

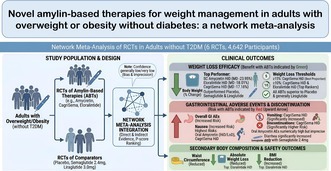

**Abbreviations**
ABT: amylin‑based therapy
AEs: adverse events
BMI: body mass index
CI: confidence interval
CINeMA: confidence in network meta‐analysis
GI: gastrointestinal
GLP‐1: glucagon‐like peptide‐1
GLP‑1RA: glucagon‑like peptide‑1 receptor agonist
HiD: high dose
LoD: low dose
MD: mean difference
NMA: network meta‐analysis
PICOS: the population, intervention, comparison, outcomes and study design
PRISMA: preferred reporting items for systematic reviews and meta‐analyses
RCT: randomized controlled trial
RoB: risk of bias
RR: risk ratio
SC: subcutaneous
SD: standard deviation
SGLT2: sodium‐glucose cotransporter‐2
T2D: type 2 diabetes

## 1 Introduction

Obesity is a growing global health concern that affects many aspects of people's lives, including physical health, mental well‐being and social interactions [1]. Current anti‐obesity drugs, including glucagon‐like peptide‐1 (GLP‐1) receptor agonists (GLP‐1RAs) and dual incretin agonists, can induce substantial weight loss but are limited by inconsistent responses, gastrointestinal side effects, access constraints, and the frequent need for long‐term, injectable regimens [2]. This therapeutic gap has renewed interest in amylin, a pancreatic β‐cell hormone that complements insulin by promoting satiety, slowing gastric emptying, suppressing postprandial glucagon and modulating energy expenditure via distinct amylin receptor subtypes in the area postrema, nucleus tractus solitarius and hypothalamus [3].

Amylin is a β‐cell–derived peptide hormone co‐secreted with insulin that acts in the area postrema, nucleus tractus solitarius and hypothalamus to promote satiety, slow gastric emptying, suppress postprandial glucagon and modulate energy expenditure through amylin receptor complexes composed of calcitonin receptors and receptor activity–modifying proteins [3]. First‐generation amylin analogs such as pramlintide demonstrated proof of concept for weight loss and prandial glucose control in diabetes but were limited by a short half‐life, the need for multiple daily injections, modest weight‐loss efficacy, and tolerability concerns, which restricted their use for long‐term obesity treatment [4, 5]. Advances in receptor structural biology, peptide engineering, and half‐life extension technologies (including albumin binding and lipidation) have enabled a second generation of long‐acting, non‐aggregating amylin‐based therapies (ABTs) that more effectively harness the anorectic and energy‐balance effects of amylin signalling. These ABTs include selective amylin receptor agonists (e.g., eloralintide), dual amylin–calcitonin receptor agonists (e.g., cagrilintide, petrelintide) and multi‐agonist approaches that either combine amylin and GLP‐1 receptor agonism in a single molecule (e.g., amycretin) or use fixed‐dose combinations of different agonists such as CagriSema (semaglutide plus cagrilintide), to integrate complementary mechanisms in appetite regulation, gastric motility and metabolic control [6, 7]. In Phase 1–3 trials in individuals with overweight or obesity, largely without diabetes, these agents have produced placebo‐subtracted weight losses of approximately 7%–20%, with emerging evidence of benefits in glycemic control, blood pressure and liver fat, alongside a typical pattern of dose‐related gastrointestinal (GI) adverse events (AEs) similar to those observed with GLP‐1 RAs [8, 9, 10, 11, 12, 13].

However, existing trials vary in populations, doses, classes of molecules, routes of administration, duration, and endpoints, and comprehensive head‐to‐head comparisons across the amylin‐based pipeline are lacking. As a result, the relative efficacy and tolerability of individual amylin receptor agonists and amylin/GLP‐1 multi‐agonists in adults with overweight or obesity without diabetes remain unclear, and the optimal combinations of dose, route, and duration are unknown. Accordingly, this network meta‐analysis (NMA) has three main objectives: first, to compare the effects of novel ABTs versus placebo and active comparators on weight loss and key anthropometric outcomes in adults with overweight or obesity without diabetes; second, to assess and contrast safety and tolerability, focusing on GI AEs and treatment discontinuations; and third, to rank individual agents and regimens based on their likelihood of being the most effective and tolerated options. By integrating direct and indirect evidence from randomized controlled trials (RCTs), this NMA aims to provide a quantitative framework to inform clinical use, guideline development, and the design of future obesity trials for ABTs.

## 2 Methods

This NMA was prospectively registered in PROSPERO (CRD420261340457), with the protocol summary available online. It was conducted following the Cochrane Handbook for Systematic Reviews of Interventions, and its reporting adhered to the PRISMA extension statement for reporting systematic reviews incorporating network meta‐analyses of health care interventions [14, 15].

### 2.1 Search Strategy

Several databases and registries, including PubMed, Scopus, Web of Science, the Cochrane Central Register of Controlled Trials (CENTRAL), and ClinicalTrials.gov, were systematically searched. The search spanned these sources from their inception to 10 March 2026. Using the Boolean operators ‘AND’ and ‘OR’ the following terms were searched: ‘obesity’, ‘overweight’, ‘weight management’, ‘amylin receptor agonist’, ‘amylin agonist’, ‘amylin analog’, ‘amylin‐based therapy’, ‘long‐acting amylin analog’, ‘long‐acting amylin agonist’, ‘dual amylin‐calcitonin receptor agonist’, ‘dual amylin‐GLP‐1 receptor agonist’, ‘pramlintide’, ‘cagrilintide’, ‘AM833’, ‘cagrilintide–semaglutide’, ‘CagriSema’, ‘amycretin’, ‘eloralintide’, ‘LY3841136’, ‘petrelintide’, ‘ZP8396’, ‘AZD6234’, ‘MET‐233’, ‘NN1213’, ‘Amylin‐355’, ‘NN9638’, ‘KBP‐042’, ‘KBP‐066’, ‘KBP‐066A’, ‘KBP‐088’, ‘KBP‐088A’, ‘KBP‐089’, ‘ASC36’ and ‘GUB014295’. The search targeted MeSH terms, titles, abstracts and keywords in the documents, with no language restrictions, to identify published studies. The full search strategies are provided in Table S1. Additionally, the process included reviewing the references cited in the articles collected for this research and in relevant journals.

### 2.2 Study Selection

The Population, Intervention, Comparison, Outcomes and Study (PICOS) framework guided the development of eligibility criteria for the clinical trials included in this NMA. The patient population (P) included individuals with overweight or obesity who did not have diabetes. The intervention (I) included any long‐acting ABTs, such as selective amylin receptor agonists, dual amylin‐calcitonin receptor agonists, and multi‐agonists that combine amylin and GLP‐1 activity. The control (C) group received either a placebo or an active weight‐loss intervention. The outcomes (O) included changes in body weight (percent and absolute) from baseline and adverse events associated with the interventions. We included only RCTs with a minimum duration of 12 weeks and participants aged 18 or older. We excluded RCTs conducted exclusively in people with type 2 diabetes because glycemic status, concurrent glucose‐lowering medications, and regulatory dose regimens can substantially modify weight‐loss responses. We aimed to reduce clinical heterogeneity by focusing on individuals with overweight or obesity who do not have diabetes. Excluded from the analysis were nonrandomized trials, retrospective studies, pooled clinical trial analyses, conference proceedings, letters to editors, case reports, and articles lacking outcome data of interest. Additionally, studies involving animals or normal‐weight humans, as well as RCTs shorter than 12 weeks, were excluded.

### 2.3 Outcomes Analyzed

The primary outcome of interest was the percent change in body weight from baseline to the end of the study. The secondary outcomes included the absolute change in body weight, changes in body mass index (BMI) and waist circumference, and the proportions of study subjects who lost ≥ 5%, ≥ 10% or ≥ 15% of their baseline body weight. Safety outcomes included overall GI AEs, nausea, vomiting, diarrhoea, constipation and treatment discontinuations.

### 2.4 Data Extraction

Three authors independently extracted data using standard forms. When multiple publications originated from the same study group, their results were combined, and relevant data from each report were included in the analysis. Patient characteristics, including demographic details and comorbidities, from both included and excluded studies were recorded. For the review, we extracted data from all eligible studies on several key factors: first author, publication year, clinical trial unique identifier, study design and settings, major inclusion criteria, study arms, sample size, percentage of female participants, mean age, baseline body weight, BMI, waist circumference and study duration. Data on both primary and secondary outcomes were also collected, as previously mentioned. For continuous outcomes, we extracted mean differences (MDs) and standard deviations (SDs) between baseline and end‐of‐study values. The data were converted to MD (±SD) when reported in other formats (e.g., standard error, median, confidence intervals [CIs]) using the web‐based Meta‐Analysis Accelerator [16]. For categorical variables, we recorded the number of participants who achieved the specific outcome and the total number of participants. The Supporting Information of the relevant studies were carefully reviewed. All pertinent information was obtained through written email communication with the corresponding author of the relevant article and was thoughtfully incorporated into the meta‐analysis.

### 2.5 Risk of Bias Assessment

Three authors independently assessed risk of bias (RoB) using version 2 of the Cochrane risk‐of‐bias tool for randomized trials (RoB 2) [17]. The domains covered by RoB 2 encompass all types of bias currently recognized as affecting the results of RCTs, including bias arising from randomization, deviations from intended interventions, missing outcome data, outcome measurement, and the selection of reported results. The RoB judgement categorized each domain into one of three levels: low RoB, some concerns or high RoB. The least favourable assessment across the bias domains was used to determine the overall RoB for the result [17]. Disagreements were settled through consensus. Publication bias was assessed visually using funnel plots, followed by Egger's test for a quantitative assessment [18].

### 2.6 Data Synthesis and Statistical Analysis

Transitivity was evaluated descriptively by comparing key baseline characteristics (including age, sex and BMI) across trials and treatment comparisons, as recommended by PRISMA‐NMA guidance. A frequentist random‐effects NMA was performed in RStudio (Version 2026.01.1+403), primarily using the netmeta package [19]. Treatment effects for both primary and secondary outcomes were estimated as MDs for continuous outcomes and risk ratios [RRs] for binary outcomes, along with 95% CIs, for each comparison against the reference treatment, that is, placebo. To explore dose–response relationships and clinical dosing variation, the ABTs were grouped by dose. Network plots were produced to visualize the structure and connectivity of the treatment comparisons, illustrating direct and indirect evidence pathways. From the fitted network model, relative effects for all possible pairwise treatment comparisons were obtained and summarized in league tables, whereas forest plots present the estimated effects of each active treatment versus placebo. Inconsistency between direct and indirect evidence was evaluated using the netsplit function with the back‐calculation method. Treatments were ranked from best to worst according to their relative effects along the leading diagonal of the league tables, and P‐scores were calculated to quantify the probability that each treatment was among the most effective options (higher values indicating greater effectiveness). Statistical significance was defined as *p* < 0.05. Between‐study heterogeneity was assessed using Cochran's *Q* test and the *I* ^2^ statistic, with *τ* ^2^ estimated by restricted maximum likelihood and *I* ^2^ derived from *Q*; *I* ^2^ values of approximately 25%, 50% and 75% were interpreted as low, moderate and high heterogeneity, respectively [20, 21].

### 2.7 Assessment of the Certainty of Evidence

We evaluated the certainty of evidence for the primary outcome (percent change in body weight) using the CINeMA framework. CINeMA assesses six domains: within‐study bias, across‐study bias, indirectness, imprecision, heterogeneity and incoherence. For each pairwise comparison, judgements across domains were combined to produce an overall confidence rating (high, moderate, low or very low) for mixed and indirect evidence [22].

## 3 Results

### 3.1 Search Results

**FIGURE 1:**
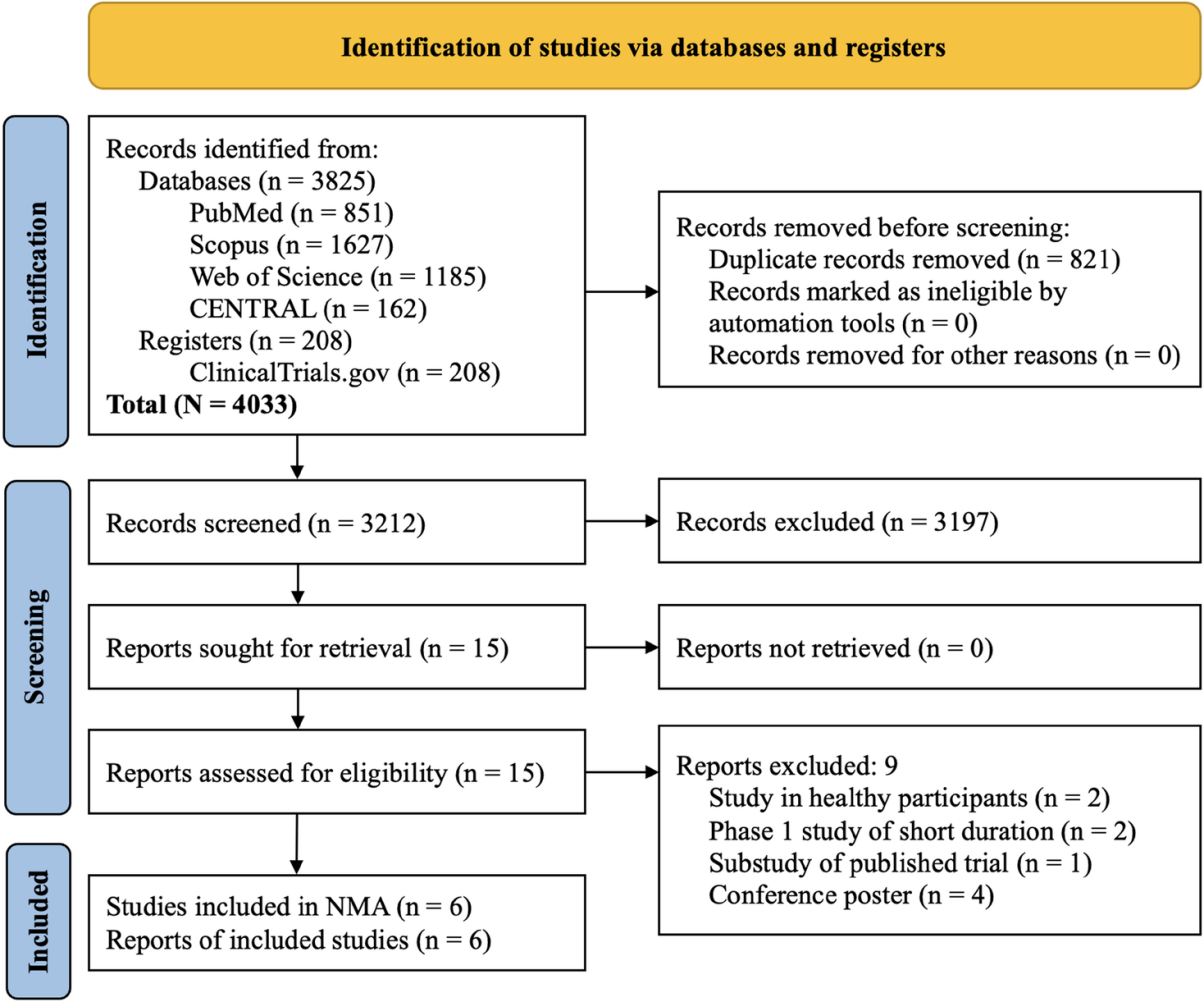
Flowchart on study retrieval and inclusion in the meta‐analysis.

The study selection process is summarized in Figure 1. The initial search identified 4033 records. After removing duplicates and screening titles, abstracts and full texts, 15 articles were assessed in detail, and 6 RCTs (reported in 6 publications) involving 4642 participants and follow‐up durations of 12–68 weeks met the inclusion criteria for the NMA [8, 9, 10, 11, 12, 13].

### 3.2 Characteristics of Included Studies

Key characteristics of the included RCTs are shown in Table 1. Enebo 2021 and Gasiorek 2025 were phase 1 trials [10, 13]. Dahl 2025 was a phase 1b/2a trial [12]. Lau 2021 and Billings 2025 were phase 2 trials [8, 11] and Garvey 2025 was a phase 3a trial [9]. In the intervention group, Lau et al. [8] used once‐weekly subcutaneous (SC) injections of cagrilintide at doses of 0.3 mg to 4.5 mg; once‐daily SC liraglutide 3.0 mg and placebo were in the control arms. Garvey et al. [9] compared once‐weekly SC CagriSema (a combination of semaglutide 2.4 mg and cagrilintide 2.4 mg) with each molecule separately and with placebo. Enebo et al. [10] tested different doses of CagriSema (0.16/2.4–4.5/2.4 mg), with the control group receiving a placebo and semaglutide 2.4 mg. Billings et al. [11] used various doses of once‐weekly SC eloralintide (1–9 mg) as an intervention compared to a placebo control. Dahl et al. [12] had four parts: part A, a 23‐day study aimed at assessing treatment‐emergent AEs and the pharmacokinetics of once‐weekly SC amycretin, was not included in our analysis. Parts B, C, D and E, with different doses of SC amycretin (1.5–60 mg) as interventions and placebo as controls, were considered for the NMA [12]. Gasiorek et al. [13] administered oral amycretin tablets at various doses (50 mg, two 50 mg tablets and two 25 mg tablets) to the intervention groups, with a placebo control group. We categorized the doses of the intervention drugs as follows: cagrilintide 0.3–1.2 mg as low dose (LoD), and 2.4–4.5 mg as high dose (HiD); CagriSema 0.16–1.2/2.4 mg as LoD, and 2.4/2.4 and 4.5/2.4 mg as HiD; eloralintide 1–3 mg as LoD, and ≥ 6 mg (6 mg, 9 mg, and titration arms of 6–9 and 3–9 mg) as HiD; SC amycretin 1.25 and 5 mg as LoD, and 20 and 60 mg as HiD and oral amycretin 50 mg and 2 × 25 mg as LoD, and 2 × 50 mg as HiD. For each trial, we used the longest available follow‐up time point (range: 12–68 weeks) for weight outcomes because heterogeneous, often single‐time‐point designs prevented constructing a connected network at uniform time points (e.g., 12 or 24 weeks). The baseline characteristics of the included RCTs were consistent across all trial arms. Tables S2 and S3 provide the results of individual studies, including summary data for each intervention group. Although baseline BMI varied somewhat across agents (e.g., higher in eloralintide trials and lower in amycretin trials), all studies enrolled adults with overweight or obesity without diabetes, and we judged the populations and settings sufficiently similar to justify inclusion in a single treatment network.

**TABLE 1:** Characteristics of the included randomized controlled trials and trial participants.

Authors, publication year [ref.]	Trial reg. no.; Phase; study place	Study subjects	Study arms	*N*	Female (%)	Age (years)	Weight (kg)	BMI (kg/m^2^)	WC (cm)	Duration of follow up (weeks)
Lau et al. 2021 [8]	NCT03856047; Phase 2; 10 countries	Adults; BMI ≥ 30 or ≥ 27 kg/m^2^ with hypertension or dyslipidaemia	Cagrilintide 0.3 mg SC QW	101	55	53.5 (10.3)	109.8 (25.1)	38.4 (7.5)	116.2 (16.0)	26
			Cagrilintide 0.6 mg SC QW	100	62	53.2 (11.0)	106.2 (23.8)	37.2 (6.9)	114.5 (15.1)	
			Cagrilintide 1.2 mg SC QW	102	62	52.1 (8.7)	104 (21.5)	37.1 (6.2)	113.5 (15.0)	
			Cagrilintide 2.4 mg SC QW	102	74	52.7 (9.8)	106.8 (24.1)	37.9 (7.6)	113.7 (14.6)	
			Cagrilintide 4.5 mg SC QW	101	55	51.5 (12.7)	111.0 (28.6)	38.4 (7.7)	118.9 (17.6)	
			Liraglutide 3.0 mg SC QD	99	66	51.5 (9.3)	107.8 (24.1)	38.4 (7.4)	116.3 (15.4)	
			Placebo	101	58	51.4 (11.0)	106.2 (21.6)	37.4 (5.7)	114.7 (13.7)	
Garvey et al. 2025 [9]	NCT05567796; Phase 3a; 22 countries	Adults; BMI ≥ 30 or ≥ 27 kg/m^2^ with at least one obesity‐related complication	CagriSema 2.4/2.4 mg SC QW	2108	68.4	47.1 (11.9)	107.2 (23.2)	38.0 (6.8)	115.0 (15.7)	68
			Semaglutide 2.4 mg SC QW	302	65.9	47.4 (11.5)	105.9 (20.7)	37.4 (5.9)	114.2 (15.3)	
			Cagrilintide 2.4 mg SC QW	302	68.9	46.6 (11.2)	106.5 (22.9)	38.0 (6.4)	114.1 (14.9)	
			Placebo	705	65.4	46.7 (11.9)	106.6 (23.7)	37.8 (7.1)	114.1 (15.3)	
Enebo et al. 2021 [10]	NCT03600480; Phase 1b; USA	Age 18–55 years; BMI 27.0–39.9 kg/m^2^ and otherwise healthy	CagriSema 0.16/2.4 mg SC QW	12	33	43.0 (9.2)	93.0 (10.6)	31.0 (3.2)	NR	20
			CagriSema 0.3/2.4 mg SC QW	12	42	38.4 (10.4)	92.9 (11.7)	30.8 (2.3)	NR	
			CagriSema 0.6/2.4 mg SC QW	12	50	40.0 (8.3)	95.3 (11.7)	33.3 (3.7)	NR	
			CagriSema 1.2/2.4 mg SC QW	12	50	41.3 (11.1)	95.1 (14.4)	32.6 (4.4)	NR	
			CagriSema 2.4/2.4 mg SC QW	12	58	43.0 (8.1)	92.1 (11.9)	32.2 (2.5)	NR	
			CagriSema 4.5/2.4 mg SC QW	11	27	37.0 (9.7)	98.0 (17.3)	33.0 (4.2)	NR	
			Placebo + semaglutide 2.4 mg SC QW	24	33	41.0 (8.8)	99.6 (15.6)	32.2 (3.0)	NR	
Billings et al. 2025 [11]	NCT06230523; Phase 2; USA	Age 18–75 years; BMI ≥ 30 kg/m^2^ or ≥ 27 kg/m^2^ with at least one weight‐related comorbidity	Placebo	53	75	48.8 (12.3)	109.0 (22.0)	38.7 (5.7)	115.1 (14.5)	48
			Eloralintide 1 mg SC QW	28	75	50.4 (13.9)	113.0 (35.0)	40.6 (10.7)	118.9 (21.9)	
			Eloralintide 3 mg SC QW	24	79	48.6 (12.2)	107.8 (19.0)	37.9 (5.1)	113.4 (10.8)	
			Eloralintide 6 mg SC QW	28	75	46.1 (14.6)	106.9 (18.2)	37.9 (5.9)	116.1 (12.2)	
			Eloralintide 9 mg SC QW	54	80	50.2 (12.3)	107.0 (17.2)	38.7 (5.8)	112.4 (10.0)	
			Eloralintide 6–9 mg SC QW	24	79	49.1 (10.9)	110.0 (24.6)	40.3 (7.3)	116.9 (14.8)	
			Eloralintide 3–9 mg SC QW	52	79	48.7 (12.9)	110.8 (24.2)	39.7 (7.1)	115.8 (18.7)	
Dahl et al. 2025 [12]	NCT06064006; Phase 1b/2a; USA	Age 18–55 years; BMI 27.0–39.9 kg/m^2^	Amycretin 60 mg SC QW	17	59	42.4 (9.7)	89.4 (13.8)	31.5 (2.4)	NR	Part B: 36
			Placebo	5	60	33.6 (12.2)	83.6 (18.5)	30.0 (2.5)	NR	
			Amycretin 20 mg SC QW	34	59	40.2 (9.3)	90.4 (13.9)	32.0 (3.7)	NR	Part C: 36
			Placebo	5	40	28.6 (4.7)	89.4 (8.7)	30.8 (1.7)	NR	
			Amycretin 5 mg	16	63	36.1 (8.8)	92.9 (15.3)	32.8 (3.2)	NR	Part D: 28
			Placebo	4	100	32.5 (4.8)	88.3 (15.3)	32.5 (2.6)	NR	
			Amycretin 1.25 mg SC QW	16	25	38.9 (7.4)	99.1 (20.4)	32.2 (3.6)	NR	Part E: 20
			Placebo	4	50	27.8 (6.4)	96.1 (1.7)	33.1 (3.0)	NR	
Gasiorek et al. 2025 [13]	NCT05369390; Phase 1; USA	Age 18–55 years; BMI 25.0–34.9 kg/m^2^ (Part A & B), 27.0–39.9 kg/m^2^ (Part C & D)	Amycretin 50 mg PO QD	16	25	38 (10)	92.3 (10.8)	30.6 (2.5)	100.0 (7.7)	12
			Amycretin 2 × 50 mg PO QD	16	44	38 (8)	89.9 (9.8)	31.6 (2.8)	99.3 (7.5)	
			Amycretin 2 × 25 mg PO QD	16	75	37 (8)	89.3 (11.8)	32.8 (3.7)	97.1 (8.9)	
			Placebo PO QD	12	8	42 (4)	88.4 (7.8)	30.0 (1.4)	99.3 (7.3)	

Abbreviations: BMI, body mass index; PO, per oral; QD, once daily; QW, once weekly; SC, subcutaneous; USA, the United States of America; WC, waist circumference.

### 3.3 Risk of Bias in the Included Studies

Figure S1 presents domain‐specific and overall RoB assessments. Four trials (66.7%) were judged as having ‘some concerns’, primarily due to deviations from intended interventions [8, 12], missing outcome data [12] or concerns regarding selection of the reported result [8, 10, 11, 12]. Gasiorek et al. [13] had a high risk of bias for the selection of the reported result. The comparison‐adjusted funnel plot for percent change in body weight (Figure S2) appeared largely symmetrical, and Egger's test showed no evidence of small‐study effects (*p* = 0.9490). Nevertheless, we considered publication bias possible because the evidence base consists mainly of early‐phase, sponsor‐funded RCTs [18].

### 3.4 Percent Change in Body Weight

**FIGURE 2:**
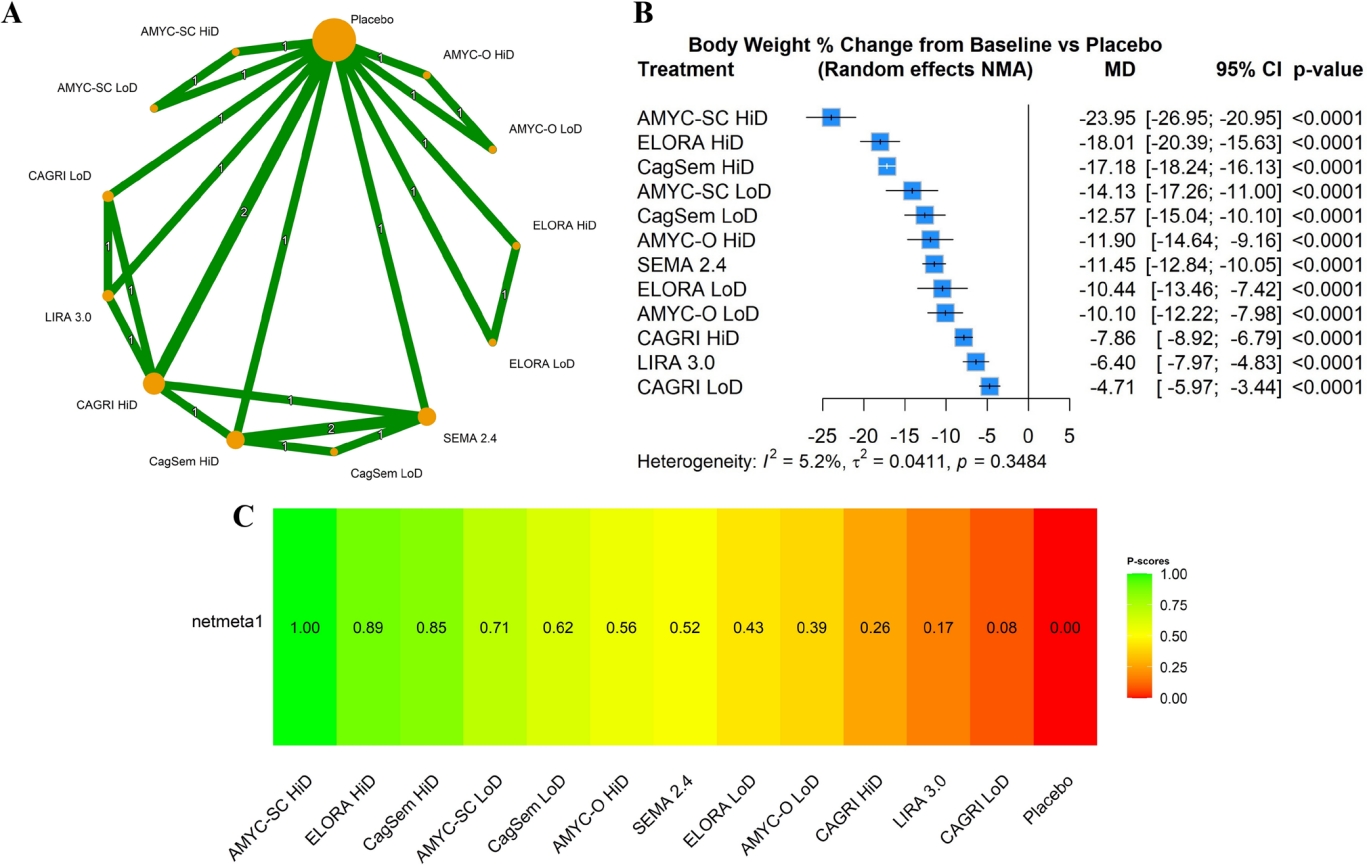
(A) Network diagram of treatment comparisons for percent change in body weight. This network meta‐analysis (NMA) graph illustrates comparisons among all treatments and placebo for the percent change in body weight from baseline. Each node represents a treatment. Lines between nodes indicate direct comparisons, with line thickness reflecting the number of supporting studies. Treatments without direct connections are compared through indirect evidence within the NMA framework. (B) Forest plot for the percent change in body weight. This figure presents the relative effects of treatments on the percent change in body weight from baseline. The forest plot displays mean differences (MDs) with 95% confidence intervals (CIs) for each treatment relative to the reference treatment (placebo). A negative MD indicates a greater percent reduction in body weight. (C) P score rankings for the percent change in body weight. The P score ranks treatments by their likelihood of being the most effective at reducing percent body weight, with higher P scores indicating greater effectiveness. AMYC‐O, oral amycretin; AMYC‐SC, subcutaneous amycretin; CAGRI, cagrilintide; CagSem, CagriSema; ELORA, eloralintide; HiD, high dose; LIRA, liraglutide; LoD, low dose; SEMA, semaglutide.

**FIGURE 3:**
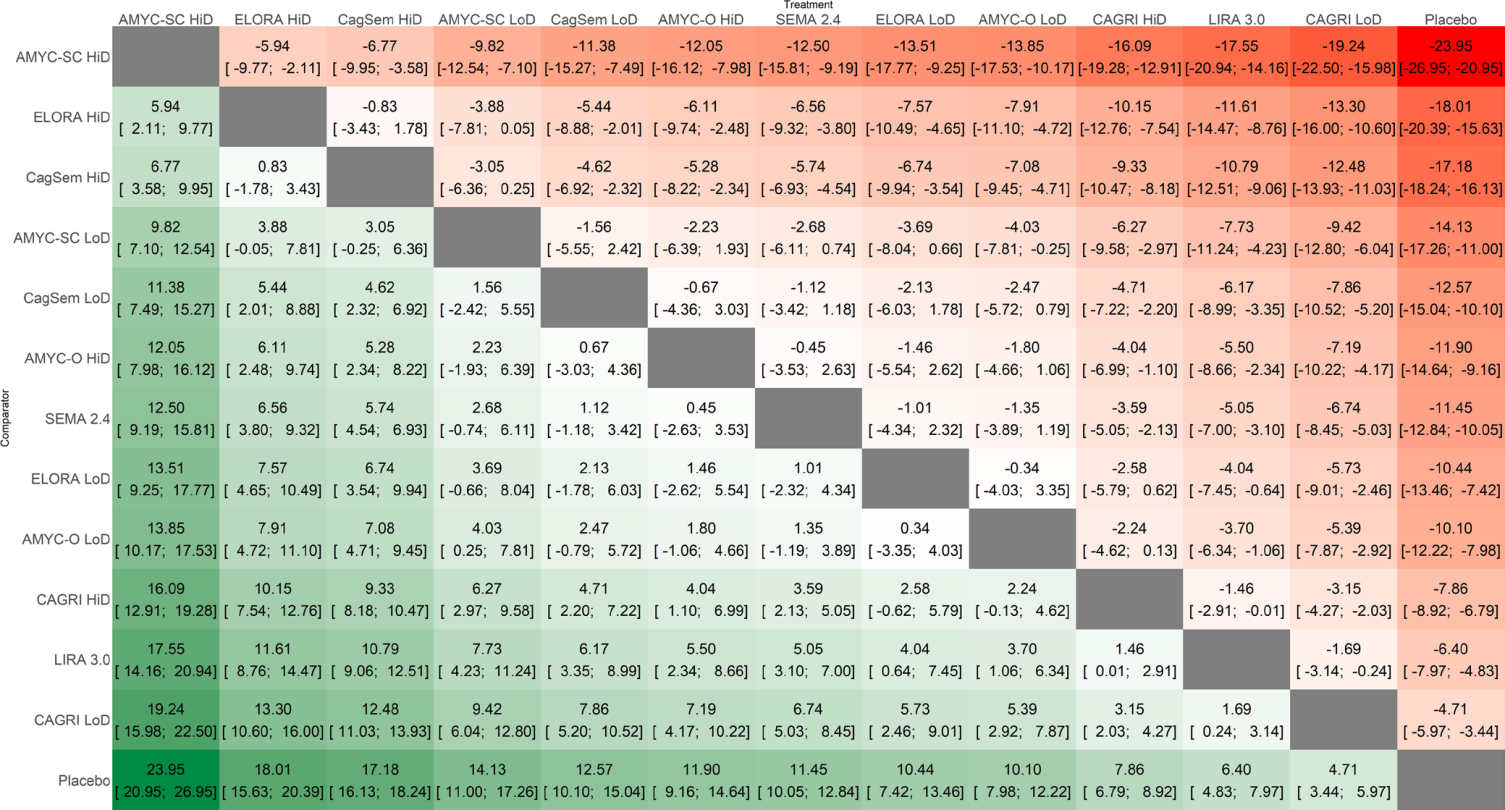
League table showing head‐to‐head comparisons among the interventions for percent changes in body weight. AMYC‐O, oral amycretin; AMYC‐SC, subcutaneous amycretin; CAGRI, cagrilintide; CagSem, CagriSema; ELORA, eloralintide; HiD, high dose; LIRA, liraglutide; LoD, low dose; SEMA, semaglutide.

Six trials (13 treatments, 24 pairwise comparisons) contributed to the NMA for percent change in body weight versus placebo (Figures 2A–C and 3). All ABTs produced greater weight loss than placebo. Heterogeneity and inconsistency were low (*τ* ^2^ = 0.0411; *τ* = 0.2027; *I* ^2^ = 5.2%), and *Q* tests indicated no significant heterogeneity. HiD SC amycretin achieved the greatest mean percent weight reduction versus placebo (MD −23.95%; P score 1.00), followed by HiD eloralintide (MD −18.01%; P score 0.89) and HiD CagriSema (MD −17.18%; P score 0.85). All ABTs except cagrilintide LoD produced larger weight reductions than liraglutide 3.0 mg (MD −6.4%; P score 0.17). HiD and LoD SC amycretin, HiD eloralintide, HiD and LoD CagriSema, and HiD oral amycretin all reduced body weight more than semaglutide 2.4 mg (MD −11.45%; P score 0.52). Netsplit analyses (Figure S3) showed no important inconsistency between direct and indirect evidence.

### 3.5 Absolute Change in Body Weight

Five trials (11 treatments, 21 pairwise comparisons) were included in the NMA of absolute weight change versus placebo (Figures S4 and S5). All ABTs yielded greater weight loss than placebo. Heterogeneity and inconsistency were low (*τ* ^2^ = 0.0356; *τ* = 0.1887; *I* ^2^ = 5.2%), with non‐significant *Q* tests. HiD eloralintide ranked highest for absolute weight loss (MD −19.44 kg; P score 0.98), followed by HiD CagriSema (MD −18.14 kg; P score 0.92) and LoD CagriSema (MD −13.48 kg; P score 0.77). All ABTs except LoD cagrilintide produced greater absolute weight loss than liraglutide 3.0 mg (−6.79 kg). HiD and LoD CagriSema and HiD eloralintide reduced weight more than semaglutide 2.4 mg (−12.03 kg). Netsplit results (Figure S6) indicated no meaningful inconsistency.

### 3.6 Change in Body Mass Index

Four trials (9 treatments, 15 pairwise comparisons) contributed to the NMA on BMI change versus placebo (Figures S7 and S8). All ABTs improved BMI more than the placebo. Heterogeneity and inconsistency were low (*τ* ^2^ = 0; *τ* = 0; *I* ^2^ = 0%), and *Q* tests were not significant. HiD eloralintide (MD −6.88 kg/m^2^, P score = 0.97) ranked highest, followed by HiD CagriSema (MD −6.5 kg/m^2^, P score = 0.89) and LoD CagriSema (MD −4.78 kg/m^2^, P score = 0.64). HiD and LoD CagriSema and HiD eloralintide all produced greater BMI reductions than semaglutide 2.4 mg. Netsplit analyses (Figure S9) showed no important inconsistency.

### 3.7 Change in Waist Circumference

Four trials (10 treatments, 18 pairwise comparisons) were included in the NMA for change in waist circumference versus placebo (Figures S10 and S11). All ABTs reduced waist circumference more than the placebo. Heterogeneity and inconsistency were moderate (*τ* ^2^ = 1.3573; *τ* = 1.1650; *I* ^2^ = 61.6%), but *Q* tests did not indicate statistically significant heterogeneity (total *Q* = 2.6; *p* = 0.1068). HiD eloralintide (MD −14.67 cm, P score = 0.98) ranked highest, followed by HiD CagriSema (MD −12.98 cm, P score = 0.91) and LoD eloralintide (MD −7.54 cm, P score = 0.60). HiD eloralintide and HiD CagriSema reduced waist circumference more than semaglutide 2.4 mg, and all ABTs except LoD cagrilintide outperformed liraglutide 3.0 mg. Netsplit analyses (Figure S12) did not reveal any important inconsistencies.

### 3.8 Body Weight Change ≥ 5%, ≥ 10% and ≥ 15%

Across the ≥ 5%, ≥ 10% and ≥ 15% weight‐loss thresholds, all active interventions were superior to placebo (Figures S13–S21). For ≥ 5% weight loss, HiD CagriSema (RR 2.94; P score 0.88) ranked highest, followed by HiD eloralintide (RR 3.02; P score 0.83) and LoD eloralintide (RR 2.69; P score 0.55). For ≥ 10% weight loss, HiD CagriSema (RR 5.95; P score 0.89) again ranked highest, with HiD eloralintide (RR 6.18; P score 0.87) and LoD eloralintide (RR 4.37; P score 0.56) next. At the ≥ 15% threshold, HiD CagriSema (RR 13.49; P score 0.99) remained most effective, while HiD eloralintide (RR 6.57; P score 0.66) and HiD cagrilintide (RR 6.00; P score 0.60) showed more modest but substantial effects. HiD CagriSema outperformed semaglutide 2.4 mg at all three thresholds. HiD eloralintide was superior to semaglutide 2.4 mg for ≥ 5% and ≥ 10% weight loss, but not clearly for ≥ 15%. Most ABTs, except the lowest‐dose regimens, exceeded liraglutide 3.0 mg. Netsplit analyses indicated no important inconsistency between direct and indirect estimates.

### 3.9 Gastrointestinal Adverse Events

GI AEs were generally more frequent with ABTs than with placebo, particularly for oral amycretin and HiD CagriSema. All active regimens except LoD SC amycretin, LoD cagrilintide and LoD eloralintide increased overall GI AEs relative to placebo (Figures S22–S24). HiD oral amycretin (RR 5.25; P score 0.04), LoD oral amycretin (RR 4.31; P score 0.13) and HiD CagriSema (RR 2.06; P score 0.20) had the highest risks and exceeded semaglutide 2.4 mg and liraglutide 3.0 mg. Nausea showed a similar pattern: all active treatments, except LoD SC amycretin and LoD eloralintide, increased the risk versus placebo (Figures S25–S27). HiD oral amycretin (RR 9.00; P score 0.10), HiD CagriSema (RR 4.45; P score 0.14) and LoD oral amycretin (RR 7.50; P score 0.18) conferred the highest risks and generally exceeded semaglutide 2.4 mg and liraglutide 3.0 mg. HiD CagriSema was the only regimen that significantly increased vomiting versus placebo (RR 8.95; P score 0.21), with a higher risk than liraglutide 3.0 mg and semaglutide 2.4 mg; HiD and LoD oral amycretin and HiD eloralintide showed numerically higher but imprecise risks (Figures S28–S30). In contrast, a significantly increased risk of diarrhoea was observed only with semaglutide 2.4 mg (RR 2.48), while estimates for ABTs had wide CIs that included no effect (Figures S31–S33). For constipation, HiD CagriSema (RR 2.71; P score 0.32) and HiD cagrilintide (RR 1.87; P score 0.67) were the only ABTs with significantly elevated risks versus placebo; HiD CagriSema had higher and HiD cagrilintide lower risks than liraglutide 3.0 mg and semaglutide 2.4 mg (Figures S34–S36).

### 3.10 Adverse Events Leading to Discontinuation

Compared with placebo, a significantly higher risk of AE‐related discontinuation was observed only with HiD CagriSema (RR 1.72), whereas all other treatments had imprecise estimates with CIs crossing the null (Figures S37 and S38). Netsplit analyses (Figure S39) showed no important inconsistency between direct and indirect effects.

### 3.11 CINeMA Certainty Assessment

The CINeMA evaluation for percent change in body weight (Figure S40) indicated that most pairwise comparisons were rated as low or very low confidence for mixed and indirect evidence, with a few comparisons rated as moderate. The main reasons for downgrading were within‐study bias and imprecision; in some comparisons, heterogeneity and incoherence also contributed. Across‐study bias and indirectness were generally judged as of low concern.

### 3.12 Sensitivity Analysis and Meta‐Regression

Given the sparse network and the fact that most studies contributed unique treatment comparisons, we did not conduct additional sensitivity analyses restricted to low‐RoB trials or to studies excluding those with imputed standard deviations, as this would have rendered several key comparisons inestimable. Because of the small number of trials per comparison, we did not perform formal meta‐regression or stratified NMA by baseline BMI or treatment duration; instead, these variables are considered qualitatively in interpreting the results.

## 4 Discussion

This NMA shows that long‐acting ABTs produce substantial weight loss in adults with overweight or obesity without diabetes, outperforming placebo across multiple anthropometric endpoints. Some Hi‐D amylin regimens achieved weight loss comparable to or greater than GLP‐1RAs, but these estimates are imprecise and should be viewed cautiously. SC amycretin HiD, eloralintide HiD and CagriSema HiD consistently appeared among the most effective regimens for percent and absolute weight loss, BMI reduction, waist‐circumference reduction, and achieving ≥ 5%, ≥ 10% and ≥ 15% weight‐loss thresholds, frequently exceeding semaglutide 2.4 mg and liraglutide 3.0 mg in the included trials. These benefits were accompanied by dose‐dependent increases in GI AEs, particularly with oral amycretin and HiD CagriSema, although only CagriSema HiD significantly increased discontinuations versus placebo. Statistical heterogeneity and inconsistency were generally low for weight outcomes, but CINeMA ratings were mostly very low or low certainty due to within‐study bias and imprecision, suggesting that comparative‐effect estimates should be used cautiously in clinical decision‐making. However, given the pharmacological differences, our results should be interpreted as comparing a class of ABTs rather than interchangeable ‘amylin agonists’.

The present findings suggest that long‐acting ABTs could occupy a prominent position near the top of the anti‐obesity pharmacotherapy hierarchy, especially for patients requiring large, durable weight reductions beyond those typically achievable with existing GLP‐1 RAs alone. HiD amycretin, eloralintide and CagriSema produced greater weight loss than semaglutide 2.4 mg and liraglutide 3.0 mg, indicating that amylin‐based approaches, either alone or in combination with GLP‐1 receptor agonism, may be a next step for individuals with an inadequate response or intolerance to GLP‐1 monotherapy. Given the dose‐dependent GI AEs observed, careful dose titration, gradual uptitration schedules, and early management of nausea, vomiting and constipation will be essential to optimize tolerability and adherence in clinical practice, particularly for oral amycretin and high‐dose CagriSema [23].

From a practical standpoint, once‐weekly subcutaneous formulations (cagrilintide, eloralintide, amycretin, CagriSema) align with current injection practices for GLP‐1 RAs, making them easier to integrate into existing obesity treatment pathways. Conversely, if proven safe and effective in later studies, oral amycretin could broaden access for patients hesitant to use injections. The strong effects on body weight, BMI and waist circumference suggest that ABTs might be suitable for high‐risk groups, including those with severe obesity, obesity‐related cardiometabolic conditions, or those preparing for bariatric surgery, though these applications require further research. Our estimates, however, focus on short‐ to medium‐term weight loss and should not be directly compared with the 52–60‐week weight plateaus typical of established anti‐obesity medications. Moreover, because of pronounced dose–response relationships and ongoing dose‐finding phases in some programs, current treatment rankings and comparisons are based on doses studied to date and may not fully reflect the efficacy of future phase 3 or marketed regimens. At present, most evidence comes from early‐phase, sponsor‐funded RCTs with short‐ to intermediate‐term follow‐ups, so guideline panels and clinicians should interpret these results as promising but preliminary when considering ABTs alongside established GLP‐1 and dual incretin therapies.

Several priorities for future research on amylin‐based anti‐obesity therapies emerge. First, large, independent phase 3 trials with extended follow‐up are necessary to confirm the extent and durability of weight loss, clarify dose–response relationships, and better understand long‐term safety, including rare adverse events and off‐target effects. Second, RCTs in populations with obesity‐related comorbidities, such as T2D, cardiovascular disease, non‐alcoholic steatohepatitis and obesity‐related sleep apnea, are needed to determine whether weight loss translates into meaningful improvements in cardiometabolic and hepatic outcomes, mirroring the cardiovascular outcome trials conducted for GLP‐1 receptor agonists. Head‐to‐head trials comparing different amylin‐based modalities (selective amylin agonists, dual amylin‐calcitonin agonists, amylin‐GLP‐1 co‐agonists, and fixed‐dose combinations) will be important for delineating the optimal balance among efficacy, tolerability, convenience and cost. Mechanistic studies interrogating central and peripheral signalling pathways, energy expenditure, and changes in body composition may help explain inter‐individual variability in responses and guide personalized treatment strategies. Finally, real‐world implementation research, pharmacoeconomic analyses, assessment of patient‐reported outcomes, and cost‐effectiveness analyses will be crucial for defining the role of amylin‐based therapies within chronic obesity care models, particularly in health systems with constrained resources and limited access to high‐cost injectable drugs.

### 4.1 Strengths and Limitations of the Study

This NMA has several strengths, including a comprehensive search across multiple databases and registries up to March 2026, rigorous application of PICOS‐based eligibility criteria, and inclusion of a wide range of long‐acting ABTs across early to late‐phase RCTs in adults with overweight or obesity without diabetes. Using a frequentist random‐effects network meta‐analysis framework enabled simultaneous comparison and ranking of multiple doses and formulations against placebo and active comparators, with generally low statistical heterogeneity and no significant inconsistency in net‐split analyses for key weight outcomes. The pre‐specified focus on anthropometric endpoints and GI AEs, along with formal assessment of publication bias and CINeMA‐based grading of evidence certainty, offers a structured and transparent summary of the current data landscape. However, the analysis is limited by the small number of available RCTs, the predominance of early‐phase, industry‐sponsored trials, and short follow‐up periods (12–68 weeks), which restrict conclusions regarding long‐term effectiveness, safety and weight‐regain trajectories. Several comparisons had low event counts or wide confidence intervals, leading to CINeMA ratings of low or very low certainty due to within‐study bias and imprecision, especially for safety outcomes and less common dose regimens. In addition, the evidence base is sparse at the study level, with many treatment contrasts informed by single trials, and indirect comparisons sometimes linking trials that differ in baseline BMI and treatment duration; as a result, strict transitivity assumptions may not be fully met, and our findings should be interpreted as hypothesis‐generating rather than definitive. Clinical and methodological heterogeneity, including differences in trial phase, inclusion criteria, background lifestyle interventions, dose‐titration schedules, and outcome ascertainment, may not be fully captured by statistical models. In addition, the limited number of studies per comparison precluded robust meta‐regression or sensitivity analyses (e.g., restricting to low RoB studies or excluding trials with imputed variability measures) without disconnecting the network. We included trials with at least one arm using an amylin‐based therapy. RCTs comparing only established anti‐obesity agents, like liraglutide vs. semaglutide without an amylin arm, were excluded, as they address different questions and are covered in other NMAs. Our network is part of, and complements, broader NMAs of obesity drugs, not a comprehensive comparison of all approved treatments. Additionally, the analysis was limited to individuals without diabetes, as the presence of diabetes mellitus may alter weight‐loss responses due to baseline metabolic differences and the concurrent use of glucose‐lowering therapies. Excluding diabetes RCTs in this NMA reduces clinical heterogeneity and offers a clearer assessment of the anti‐obesity effects of amylin‐based therapies; however, it limits its applicability to the broader population with obesity and coexisting metabolic conditions.

## 5 Conclusions

In adults with overweight or obesity without diabetes, long‐acting ABTs were associated with numerically greater weight loss than placebo, and some ABTs achieved greater weight loss than the GLP‐1RA regimens studied in this NMA, but they were also associated with more frequent AEs, mainly GI, at higher doses. However, these estimates are based on a small number of heterogeneous, mostly early‐phase trials, and the certainty of evidence for most comparisons is low or very low. Taken together, our findings suggest that amylin‐based agents are promising but still experimental candidates for pharmacologic obesity treatment and should be viewed as hypothesis‐generating signals that require confirmation in larger, longer‐term, and methodologically robust Phase 3 studies.

## Author Contributions

**A. B. M. Kamrul‐Hasan:** conceptualization, methodology, software, formal analysis, data curation, supervision, resources, project administration, visualization, writing – original draft, investigation, validation. **Ibrahim Khalil:** data curation, software, formal analysis, resources, writing – original draft. **Kunal Mahajan:** investigation, visualization, resources, methodology, writing – original draft. **Deep Dutta:** conceptualization, writing – review and editing, methodology, validation, resources. **Mainak Banerjee:** writing – review and editing, methodology, data curation, resources, investigation. **Joseph M. Pappachan:** validation, writing – review and editing, project administration, resources, supervision.

## Funding

The authors have nothing to report.

## Conflicts of Interest

The authors declare no conflicts of interest.

## Supporting information


**Table S1:** Search strings.
**Table S2:** Results from individual studies' efficacy outcomes.
**Table S3:** Results from individual studies' safety outcomes.
**Figure S1:** (A) Risk of bias summary: Review authors' judgements about each risk of bias item for each included study; (B) risk of bias graph: Review authors' judgements about each risk of bias item presented as percentages across all included studies.
**Figure S2:** Comparison‐adjusted funnel plots for the interventions for the percent change in body weight.
**Figure S3:** Netsplit estimates for separate direct and indirect evidence for the percent change in body weight.
**Figure S4:** Network diagram (A), network meta‐analysis forest plot (B), P score (C) for the absolute change in body weight, comparing various amylin‐based therapies to placebo.
**Figure S5:** League table showing head‐to‐head comparisons among the interventions for absolute changes in body weight (kg).
**Figure S6:** Netsplit estimates for separate direct and indirect evidence for absolute changes in body weight (kg).
**Figure S7:** Network diagram (A), network meta‐analysis forest plot (B), P score (C) for the change in body mass index, comparing various amylin‐based therapies to placebo.
**Figure S8:** League table showing head‐to‐head comparisons among the interventions for changes in body mass index.
**Figure S9:** Netsplit estimates for separate direct and indirect evidence for changes in body mass index.
**Figure S10:** Network diagram (A), network meta‐analysis forest plot (B), P score (C) for the change in waist circumference, comparing various amylin‐based therapies to placebo.
**Figure S11:** League table showing head‐to‐head comparisons among the interventions for changes in waist circumference.
**Figure S12:** Netsplit estimates for separate direct and indirect evidence for changes in waist circumference.
**Figure S13:** Network diagram (A), network meta‐analysis forest plot (B), P score (C) for the proportions of study subjects who lost ≥ 5% of their baseline body weight, comparing various amylin‐based therapies to placebo.
**Figure S14:** League table showing head‐to‐head comparisons among the interventions for the proportions of study subjects who lost ≥ 5% of their baseline body weight.
**Figure S15:** Netsplit estimates for separate direct and indirect evidence for the proportions of study subjects who lost ≥ 5% of their baseline body weight.
**Figure S16:** Network diagram (A), network meta‐analysis forest plot (B), P score (C) for the proportions of study subjects who lost ≥ 10% of their baseline body weight, comparing various amylin‐based therapies to placebo.
**Figure S17:** League table showing head‐to‐head comparisons among the interventions for the proportions of study subjects who lost ≥ 10% of their baseline body weight.
**Figure S18:** Netsplit estimates for separate direct and indirect evidence for the proportions of study subjects who lost ≥ 10% of their baseline body weight.
**Figure S19:** Network diagram (A), network meta‐analysis forest plot (B), P score (C) for the proportions of study subjects who lost ≥ 15% of their baseline body weight, comparing various amylin‐based therapies to placebo.
**Figure S20:** League table showing head‐to‐head comparisons among the interventions for the proportions of study subjects who lost ≥ 15% of their baseline body weight.
**Figure S21:** Netsplit estimates for separate direct and indirect evidence for the proportions of study subjects who lost ≥ 15% of their baseline body weight.
**Figure S22:** Network diagram (A), network meta‐analysis forest plot (B), P score (C) for the proportions of study subjects who experienced gastrointestinal adverse events, comparing various amylin‐based therapies to placebo.
**Figure S23:** League table showing head‐to‐head comparisons among the interventions for the proportions of study subjects who experienced gastrointestinal adverse events, comparing various amylin‐based therapies to placebo.
**Figure S24:** Netsplit estimates for separate direct and indirect evidence for the proportions of study subjects who experienced gastrointestinal adverse events, comparing various amylin‐based therapies to placebo.
**Figure S25:** Network diagram (A), network meta‐analysis forest plot (B), P score (C) for the proportions of study subjects who experienced nausea, comparing various amylin‐based therapies to placebo.
**Figure S26:** League table showing head‐to‐head comparisons among the interventions for the proportions of study subjects who experienced nausea.
**Figure S27:** Netsplit estimates for separate direct and indirect evidence for the proportions of study subjects who experienced nausea.
**Figure S28:** Network diagram (A), network meta‐analysis forest plot (B), P score (C) for the proportions of study subjects who experienced vomiting, comparing various amylin‐based therapies to placebo.
**Figure S29:** League table showing head‐to‐head comparisons among the interventions for the proportions of study subjects who experienced vomiting.
**Figure S30:** Netsplit estimates for separate direct and indirect evidence for the proportions of study subjects who experienced vomiting.
**Figure S31:** Network diagram (A), network meta‐analysis forest plot (B), P score (C) for the proportions of study subjects who experienced diarrhoea, comparing various amylin‐based therapies to placebo.
**Figure S32:** League table showing head‐to‐head comparisons among the interventions for the proportions of study subjects who experienced diarrhoea.
**Figure S33:** Netsplit estimates for separate direct and indirect evidence for the proportions of study subjects who experienced diarrhoea.
**Figure S34:** Network diagram (A), network meta‐analysis forest plot (B), P score (C) for the proportions of study subjects who experienced constipation, comparing various amylin‐based therapies to placebo.
**Figure S35:** League table showing head‐to‐head comparisons among the interventions for the proportions of study subjects who experienced constipation.
**Figure S36:** Netsplit estimates for separate direct and indirect evidence for the proportions of study subjects who experienced constipation.
**Figure S37:** Network diagram (A), network meta‐analysis forest plot (B), P score (C) for the proportions of study subjects with adverse events leading to discontinuation, comparing various amylin‐based therapies to placebo.
**Figure S38:** League table showing head‐to‐head comparisons among the interventions for the proportions of study subjects with adverse events leading to discontinuation.
**Figure S39:** Netsplit estimates for separate direct and indirect evidence for the proportions of study subjects with adverse events leading to discontinuation.
**Figure S40:** CINeMA confidence rating for the network meta‐analysis of percent change in body weight. Most pairwise comparisons were rated as low or very low confidence, primarily due to within‐study bias, imprecision and heterogeneity. A limited number of comparisons achieved moderate confidence.

## Data Availability

Every dataset generated or scrutinized during this research is provided within this published article and its Supporting Information.
