# Supplementary material for: Novel Amylin‐Based Therapies for Weight Management in Adults With Overweight or Obesity Without Diabetes: A Network Meta‐Analysis
Source: Endocrinol Diabetes Metab. 2026 May 22;9(3):e70247. doi: 10.1002/edm2.70247 (PMC13240113; doi:10.1002/edm2.70247)
Supplement: Supplementary file 1 — Table S1: Search strings. Table S2: Results from individual studies' efficacy outcomes. Table S3: Results from individual studies' safety outcomes. Figure S1: (A) Risk of bias summary: Review authors' judgements about each risk of bias item for each included study; (B) risk of bias graph: Review authors' judgements about each risk of bias item presented as percentages across all included studies. Figure S2: Comparison‐adjusted funnel plots for the interventions for the percent change in body weight. Figure S3: Netsplit estimates for separate direct and indirect evidence for the percent change in body weight. Figure S4: Network diagram (A), network meta‐analysis forest plot (B), P score (C) for the absolute change in body weight, comparing various amylin‐based therapies to placebo. Figure S5: League table showing head‐to‐head comparisons among the interventions for absolute changes in body weight (kg). Figure S6: Netsplit estimates for separate direct and indirect evidence for absolute changes in body weight (kg). Figure S7: Network diagram (A), network meta‐analysis forest plot (B), P score (C) for the change in body mass index, comparing various amylin‐based therapies to placebo. Figure S8: League table showing head‐to‐head comparisons among the interventions for changes in body mass index. Figure S9: Netsplit estimates for separate direct and indirect evidence for changes in body mass index. Figure S10: Network diagram (A), network meta‐analysis forest plot (B), P score (C) for the change in waist circumference, comparing various amylin‐based therapies to placebo. Figure S11: League table showing head‐to‐head comparisons among the interventions for changes in waist circumference. Figure S12: Netsplit estimates for separate direct and indirect evidence for changes in waist circumference. Figure S13: Network diagram (A), network meta‐analysis forest plot (B), P score (C) for the proportions of study subjects who lost ≥ 5% of their baseline body weight, comparing var [file EDM2-9-e70247-s001.zip › edm270247-sup-0002-TableS1-S3-FigureS1-S39@Supplementary Files.docx]

**Table S1.** Search strings

| **Database** | **Search strings** |
| --- | --- |
| **PubMed** | ("Obesity"[Mesh] OR "Overweight"[Mesh] OR obesity[tiab] OR obese[tiab] OR overweight[tiab] OR "weight management"[tiab] OR "body weight"[tiab] OR "weight loss"[tiab])  AND  ("Islet Amyloid Polypeptide"[Mesh] OR amylin[tiab] OR "islet amyloid polypeptide"[tiab] OR IAPP[tiab] OR "amylin receptor agonist*"[tiab] OR "amylin agonist*"[tiab] OR "amylin analog*"[tiab] OR "amylin-based therap*"[tiab] OR "long-acting amylin analog*"[tiab] OR "long-acting amylin agonist*"[tiab] OR "dual amylin-calcitonin receptor agonist*"[tiab] OR "dual amylin GLP-1 receptor agonist*"[tiab] OR cagrilintide[tiab] OR AM833[tiab] OR "cagrilintide-semaglutide"[tiab] OR CagriSema[tiab] OR amycretin[tiab] OR eloralintide[tiab] OR LY3841136[tiab] OR petrelintide[tiab] OR ZP8396[tiab] OR AZD6234[tiab] OR "MET-233"[tiab] OR MET233[tiab] OR NN1213[tiab] OR "Amylin-355"[tiab] OR NN9638[tiab] OR "KBP-042"[tiab] OR "KBP-066"[tiab] OR "KBP-066A"[tiab] OR "KBP-088"[tiab] OR "KBP-088A"[tiab] OR "KBP-089"[tiab] OR ASC36[tiab] OR GUB014295[tiab]) |
| **Scopus** | TITLE-ABS-KEY(obesity OR obese OR overweight OR "weight management" OR "body weight" OR "weight loss")  AND  TITLE-ABS-KEY(amylin OR "islet amyloid polypeptide" OR IAPP OR "amylin receptor agonist*" OR "amylin agonist*" OR "amylin analog*" OR "amylin-based therap*" OR "long-acting amylin analog*" OR "long-acting amylin agonist*" OR "dual amylin-calcitonin receptor agonist*" OR "dual amylin GLP-1 receptor agonist*" OR cagrilintide OR AM833 OR "cagrilintide-semaglutide" OR CagriSema OR amycretin OR eloralintide OR LY3841136 OR petrelintide OR ZP8396 OR AZD6234 OR "MET-233" OR MET233 OR NN1213 OR "Amylin-355" OR NN9638 OR "KBP-042" OR "KBP-066" OR "KBP-066A" OR "KBP-088" OR "KBP-088A" OR "KBP-089" OR ASC36 OR GUB014295) |
| **Web of Science** | TS=(obesity OR obese OR overweight OR "weight management" OR "body weight" OR "weight loss")  AND  TS=(amylin OR "islet amyloid polypeptide" OR IAPP OR "amylin receptor agonist*" OR "amylin agonist*" OR "amylin analog*" OR "amylin-based therap*" OR "long-acting amylin analog*" OR "long-acting amylin agonist*" OR "dual amylin-calcitonin receptor agonist*" OR "dual amylin GLP-1 receptor agonist*" OR cagrilintide OR AM833 OR "cagrilintide-semaglutide" OR CagriSema OR amycretin OR eloralintide OR LY3841136 OR petrelintide OR ZP8396 OR AZD6234 OR "MET-233" OR MET233 OR NN1213 OR "Amylin-355" OR NN9638 OR "KBP-042" OR "KBP-066" OR "KBP-066A" OR "KBP-088" OR "KBP-088A" OR "KBP-089" OR ASC36 OR GUB014295) |

**Table S2.** Results from individual studies’ efficacy outcomes

| **Study** | **Treatment details** | **N** | **BW (%)** | | **BW (kg)** | | **BMI (kg/m^2^)** | | **WC (cm)** | | **≥5% Weight loss** | | **≥10% Weight loss** | | **≥15% Weight loss** | |
| --- | --- | --- | --- | --- | --- | --- | --- | --- | --- | --- | --- | --- | --- | --- | --- | --- |
|  |  |  | Mean | SD | Mean | SD | Mean | SD | Mean | SD | N | N | N | N | N | N |
| Lau et al. 2021 | Cagrilintide 0.3 mg | 101 | -6 | 6 | -6.4 | 6 | - | - | -5.8 | 7 | 101 | 59 | 101 | 15 | 101 | 3 |
|  | Cagrilintide 0.6 mg | 100 | -6.8 | 6 | -7.1 | 6 | - | - | -6 | 7 | 100 | 62 | 100 | 24 | 100 | 5 |
|  | Cagrilintide 1.2 mg | 102 | -9.1 | 7.1 | -9.7 | 7.1 | - | - | -7.9 | 7 | 102 | 78 | 102 | 37 | 102 | 15 |
|  | Cagrilintide 2.4 mg | 102 | -9.7 | 6 | -10.3 | 6 | - | - | -8.6 | 7 | 102 | 75 | 102 | 45 | 102 | 22 |
|  | Cagrilintide 4.5 mg | 101 | -10.8 | 6 | -11.5 | 6 | - | - | -9.2 | 7 | 101 | 90 | 101 | 55 | 101 | 19 |
|  | Liraglutide 3.0 mg | 99 | -9 | 6 | -9.6 | 6 | - | - | -7.8 | 7 | 99 | 75 | 99 | 39 | 99 | 14 |
|  | Placebo | 101 | -3 | 6 | -3.3 | 6 | - | - | -4.4 | 7 | 101 | 31 | 101 | 10 | 101 | 3 |
| Garvey et al. 2025 | CagriSema 2.4/2.4 mg | 2108 | -20.4 | 12.5 | -21.6 | 9 | -7.7 | 2.8 | -17.5 | 17 | 2108 | 1937 | 2108 | 1761 | 2108 | 1479 |
|  | Semaglutide 2.4 mg | 302 | -14.9 | 10.1 | -15.6 | 9 | -5.6 | 3.1 | -13.4 | 13 | 302 | 254 | 302 | 209 | 302 | 142 |
|  | Cagrilintide 2.4 mg | 302 | -11.5 | 10.1 | -12.2 | 9 | -4.4 | 3.3 | -10.6 | 13 | 302 | 238 | 302 | 167 | 302 | 94 |
|  | Placebo | 705 | -3 | 12.8 | -3.3 | 9 | -1.2 | 2.4 | -4 | 17 | 705 | 222 | 705 | 101 | 705 | 37 |
| Enebo et al. 2021 | CagriSema 0.16/2.4 mg | 12 | -8.3 | 5.5 | -8 | 5.2 | -2.6 | 4.9 | - | - | - | - | - | - | - | - |
|  | CagriSema 0.3/2.4 mg | 12 | -10 | 5.2 | -9.3 | 4.9 | -3 | 3.8 | - | - | - | - | - | - | - | - |
|  | CagriSema 0.6/2.4 mg | 12 | -10.6 | 5.9 | -10.1 | 5.5 | -3.7 | 5.5 | - | - | - | - | - | - | - | - |
|  | CagriSema 1.2/2.4 mg | 12 | -15.7 | 5.5 | -14.6 | 5.2 | -4.9 | 4.2 | - | - | - | - | - | - | - | - |
|  | CagriSema 2.4/2.4 mg | 12 | -17.1 | 5.2 | -15.9 | 4.9 | -5.6 | 5.9 | - | - | - | - | - | - | - | - |
|  | CagriSema 4.5/2.4 mg | 11 | -15.4 | 4.3 | -15 | 4.3 | -5 | 4 | - | - | - | - | - | - | - | - |
|  | Placebo + Semaglutide 2.4 mg | 24 | -9.5 | 4.9 | -8.7 | 4.9 | -3 | 9.8 | - | - | - | - | - | - | - | - |
| Billings et al. 2025 | Placebo | 53 | -0.4 | 6.5 | -0.2 | 7.5 | -0.2 | 2.8 | -1.1 | 7.8 | 53 | 16 | 53 | 7 | 53 | 5 |
|  | Eloralintide 1 mg | 28 | -9.5 | 9.7 | -10.2 | 8.8 | -3.7 | 3 | -7.3 | 9.6 | 28 | 20 | 28 | 15 | 28 | 9 |
|  | Eloralintide 3 mg | 24 | -12.4 | 7.9 | -13.3 | 6.8 | -4.7 | 2.3 | -10.2 | 7.7 | 24 | 21 | 24 | 15 | 24 | 8 |
|  | Eloralintide 6 mg | 28 | -17.6 | 9.5 | -18.7 | 9.6 | -6.7 | 3.4 | -15.8 | 8.3 | 28 | 24 | 28 | 22 | 28 | 17 |
|  | Eloralintide 9 mg | 54 | -20.1 | 10.3 | -21.3 | 10.5 | -7.7 | 3.8 | -16.3 | 9.5 | 54 | 50 | 54 | 44 | 54 | 38 |
|  | Eloralintide 6–9 mg | 24 | -19.9 | 8.1 | -21 | 7.2 | -7.8 | 2.9 | -17.1 | 7.2 | 24 | 23 | 24 | 22 | 24 | 16 |
|  | Eloralintide 3–9 mg | 52 | -16.4 | 10.7 | -17.8 | 9.7 | -6.3 | 3.3 | -14.6 | 8.8 | 52 | 47 | 52 | 41 | 52 | 27 |
| Dahl et al. 2025 | Amycretin 60 mg | 17 | -24.3 | 7.8 | - | - | - | - | - | - | - | - | - | - | - | - |
|  | Placebo | 5 | -1.1 | 6.7 | - | - | - | - | - | - | - | - | - | - | - | - |
|  | Amycretin 20 mg | 34 | -22 | 5.8 | - | - | - | - | - | - | - | - | - | - | - | - |
|  | Placebo | 5 | 1.9 | 5.6 | - | - | - | - | - | - | - | - | - | - | - | - |
|  | Amycretin 5 mg | 16 | -16.2 | 4.4 | - | - | - | - | - | - | - | - | - | - | - | - |
|  | Placebo | 4 | 2.3 | 3.6 | - | - | - | - | - | - | - | - | - | - | - | - |
|  | Amycretin 1.25 mg | 16 | -9.7 | 5.2 | - | - | - | - | - | - | - | - | - | - | - | - |
|  | Placebo | 4 | 2 | 4.8 | - | - | - | - | - | - | - | - | - | - | - | - |
| Gasiorek et al. 2025 | Amycretin 50 mg | 16 | -10.4 | 4.6 | -9.5 | 4.2 | -3.1 | 1.3 | -9.5 | 6.8 | - | - | - | - | - | - |
|  | Amycretin 2 x 50 mg | 16 | -13.1 | 4.8 | -11.9 | 4.5 | -4.1 | 1.4 | -8.8 | 5.3 | - | - | - | - | - | - |
|  | Amycretin 2 x 25 mg | 16 | -12.2 | 4.5 | -11.1 | 4.3 | -4.1 | 1.6 | -7.3 | 5.7 | - | - | - | - | - | - |
|  | Placebo | 12 | -1.2 | 2.4 | -1.1 | 2.1 | -0.4 | 0.7 | -2.5 | 3.6 | - | - | - | - | - | - |

**Table S3.** Results from individual studies’ safety outcomes

| **Study** | **Treatment details** | **N** | **GI AEs** | | **Nausea** | | **Vomiting** | | **Diarrhea** | | **Constipation** | | **Treatment discontinuation for AEs** | |
| --- | --- | --- | --- | --- | --- | --- | --- | --- | --- | --- | --- | --- | --- | --- |
|  |  |  | **N** | **n** | **N** | **n** | **N** | **n** | **N** | **n** | **N** | **n** | **N** | **n** |
| Lau et al. 2021 | Cagrilintide 0.3 mg | 101 | 101 | 41 | 101 | 20 | 101 | 6 | 101 | 15 | 101 | 11 | 101 | 2 |
|  | Cagrilintide 0.6 mg | 100 | 100 | 45 | 100 | 27 | 100 | 6 | 100 | 10 | 100 | 9 | 100 | 4 |
|  | Cagrilintide 1.2 mg | 102 | 102 | 49 | 102 | 37 | 102 | 5 | 102 | 8 | 102 | 8 | 102 | 6 |
|  | Cagrilintide 2.4 mg | 102 | 102 | 52 | 102 | 32 | 102 | 9 | 102 | 18 | 102 | 17 | 102 | 6 |
|  | Cagrilintide 4.5 mg | 101 | 101 | 64 | 101 | 47 | 101 | 8 | 101 | 7 | 101 | 21 | 101 | 1 |
|  | Liraglutide 3.0 mg | 99 | 99 | 59 | 99 | 39 | 99 | 20 | 99 | 18 | 99 | 26 | 99 | 7 |
|  | Placebo | 101 | 101 | 32 | 101 | 18 | 101 | 3 | 101 | 9 | 101 | 7 | 101 | 3 |
| Garvey et al. 2025 | CagriSema 2.4/2.4 mg | 2108 | 2106 | 1676 | 2106 | 1159 | 2106 | 549 | 2106 | 517 | 2106 | 646 | 2106 | 125 |
|  | Semaglutide 2.4 mg | 302 | 302 | 223 | 302 | 132 | 302 | 70 | 302 | 79 | 302 | 84 | 302 | 11 |
|  | Cagrilintide 2.4 mg | 302 | 302 | 163 | 302 | 72 | 302 | 21 | 302 | 46 | 302 | 62 | 302 | 8 |
|  | Placebo | 705 | 705 | 281 | 705 | 89 | 705 | 29 | 705 | 85 | 705 | 82 | 705 | 25 |
| Enebo et al. 2021 | CagriSema 0.16/2.4 mg | 12 | 12 | 7 | 12 | 6 | 12 | 0 | 12 | 0 | - | - | 12 | 1 |
|  | CagriSema 0.3/2.4 mg | 12 | 12 | 10 | 12 | 9 | 12 | 4 | 12 | 2 | - | - | 12 | 0 |
|  | CagriSema 0.6/2.4 mg | 12 | 12 | 7 | 12 | 6 | 12 | 2 | 12 | 2 | - | - | 12 | 0 |
|  | CagriSema 1.2/2.4 mg | 12 | 12 | 10 | 12 | 6 | 12 | 1 | 12 | 1 | - | - | 12 | 1 |
|  | CagriSema 2.4/2.4 mg | 12 | 12 | 11 | 12 | 10 | 12 | 9 | 12 | 2 | - | - | 12 | 0 |
|  | CagriSema 4.5/2.4 mg | 11 | 11 | 9 | 11 | 8 | 11 | 4 | 11 | 0 | - | - | 11 | 0 |
|  | Placebo + Semaglutide 2.4 mg | 24 | 24 | 19 | 24 | 14 | 24 | 3 | 24 | 9 | - | - | 24 | 0 |
| Billings et al. 2025 | Placebo | 53 | 52 | 20 | 52 | 7 | 52 | 0 | 52 | 5 | 52 | 3 | 52 | 4 |
|  | Eloralintide 1 mg | 28 | 27 | 10 | 27 | 3 | 27 | 0 | 27 | 1 | 27 | 4 | 27 | 3 |
|  | Eloralintide 3 mg | 24 | 23 | 9 | 23 | 3 | 23 | 0 | 23 | 2 | 23 | 4 | 23 | 0 |
|  | Eloralintide 6 mg | 28 | 28 | 23 | 28 | 18 | 28 | 7 | 28 | 10 | 28 | 2 | 28 | 6 |
|  | Eloralintide 9 mg | 54 | 54 | 33 | 54 | 18 | 54 | 6 | 54 | 6 | 54 | 13 | 54 | 4 |
|  | Eloralintide 6–9 mg | 24 | 24 | 16 | 24 | 13 | 24 | 3 | 24 | 3 | 24 | 4 | 24 | 2 |
|  | Eloralintide 3–9 mg | 52 | 52 | 28 | 52 | 13 | 52 | 1 | 52 | 9 | 52 | 4 | 52 | 6 |
| Dahl et al. 2025 | Amycretin 60 mg | 17 | 17 | 16 | 17 | 14 | 17 | 8 | 17 | 7 | 17 | 2 | 17 | 6 |
|  | Placebo | 5 | 5 | 4 | 5 | 3 | 5 | 3 | 5 | 1 | 5 | 1 | 5 | 0 |
|  | Amycretin 20 mg | 34 | 34 | 32 | 34 | 27 | 34 | 18 | 34 | 11 | 34 | 15 | 34 | 7 |
|  | Placebo | 5 | 5 | 3 | 5 | 2 | 5 | 1 | 5 | 1 | 5 | 1 | 5 | 1 |
|  | Amycretin 5 mg | 16 | 16 | 15 | 16 | 12 | 16 | 4 | 16 | 4 | 16 | 2 | 16 | 1 |
|  | Placebo | 4 | 4 | 1 | 4 | 1 | 4 | 0 | 4 | 0 | 4 | 0 | 4 | 1 |
|  | Amycretin 1.25 mg | 16 | 16 | 10 | 16 | 8 | 16 | 5 | 16 | 4 | 16 | 2 | 16 | 0 |
|  | Placebo | 4 | 4 | 2 | 4 | 2 | 4 | 1 | 4 | 1 | 4 | 0 | 4 | 0 |
| Gasiorek et al. 2025 | Amycretin 50 mg | 16 | 16 | 8 | 16 | 5 | 16 | 6 | 16 | 1 | 16 | 2 | 16 | 0 |
|  | Amycretin 2 x 50 mg | 16 | 16 | 14 | 16 | 12 | 16 | 9 | 16 | 3 | 16 | 4 | 16 | 0 |
|  | Amycretin 2 x 25 mg | 16 | 16 | 15 | 16 | 15 | 16 | 10 | 16 | 4 | 16 | 7 | 16 | 1 |
|  | Placebo | 12 | 12 | 2 | 12 | 1 | 12 | 0 | 12 | 0 | 12 | 1 | 12 | 0 |

**Figure S1. A. Risk of bias summary:** Review authors’ judgments about each risk of bias item for each included study; **B. Risk of bias graph:** Review authors’ judgments about each risk of bias item presented as percentages across all included studies.

**
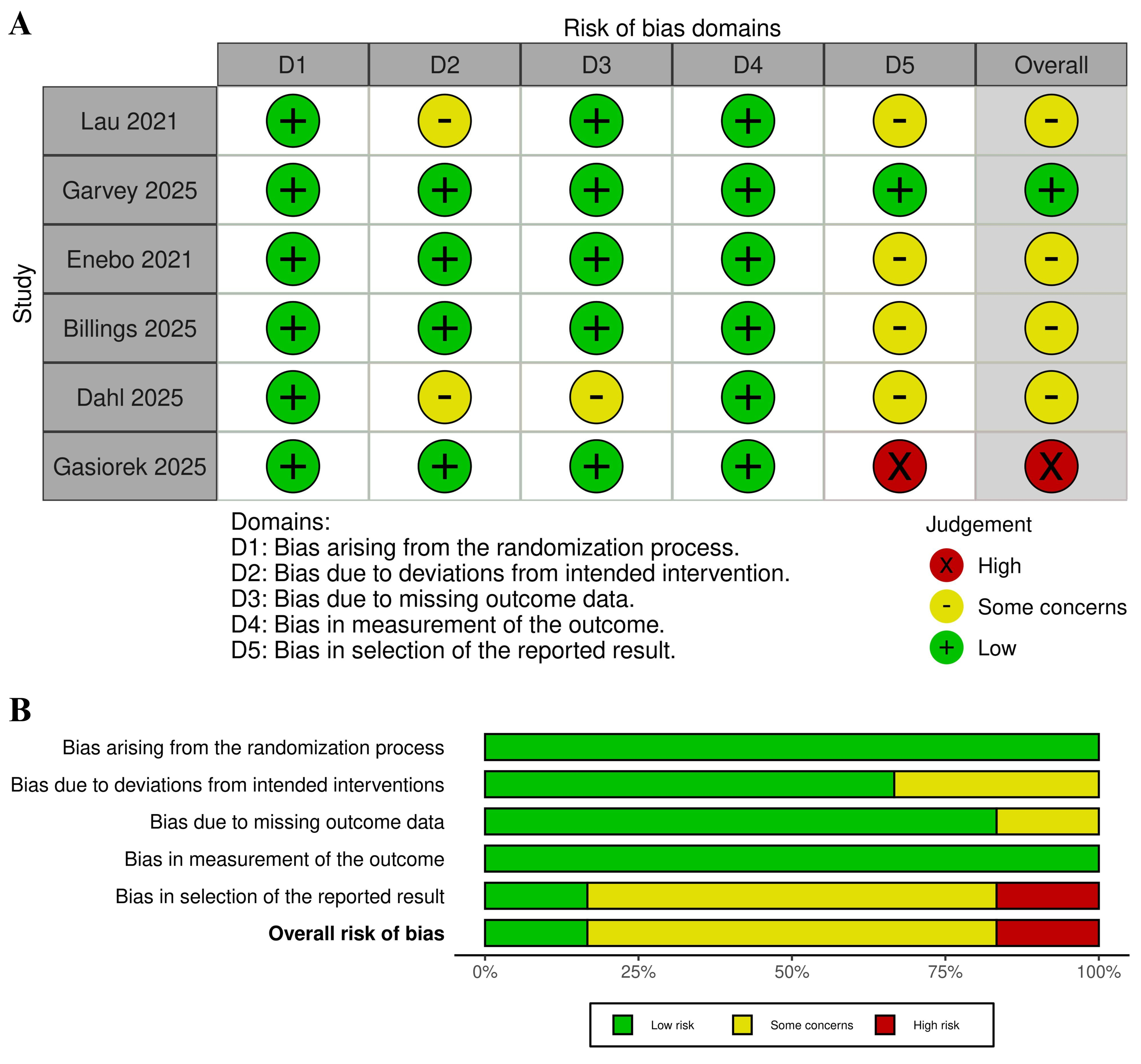
**

**Figure S2.** Comparison-adjusted funnel plots for the interventions for the percent change in body weight


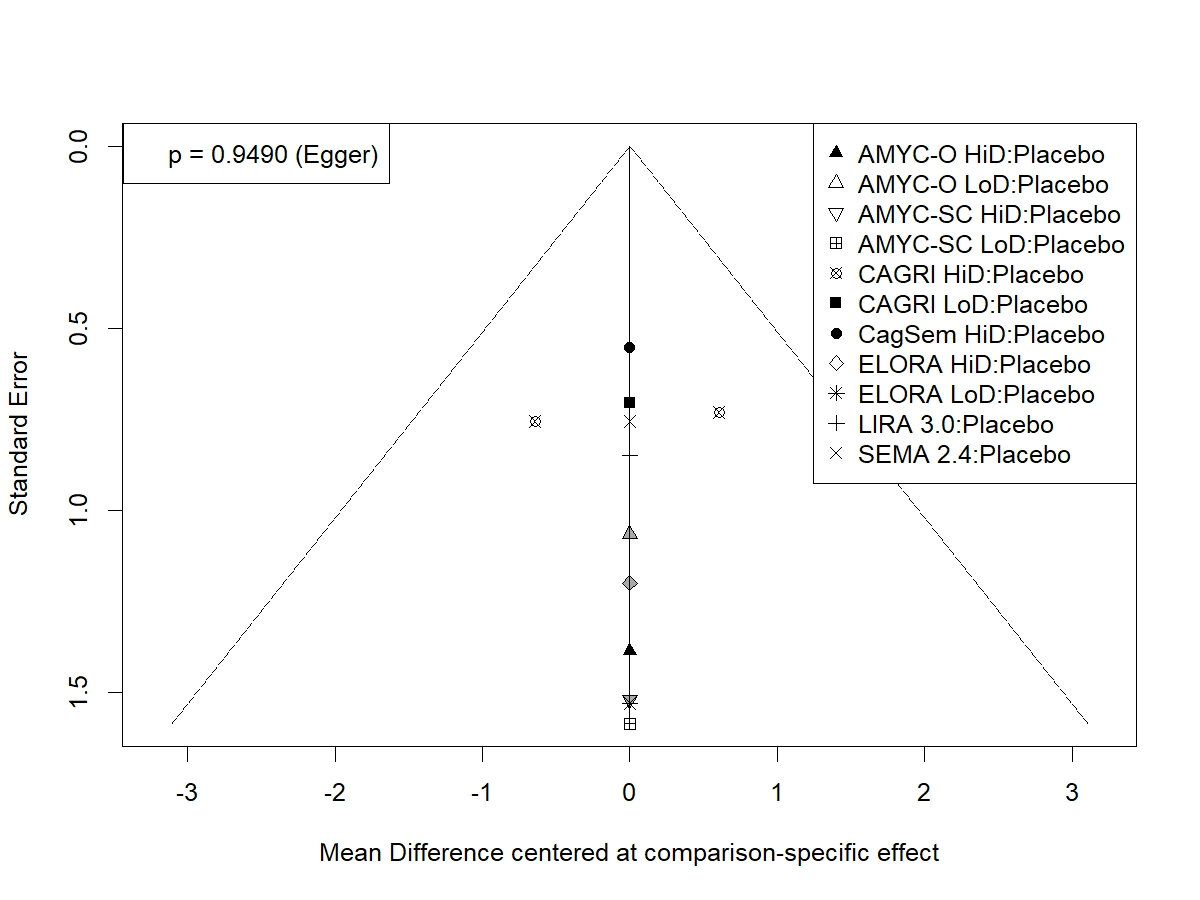


**Figure S3.** Netsplit estimates for separate direct and indirect evidence for the percent change in body weight


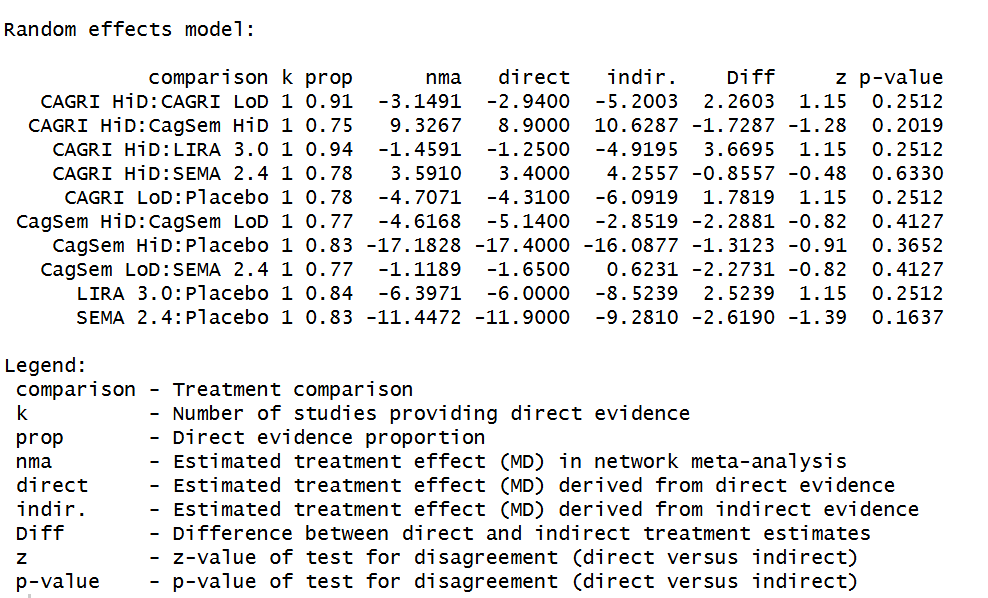


**Figure S4.** Network diagram (A), network meta-analysis forest plot (B), P score (C) for the absolute change in body weight, comparing various amylin-based therapies to placebo


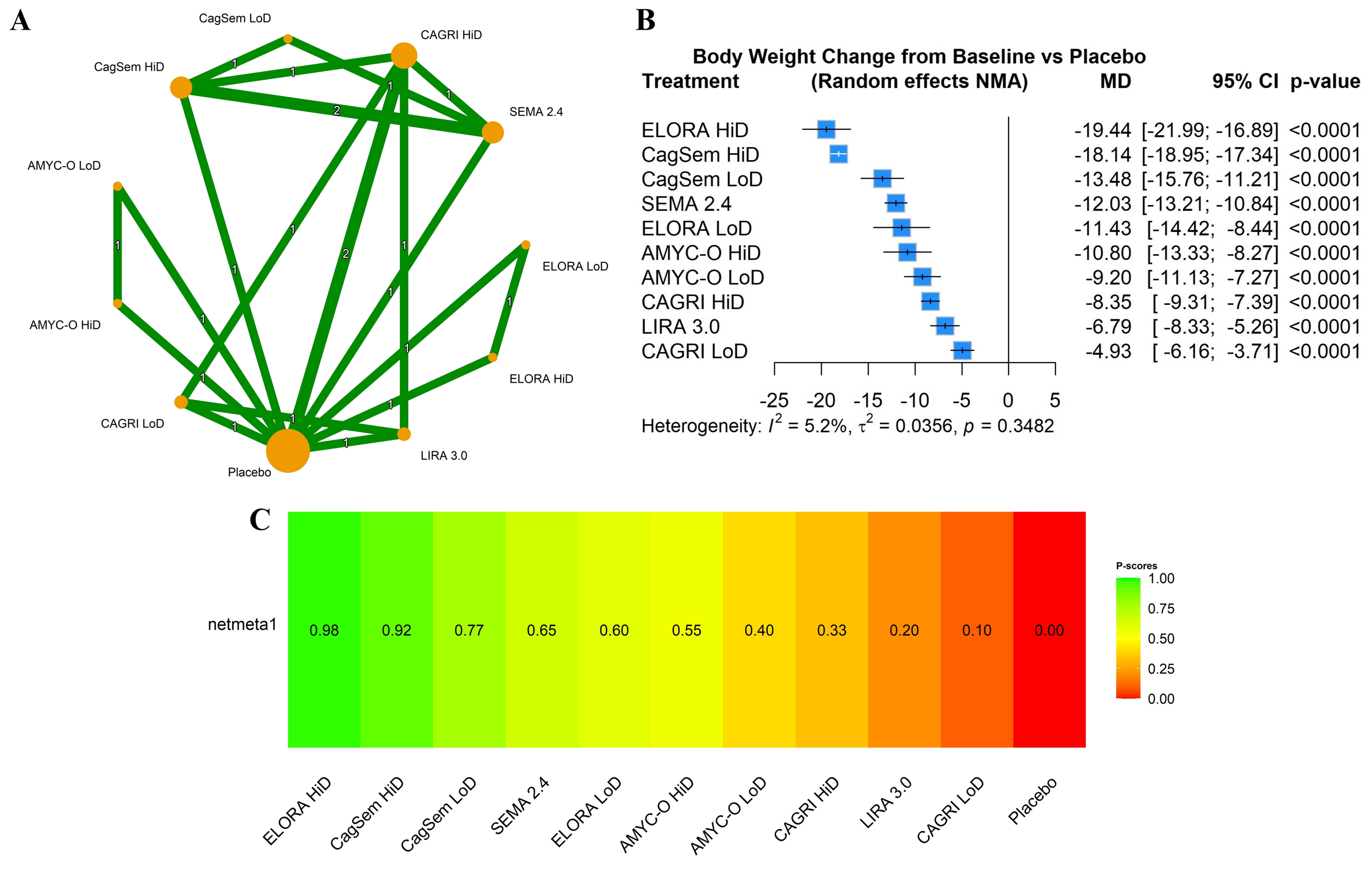


**Figure S5.** League table showing head-to-head comparisons among the interventions for absolute changes in body weight (kg)


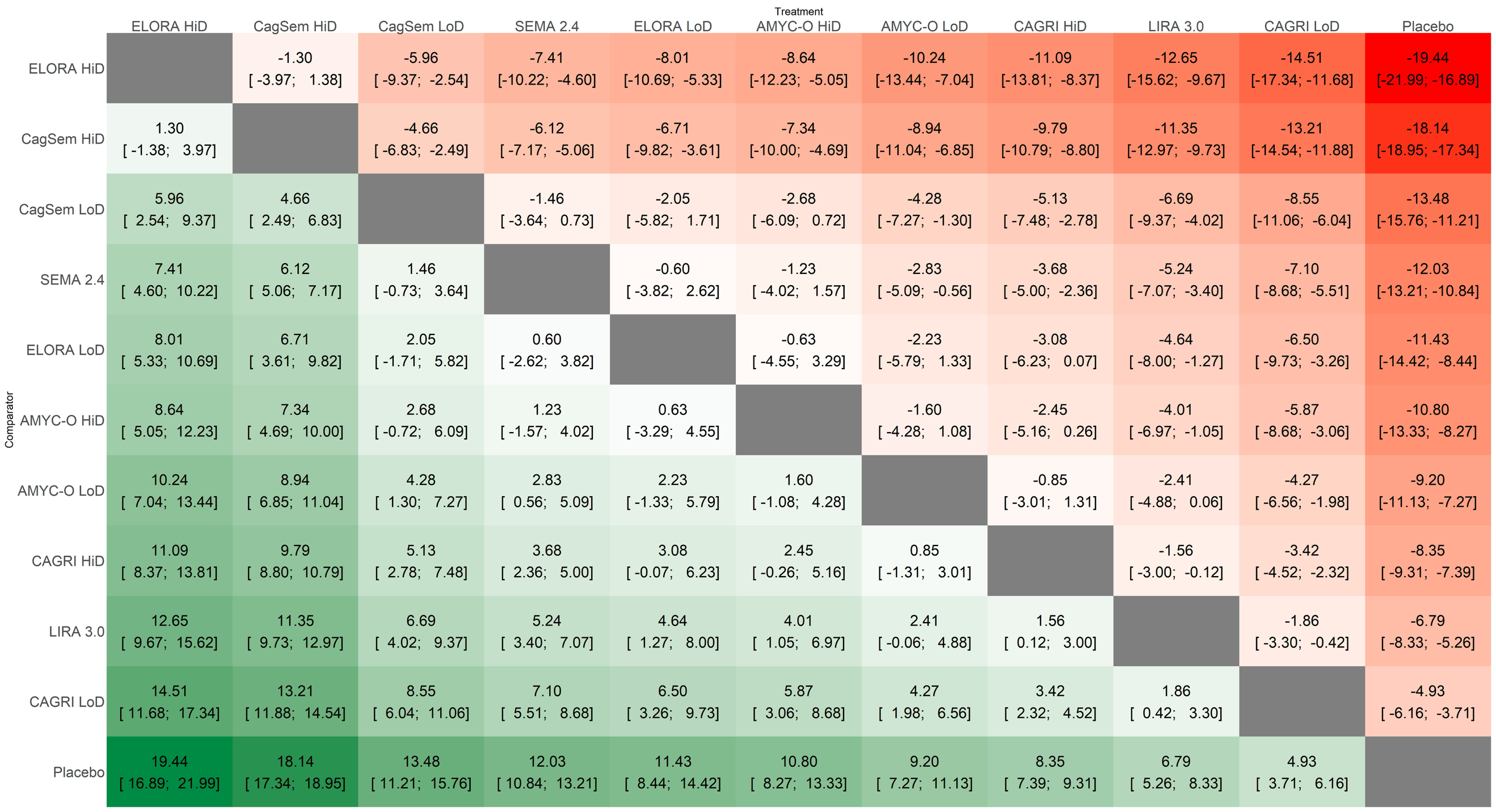


**Figure S6.** Netsplit estimates for separate direct and indirect evidence for absolute changes in body weight (kg)

**
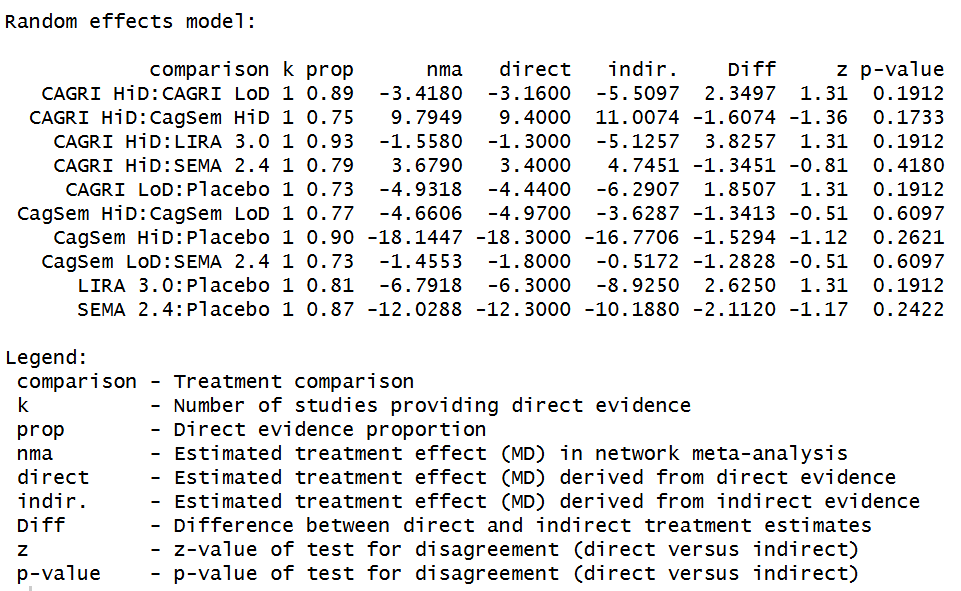
**

**Figure S7.** Network diagram (A), network meta-analysis forest plot (B), P score (C) for the change in body mass index, comparing various amylin-based therapies to placebo


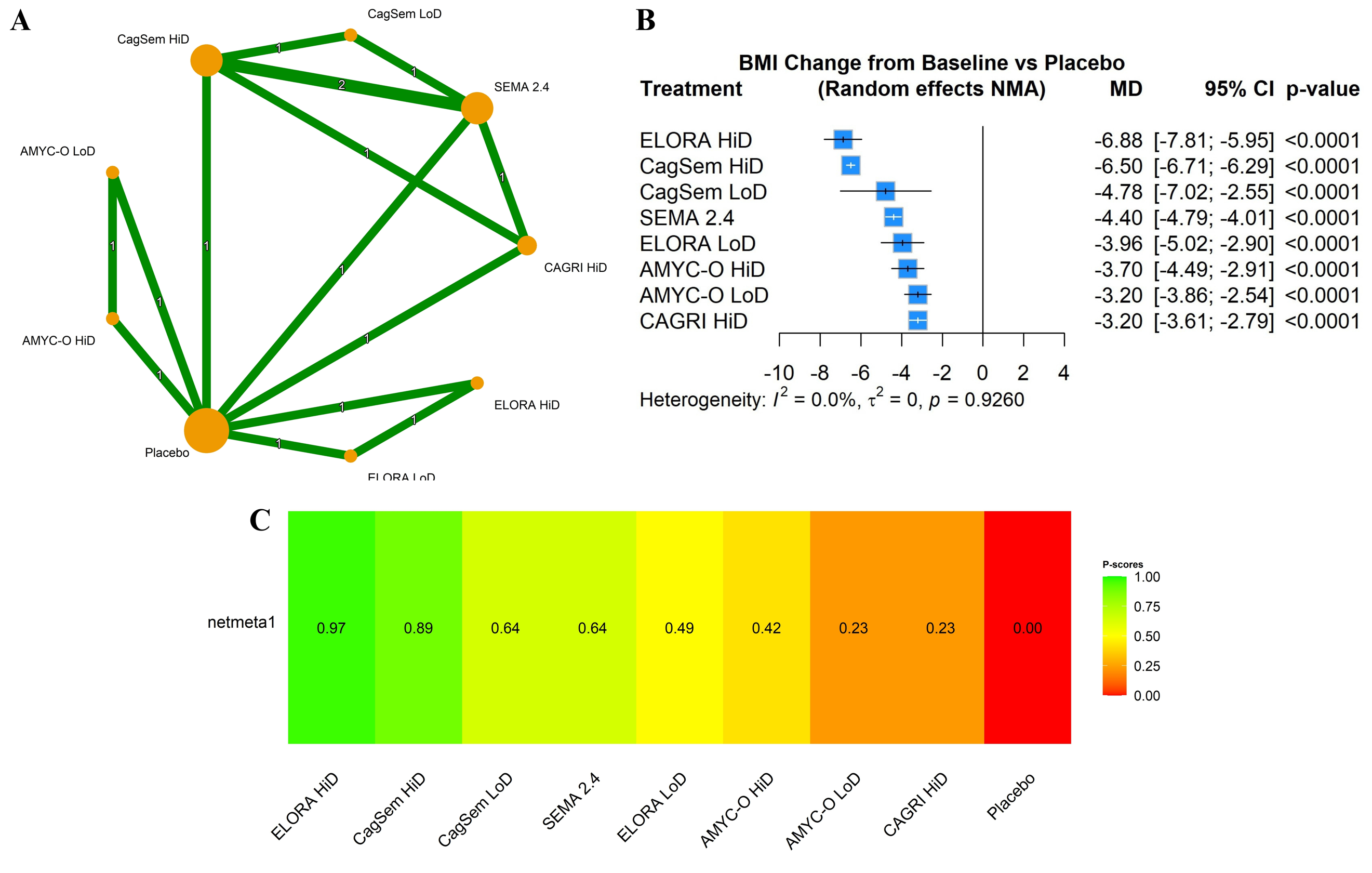


**Figure S8.** League table showing head-to-head comparisons among the interventions for changes in body mass index


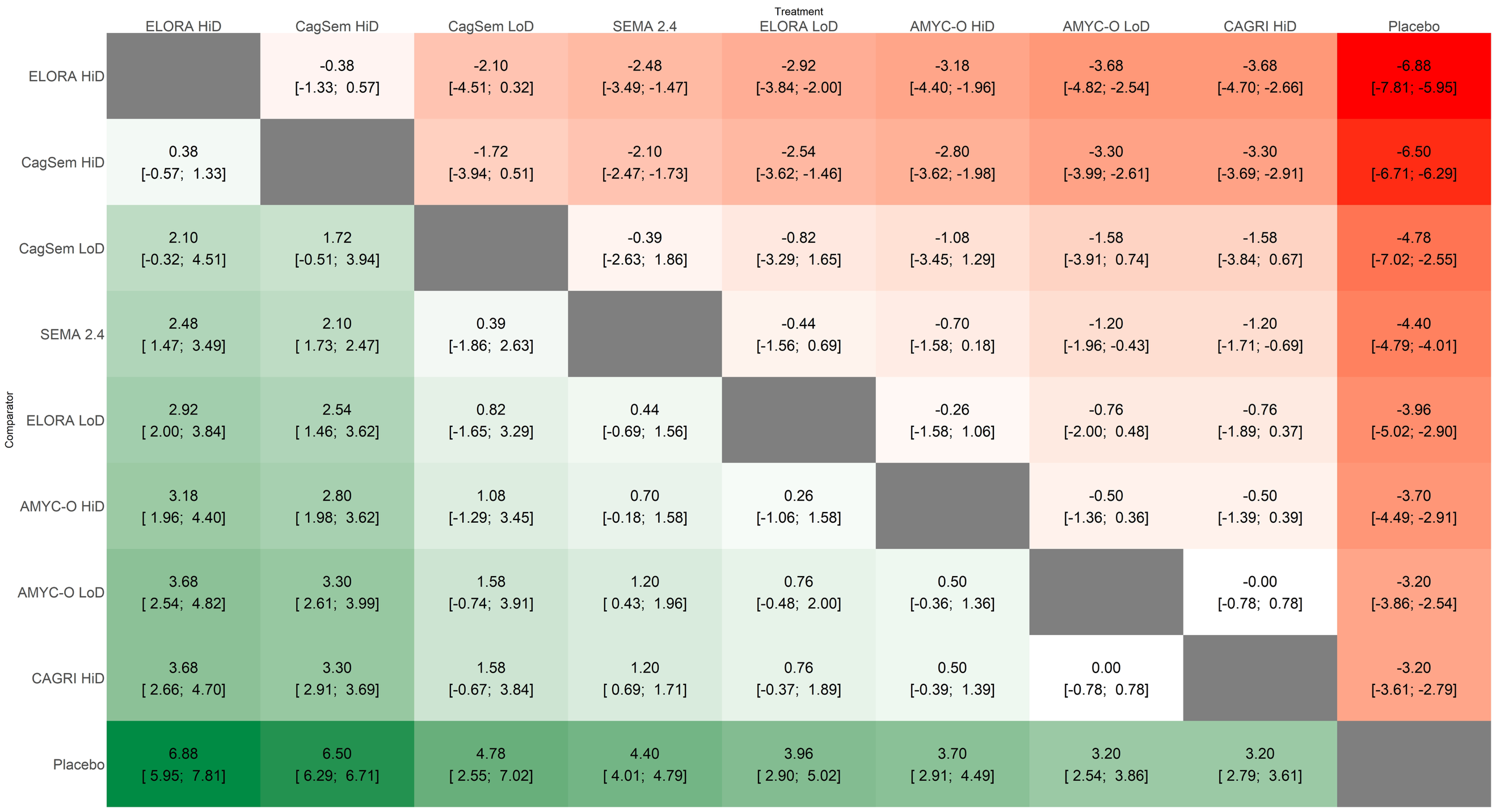


**Figure S9.** Netsplit estimates for separate direct and indirect evidence for changes in body mass index


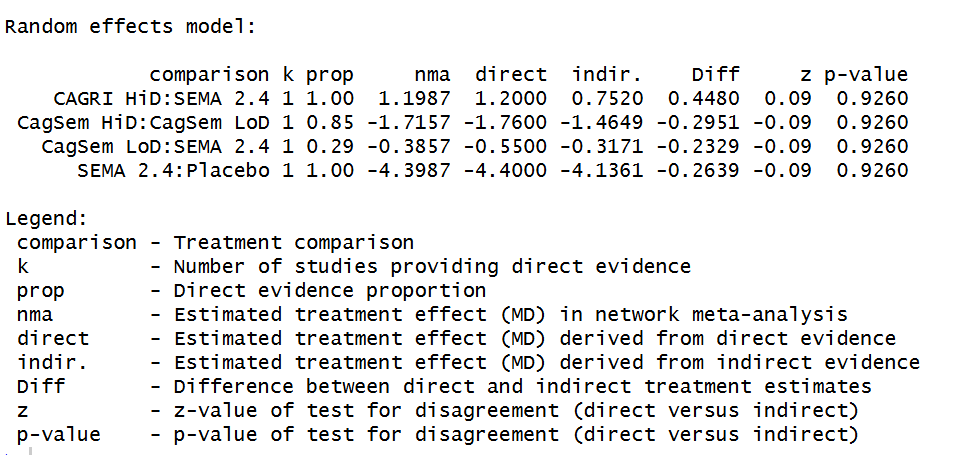


**Figure S10.** Network diagram (A), network meta-analysis forest plot (B), P score (C) for the change in waist circumference, comparing various amylin-based therapies to placebo


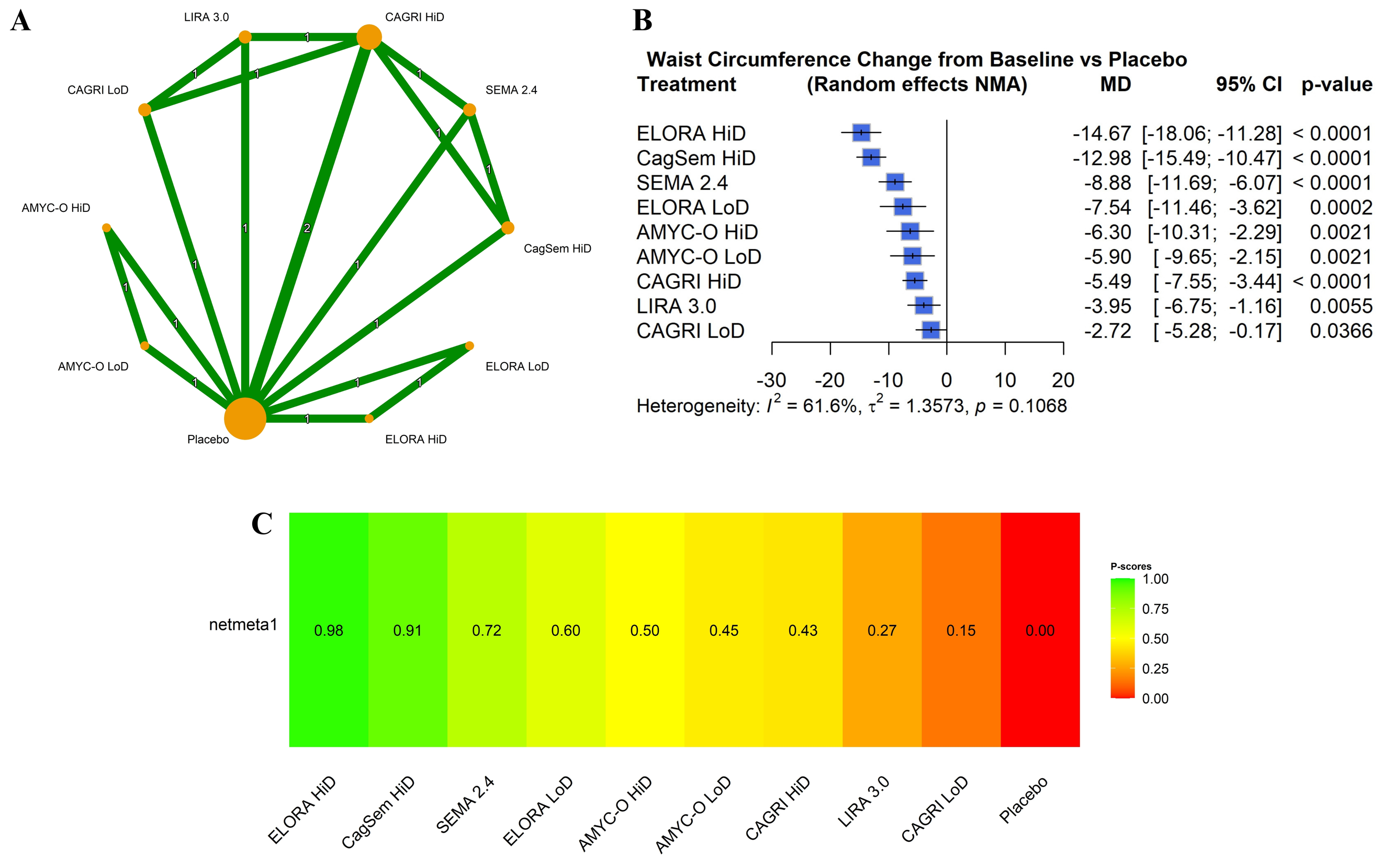


**Figure S11.** League table showing head-to-head comparisons among the interventions for changes in waist circumference


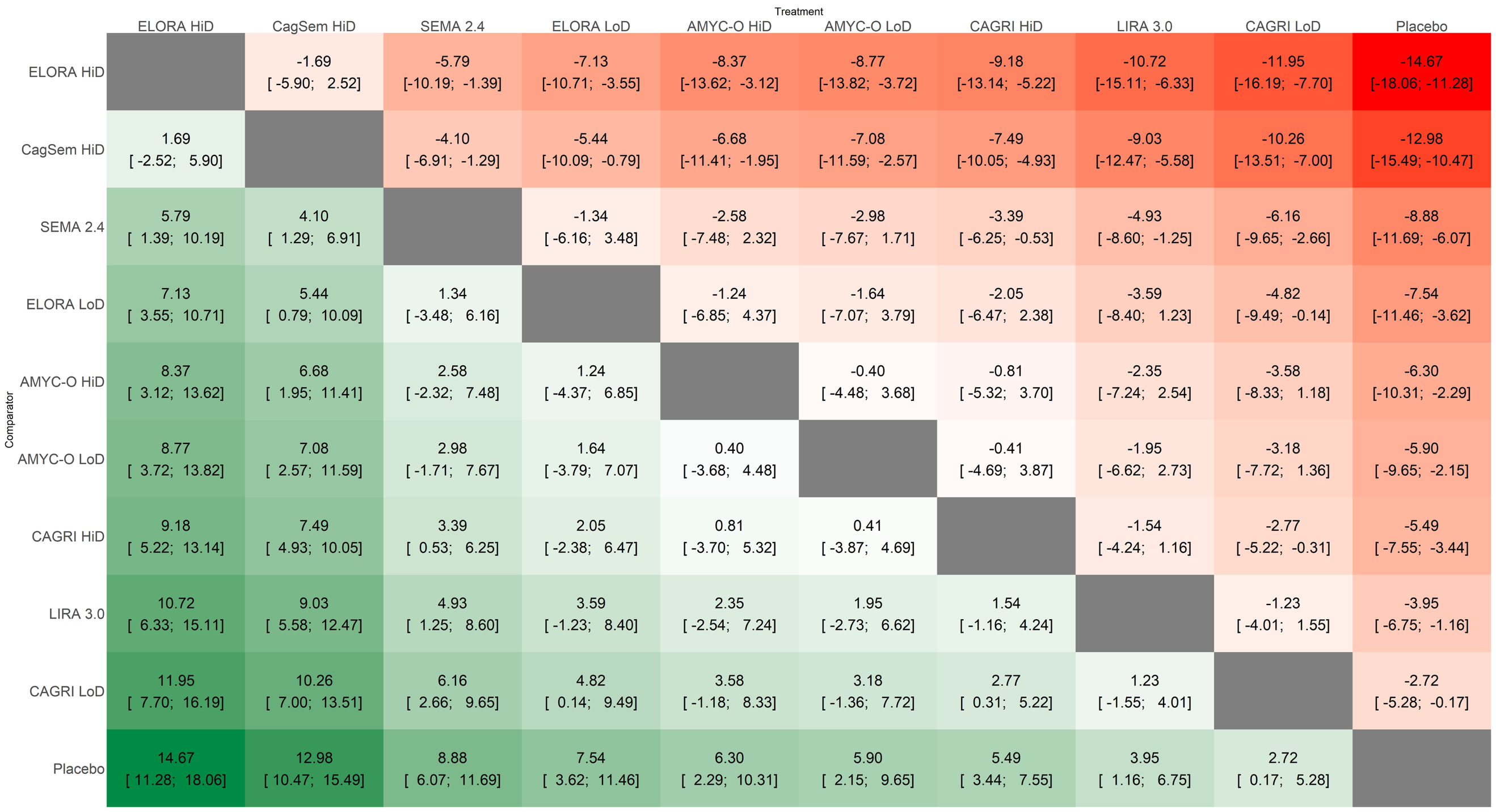


**Figure S12.** Netsplit estimates for separate direct and indirect evidence for changes in waist circumference


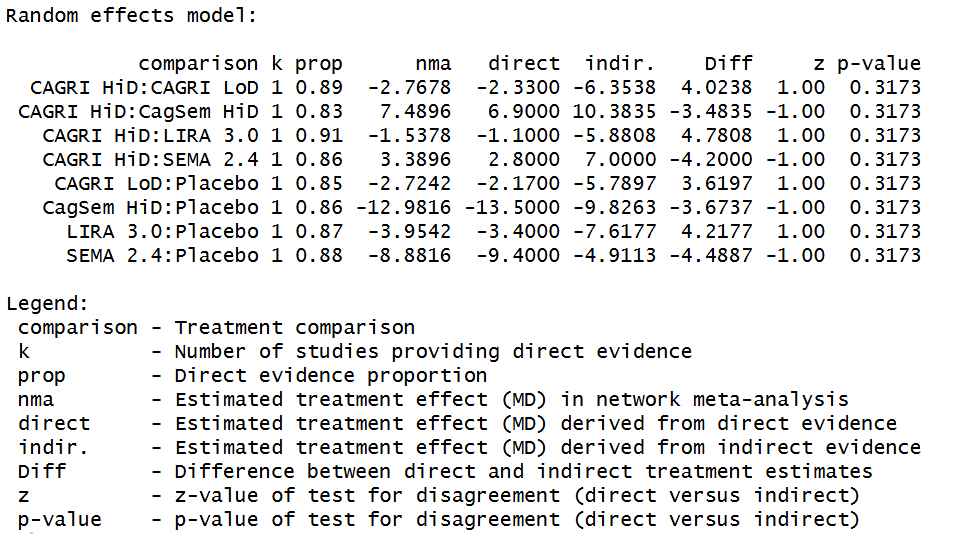


**Figure S13.** Network diagram (A), network meta-analysis forest plot (B), P score (C) for the proportions of study subjects who lost ≥5% of their baseline body weight, comparing various amylin-based therapies to placebo

[Three studies (8 treatments, 15 pairwise comparisons), no heterogeneity and inconsistency (tau^2^ = 0; tau = 0; I^2^ = 0%), non-significant Q]


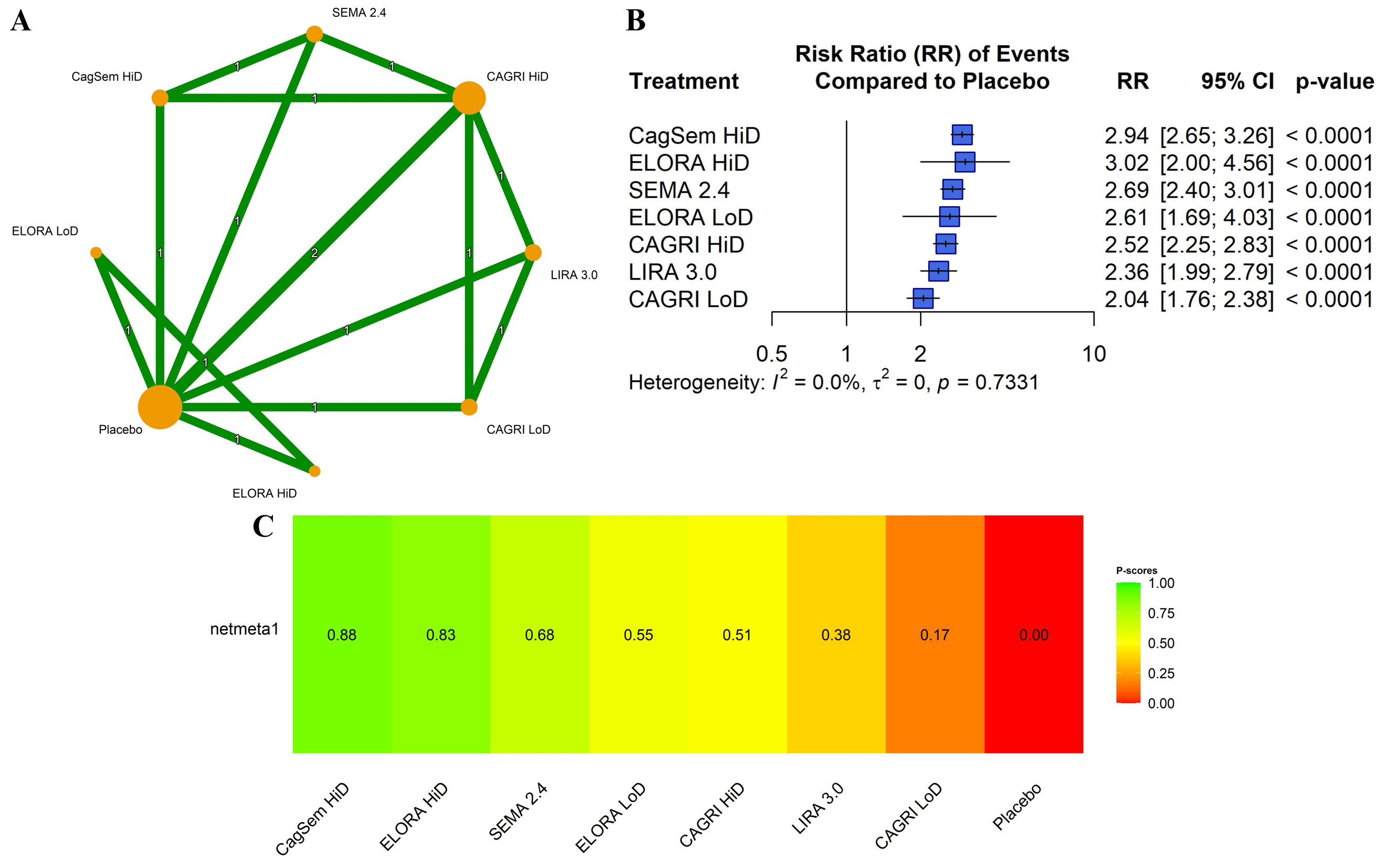


**Figure S14.** League table showing head-to-head comparisons among the interventions for the proportions of study subjects who lost ≥5% of their baseline body weight


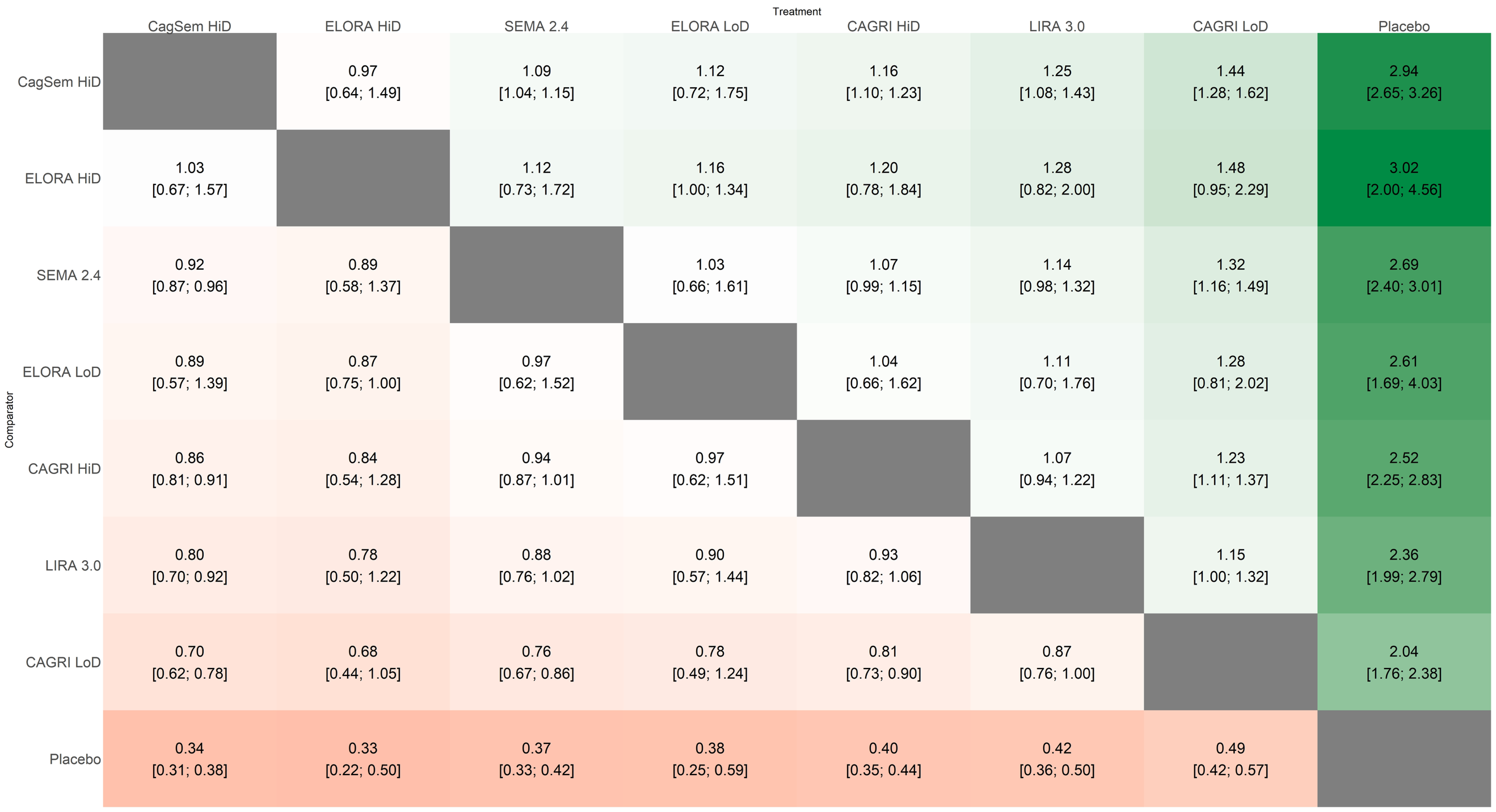


**Figure S15.** Netsplit estimates for separate direct and indirect evidence for the proportions of study subjects who lost ≥5% of their baseline body weight


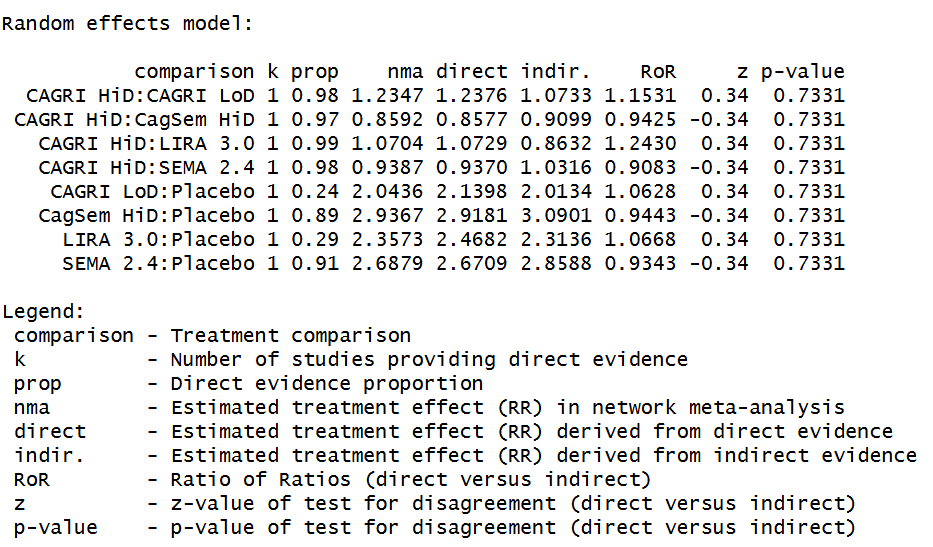


**Figure S16.** Network diagram (A), network meta-analysis forest plot (B), P score (C) for the proportions of study subjects who lost ≥10% of their baseline body weight, comparing various amylin-based therapies to placebo

[Three studies (8 treatments, 15 pairwise comparisons), no heterogeneity and inconsistency (tau^2^ = 0; tau = 0; I^2^ = 0%), non-significant Q]


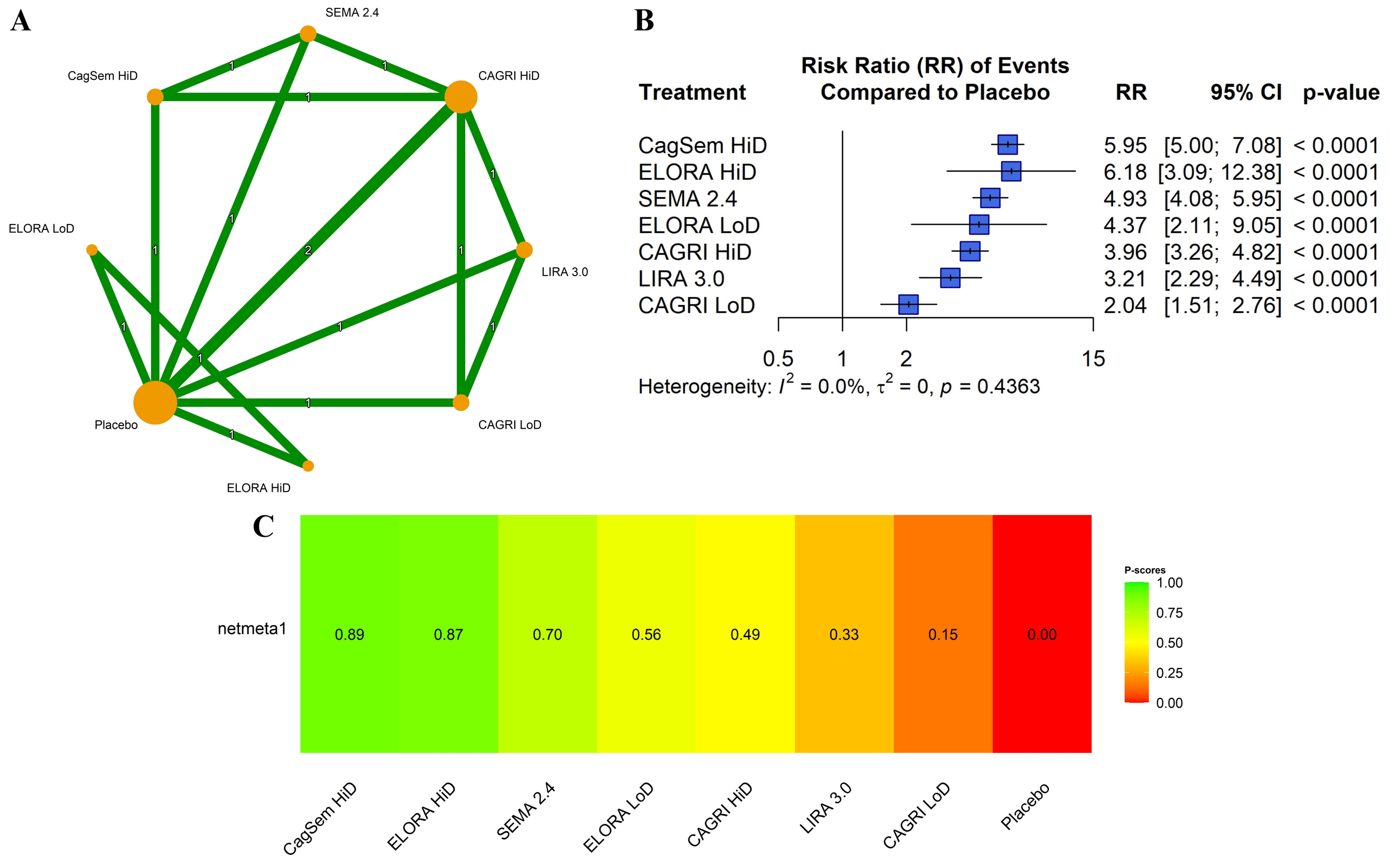


**Figure S17.** League table showing head-to-head comparisons among the interventions for the proportions of study subjects who lost ≥10% of their baseline body weight


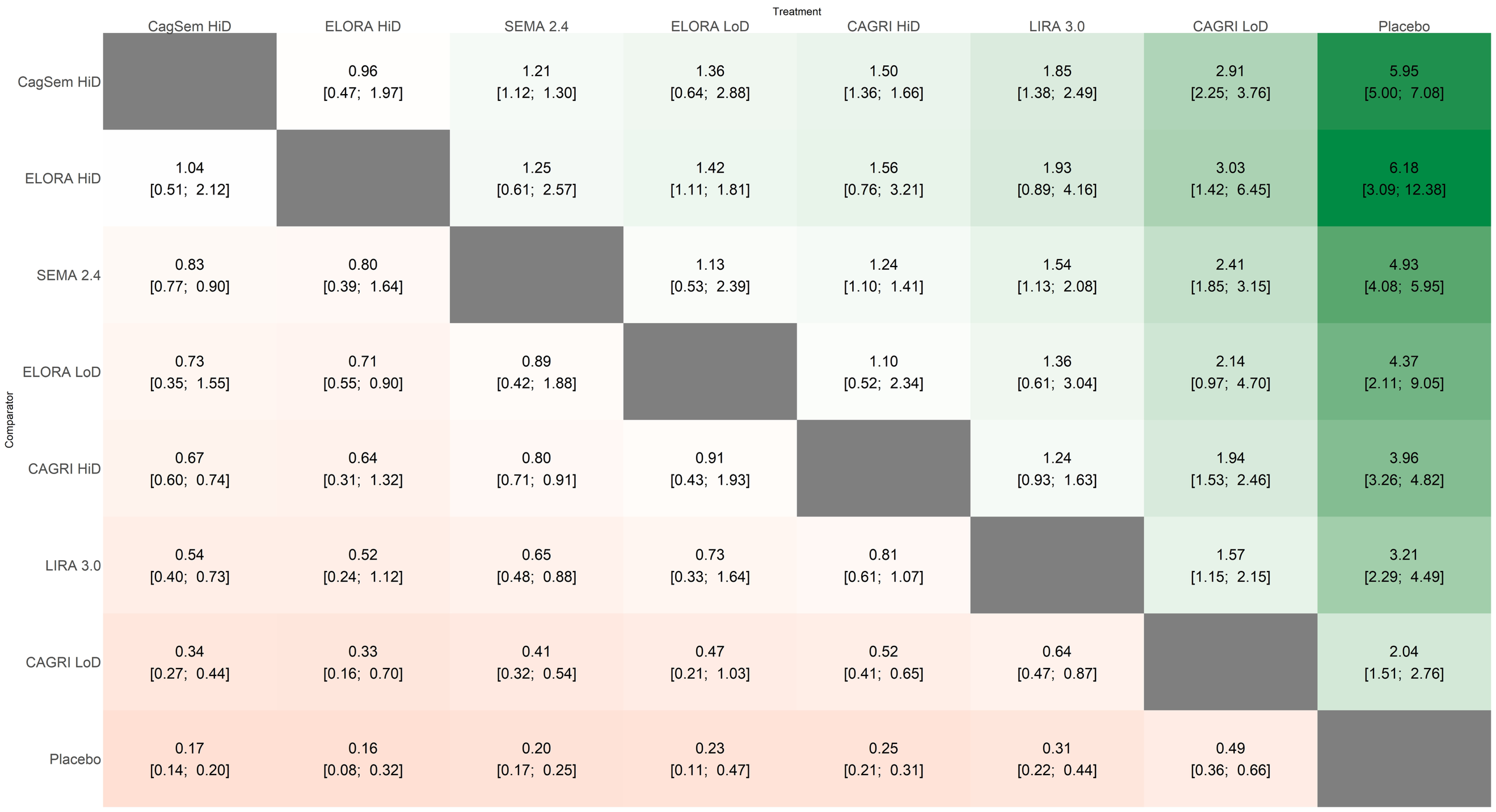


**Figure S18.** Netsplit estimates for separate direct and indirect evidence for the proportions of study subjects who lost ≥10% of their baseline body weight


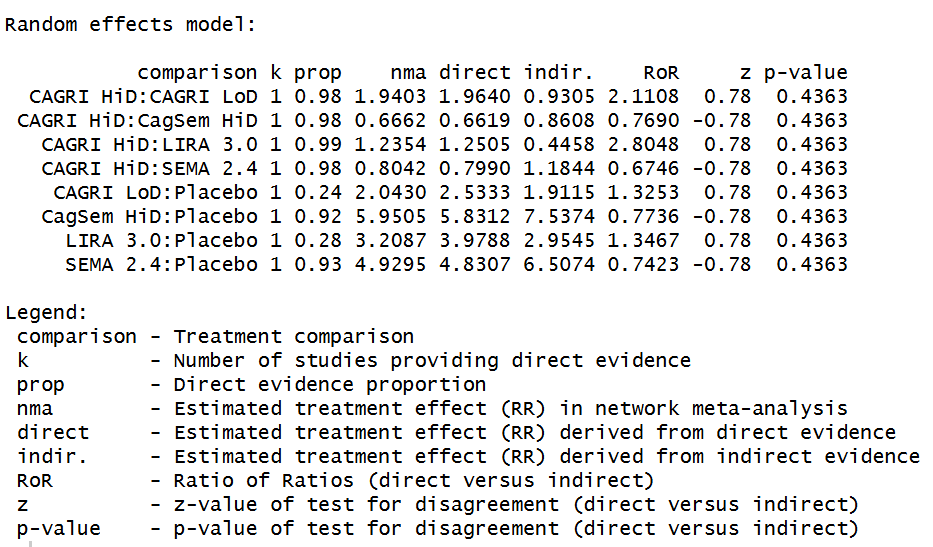


**Figure S19.** Network diagram (A), network meta-analysis forest plot (B), P score (C) for the proportions of study subjects who lost ≥15% of their baseline body weight, comparing various amylin-based therapies to placebo

[Three studies (8 treatments, 15 pairwise comparisons), no heterogeneity and inconsistency (tau^2^ = 0; tau = 0; I^2^ = 0%), non-significant Q]


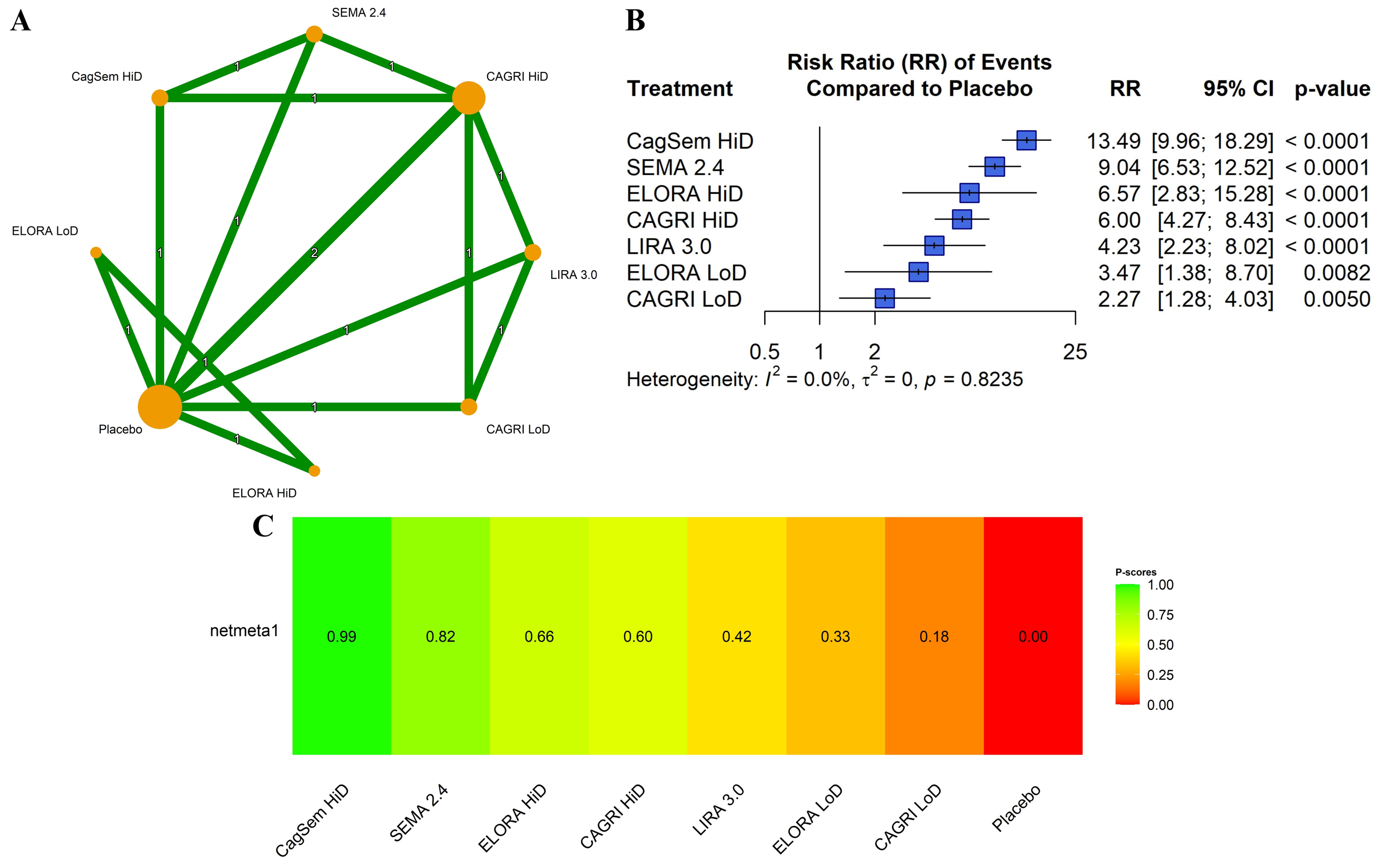


**Figure S20.** League table showing head-to-head comparisons among the interventions for the proportions of study subjects who lost ≥15% of their baseline body weight


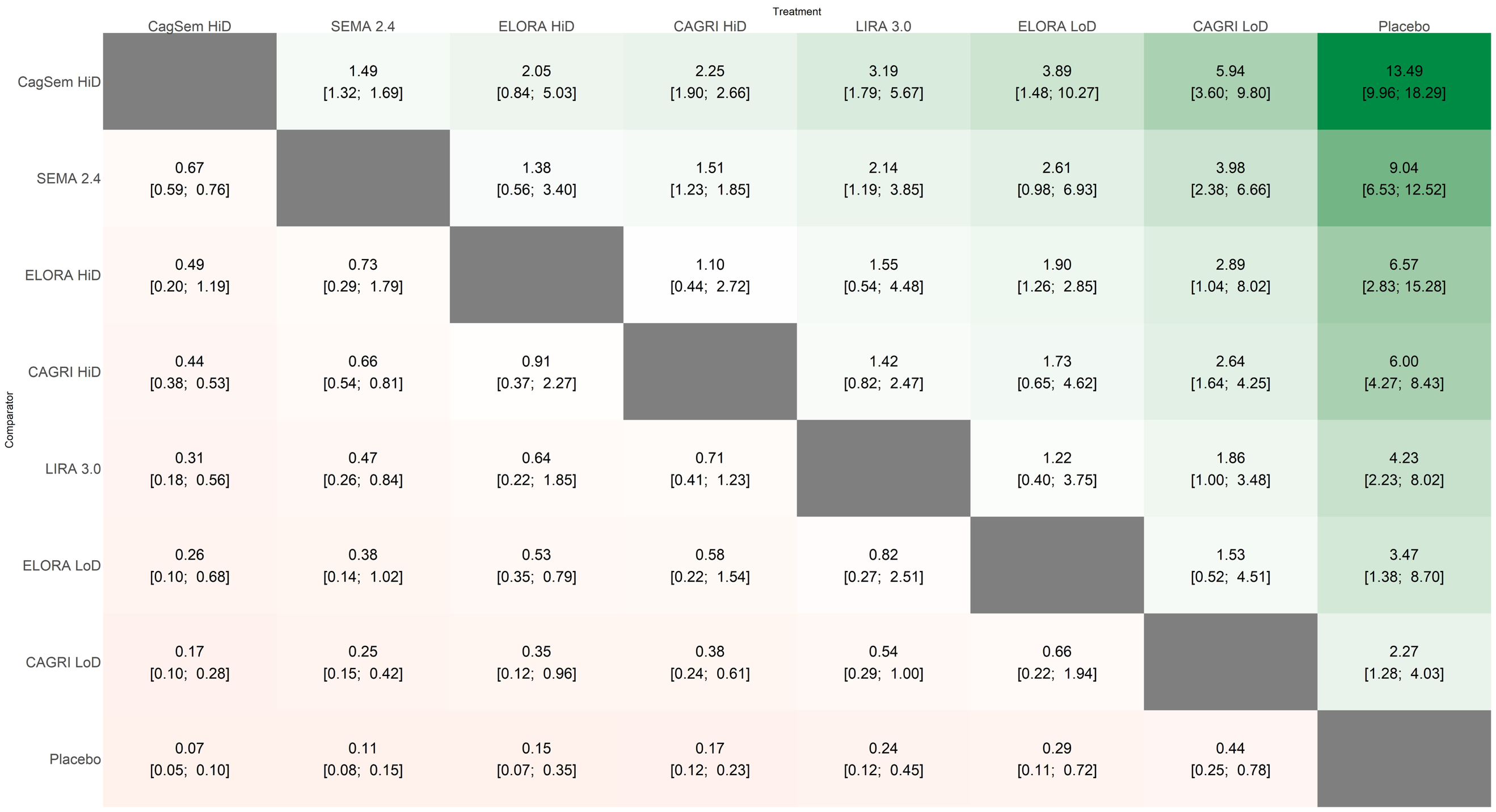


**Figure S21.** Netsplit estimates for separate direct and indirect evidence for the proportions of study subjects who lost ≥15% of their baseline body weight


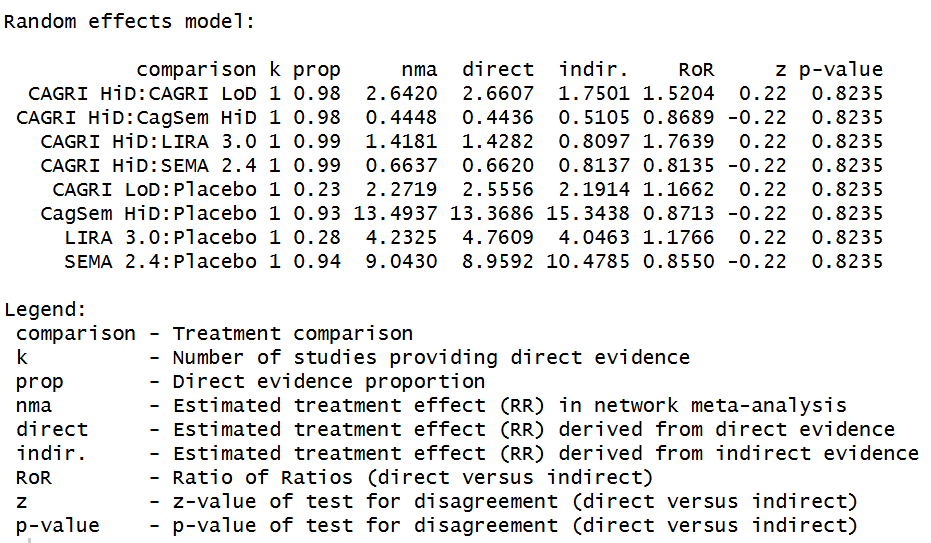


**Figure S22.** Network diagram (A), network meta-analysis forest plot (B), P score (C) for the proportions of study subjects who experienced gastrointestinal adverse events, comparing various amylin-based therapies to placebo

[Six studies (13 treatments, 24 pairwise comparisons), mild heterogeneity and inconsistency (tau^2^ = 0.0044; tau = 0.0661; I^2^ = 27.4%), non-significant Q]


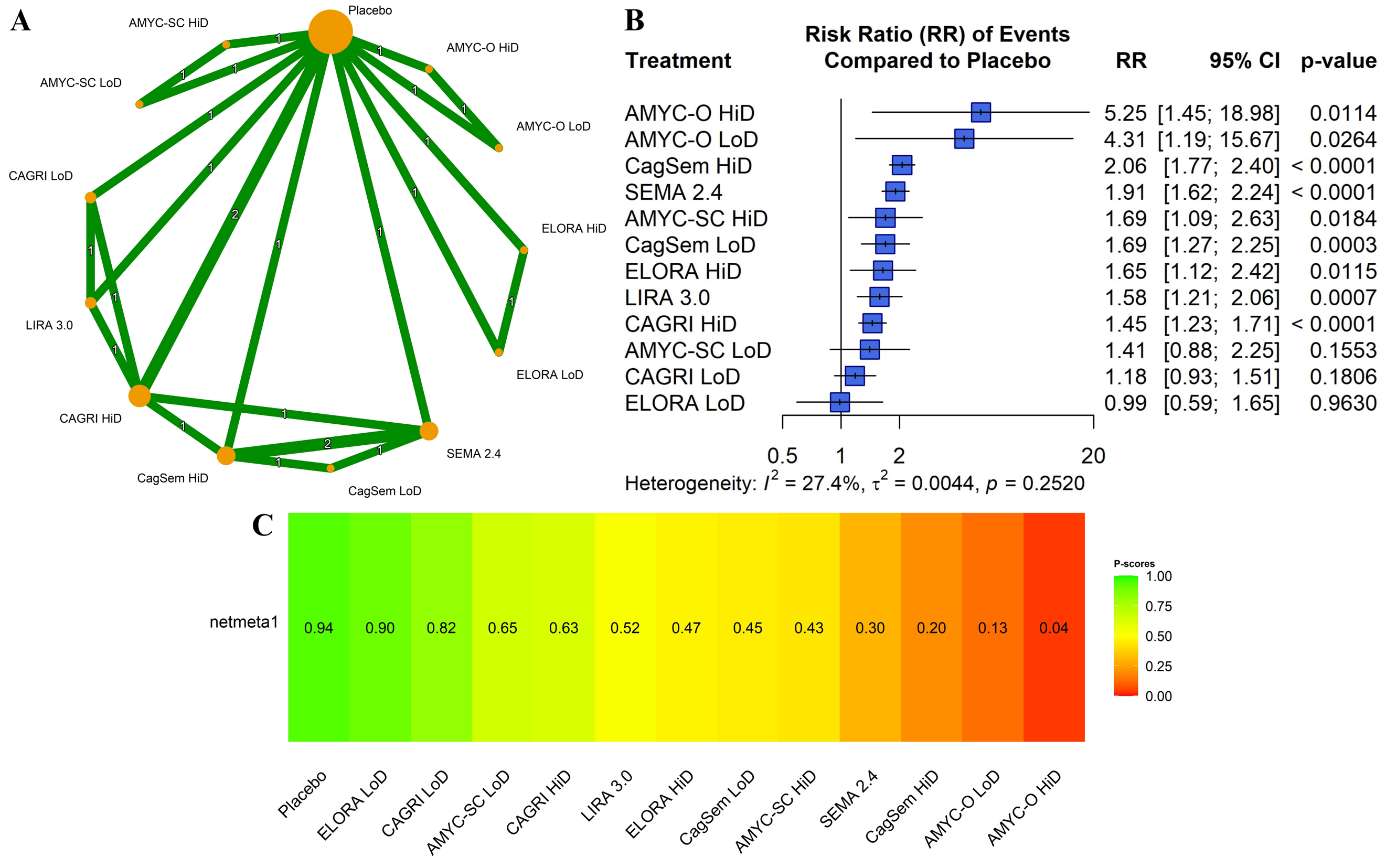


**Figure S23.** League table showing head-to-head comparisons among the interventions for the proportions of study subjects who experienced gastrointestinal adverse events, comparing various amylin-based therapies to placebo


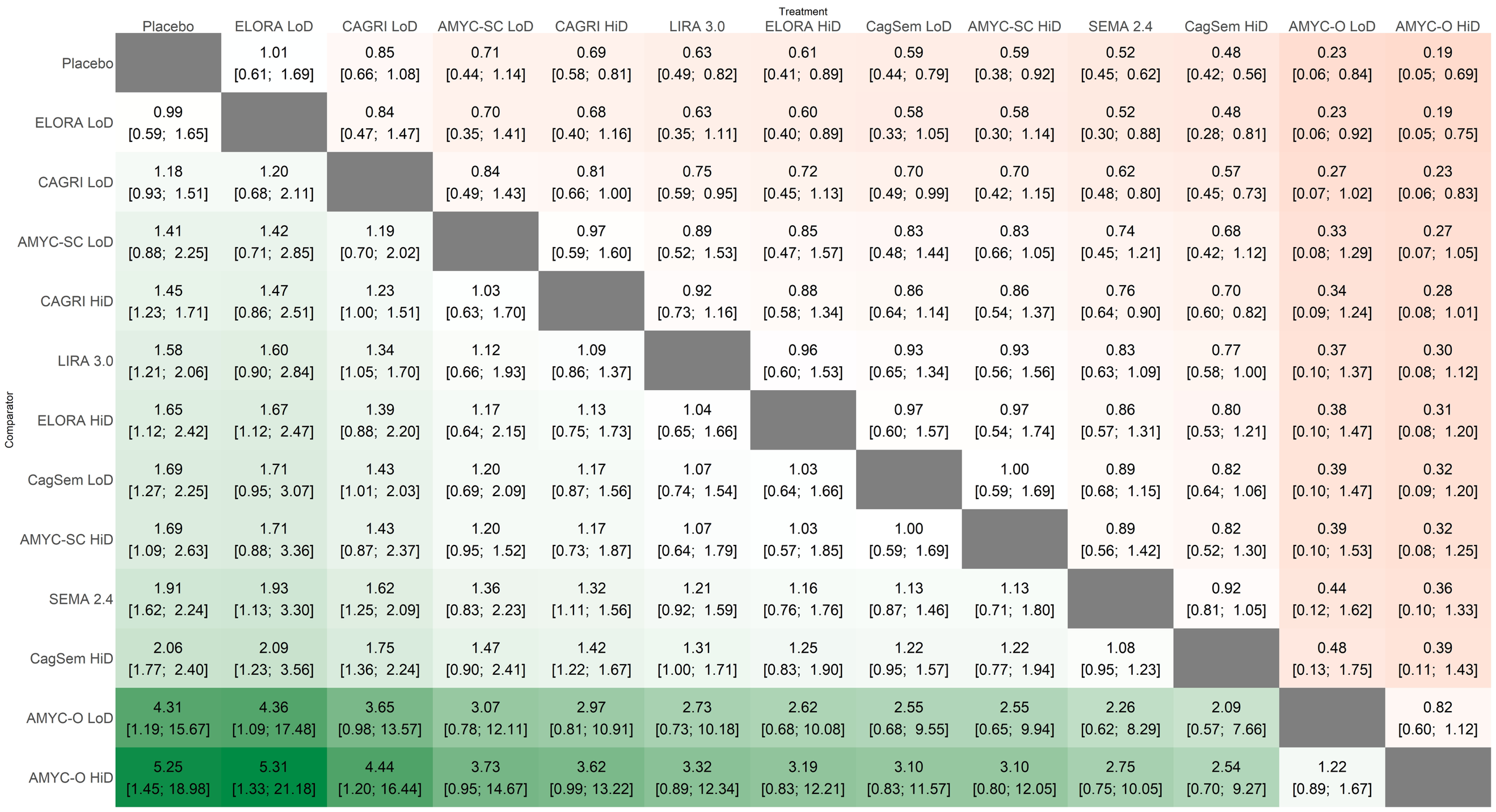


**Figure S24.** Netsplit estimates for separate direct and indirect evidence for the proportions of study subjects who experienced gastrointestinal adverse events, comparing various amylin-based therapies to placebo


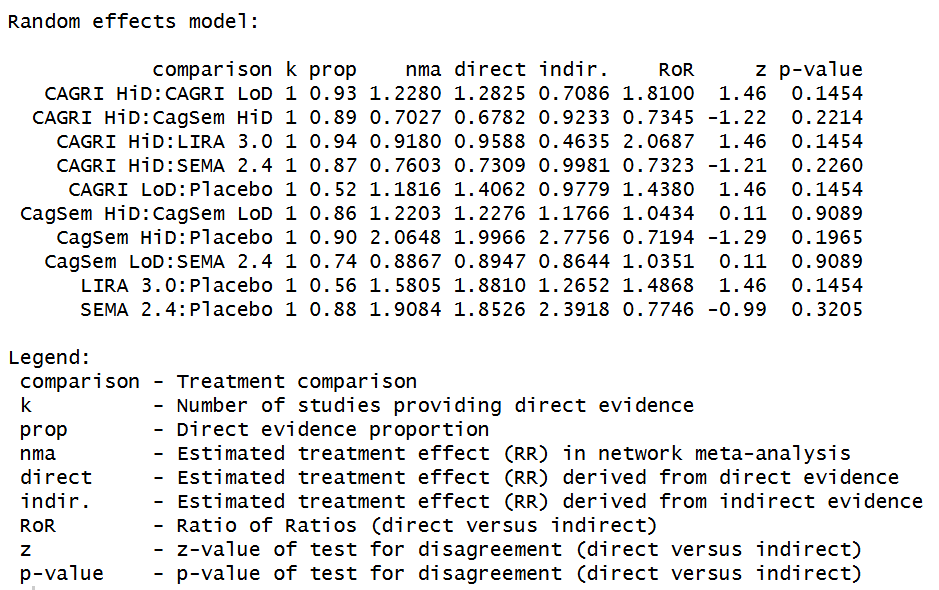


**Figure S25.** Network diagram (A), network meta-analysis forest plot (B), P score (C) for the proportions of study subjects who experienced nausea, comparing various amylin-based therapies to placebo

[Six studies (13 treatments, 24 pairwise comparisons), no heterogeneity and inconsistency (tau^2^ = 0; tau = 0; I^2^ = 0%), non-significant Q]


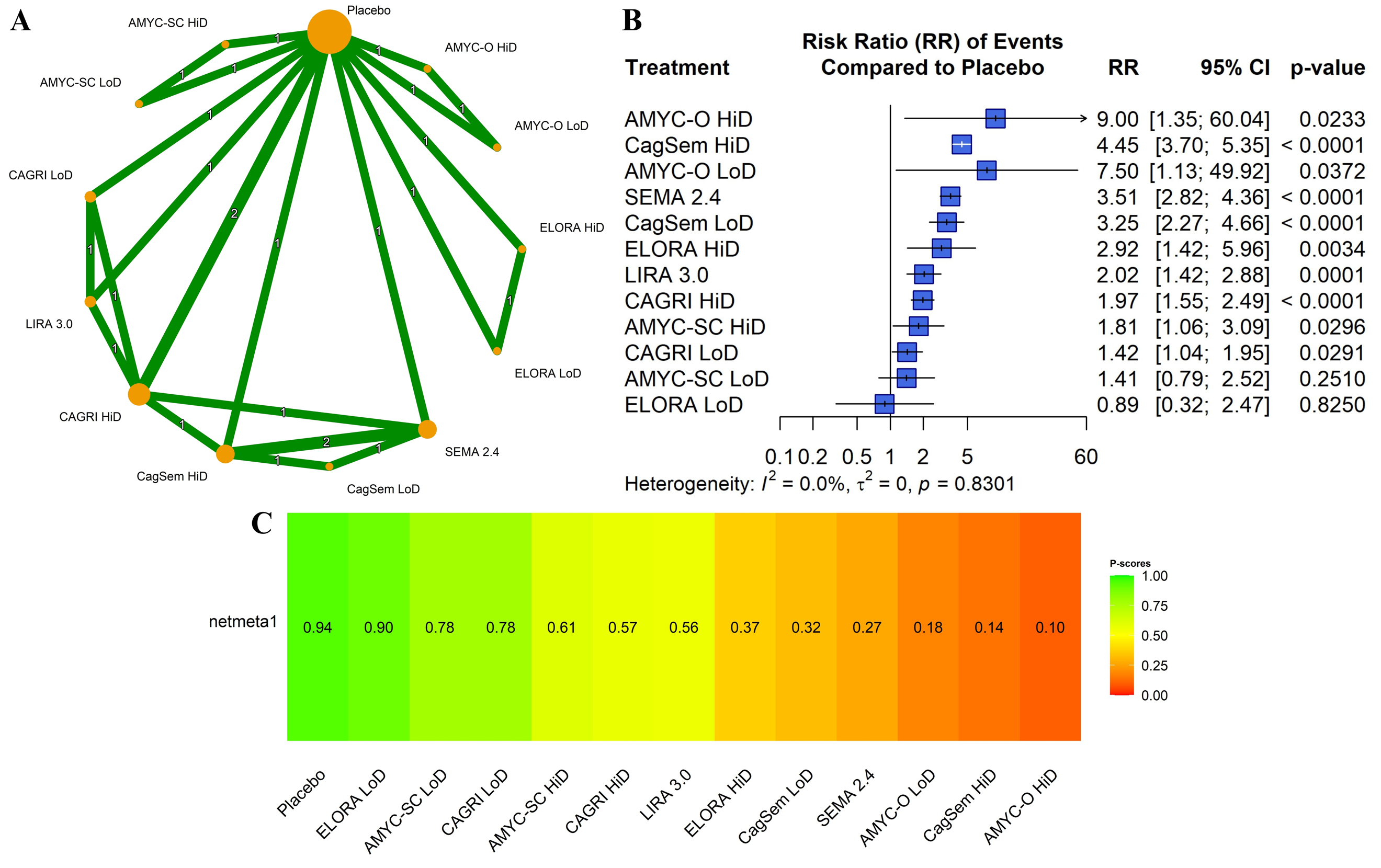


**Figure S26.** League table showing head-to-head comparisons among the interventions for the proportions of study subjects who experienced nausea


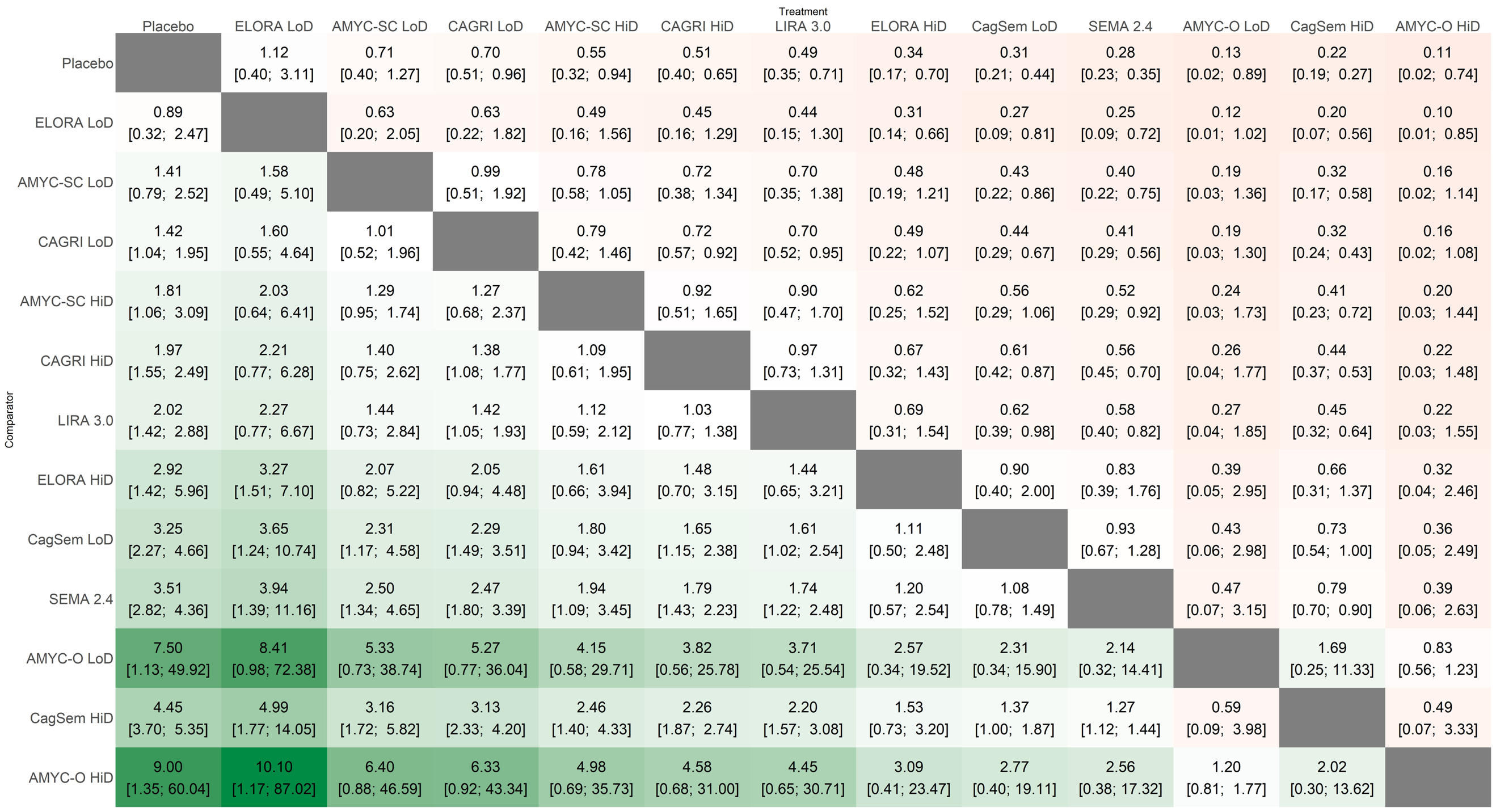


**Figure S27.** Netsplit estimates for separate direct and indirect evidence for the proportions of study subjects who experienced nausea

**
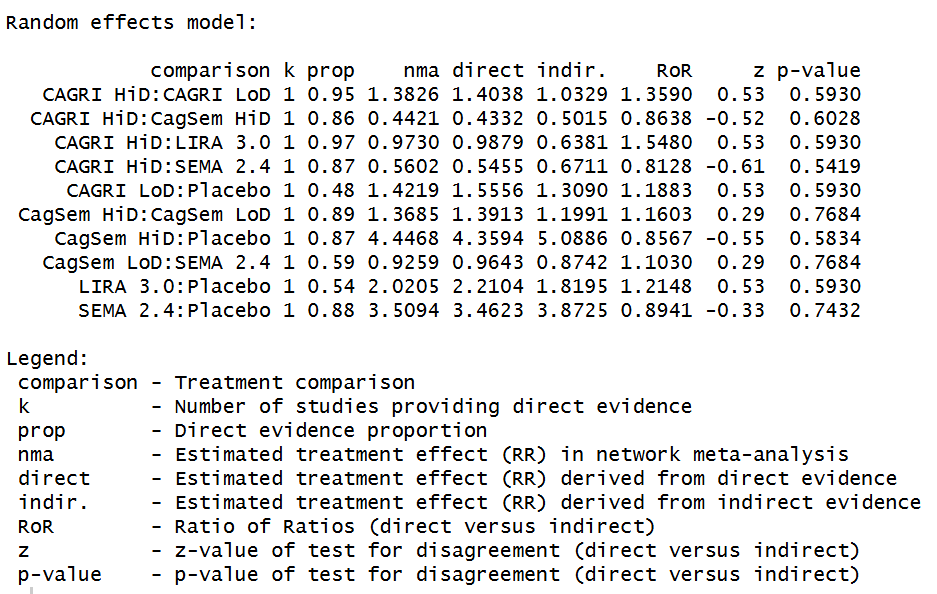
**

**Figure S28.** Network diagram (A), network meta-analysis forest plot (B), P score (C) for the proportions of study subjects who experienced vomiting, comparing various amylin-based therapies to placebo

[Six studies (13 treatments, 24 pairwise comparisons), moderate heterogeneity and inconsistency (tau^2^ = 0.4177; tau = 0.6463; I^2^ = 68.3%), Total Q = 6.31 (p = 0.0426]


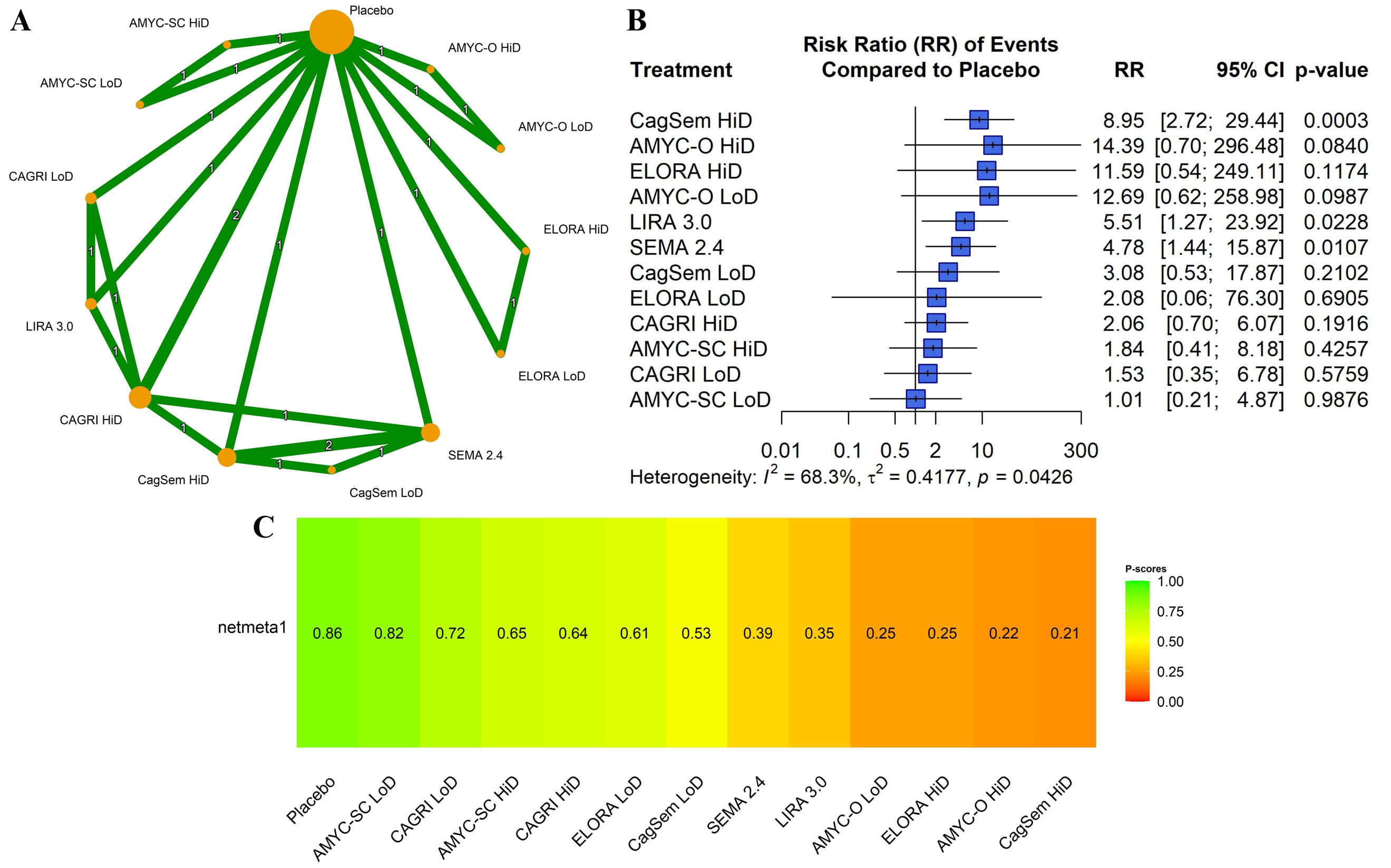


**Figure S29.** League table showing head-to-head comparisons among the interventions for the proportions of study subjects who experienced vomiting


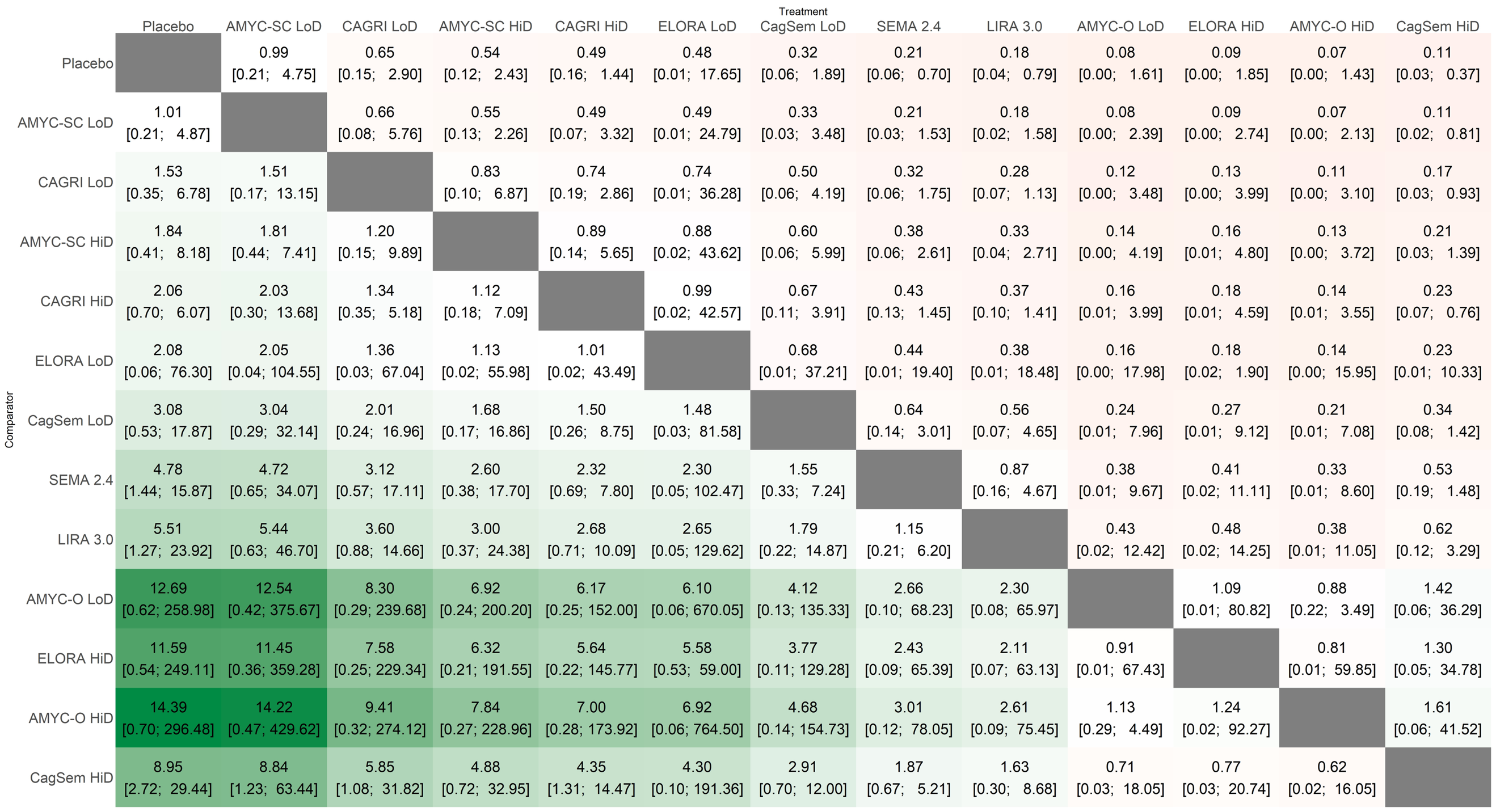


**Figure S30.** Netsplit estimates for separate direct and indirect evidence for the proportions of study subjects who experienced vomiting

**
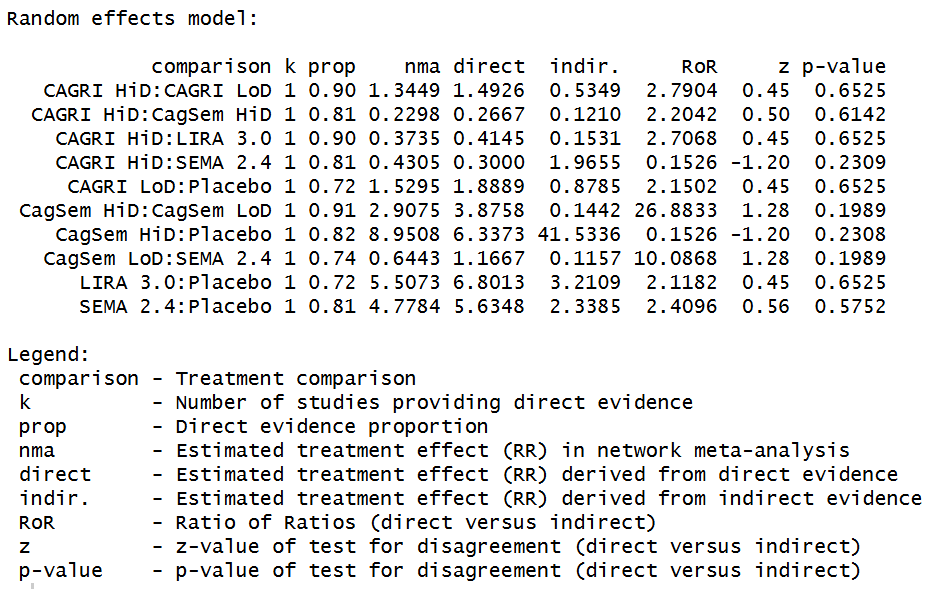
**

**Figure S31.** Network diagram (A), network meta-analysis forest plot (B), P score (C) for the proportions of study subjects who experienced diarrhea, comparing various amylin-based therapies to placebo

[Six studies (13 treatments, 24 pairwise comparisons), mild heterogeneity and inconsistency (tau^2^ = 0.1067; tau = 0.3266; I^2^ = 45.8%), non-significant Q]


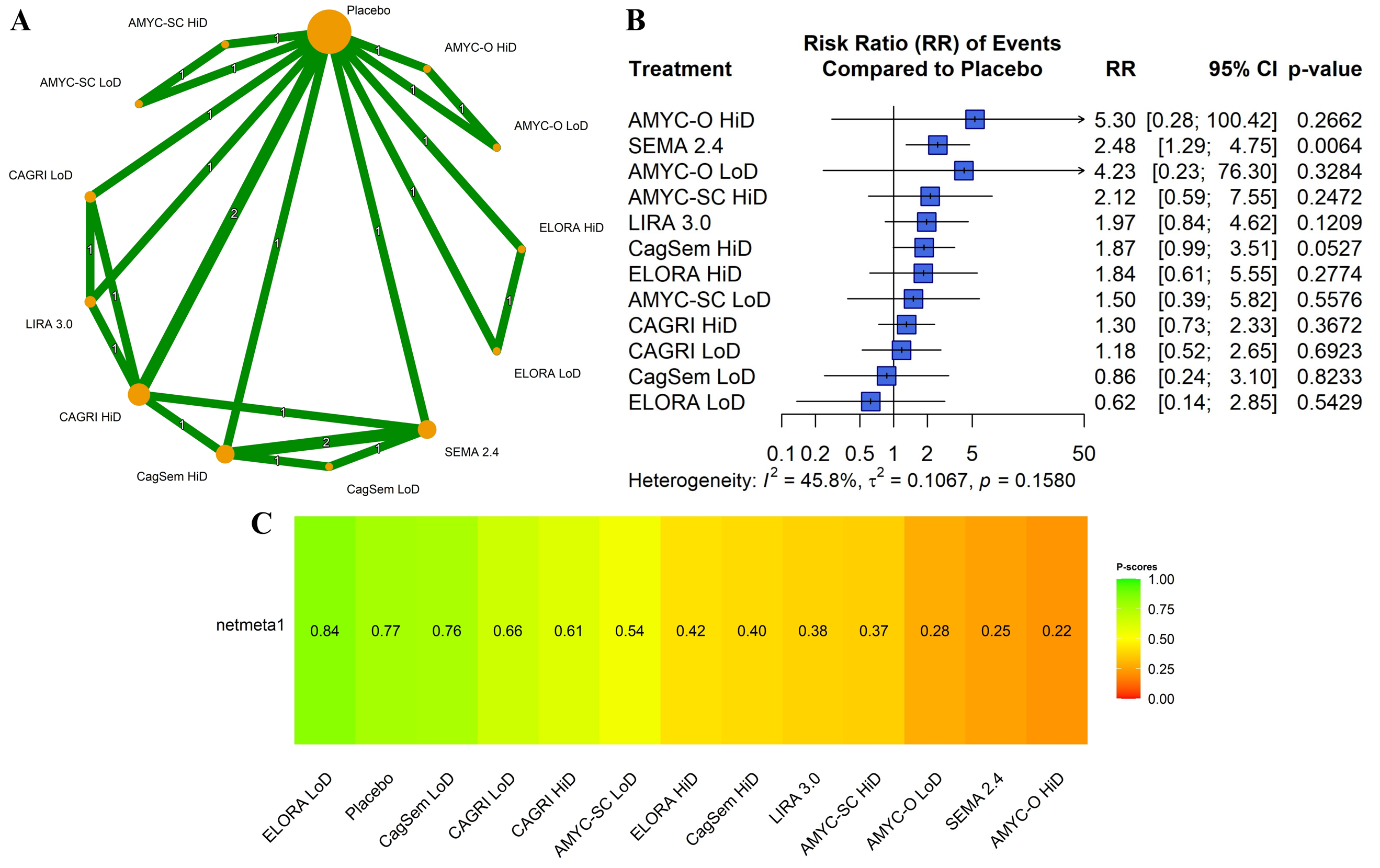


**Figure S32.** League table showing head-to-head comparisons among the interventions for the proportions of study subjects who experienced diarrhea

**
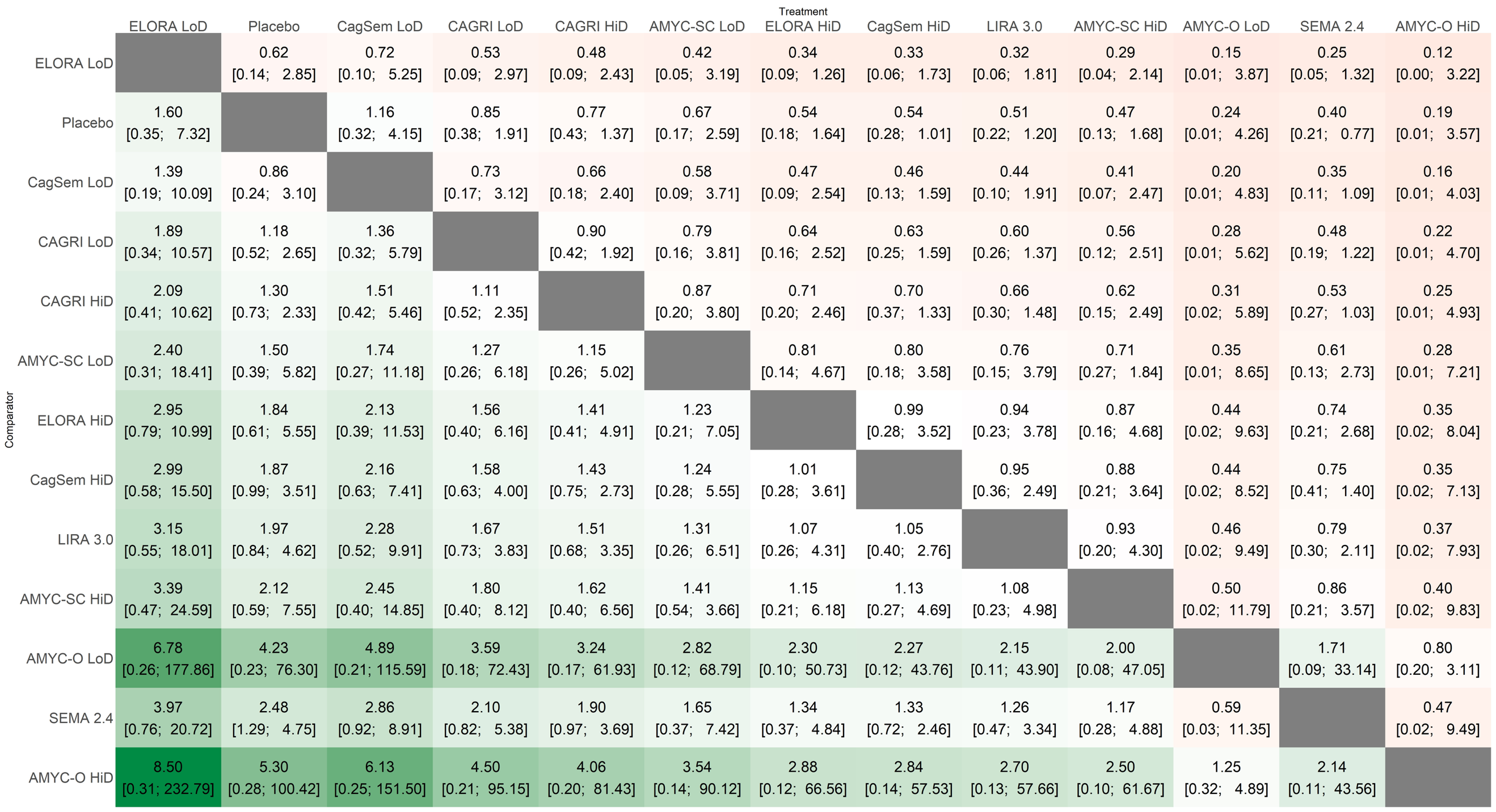
**

**Figure S33.** Netsplit estimates for separate direct and indirect evidence for the proportions of study subjects who experienced diarrhea

**
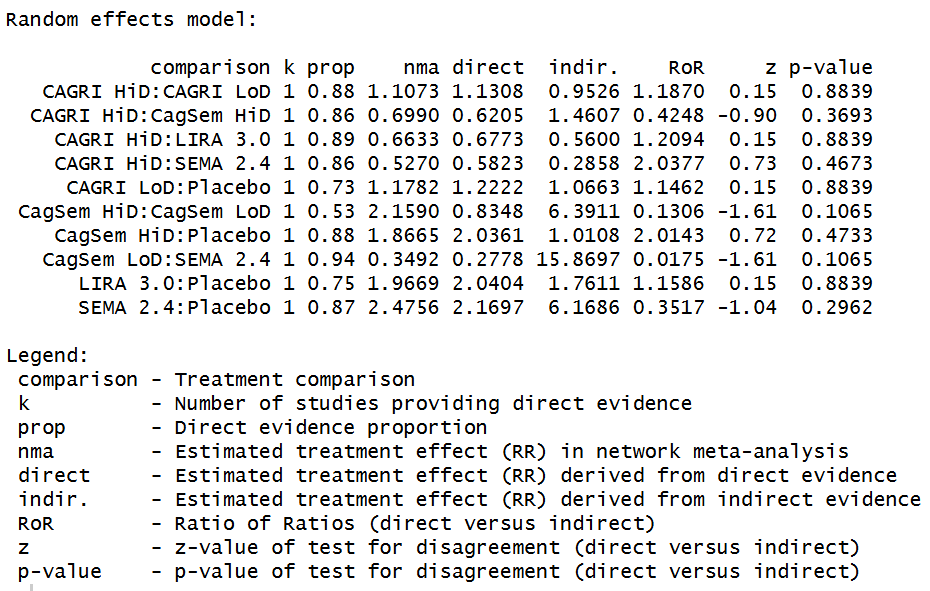
**

**Figure S34.** Network diagram (A), network meta-analysis forest plot (B), P score (C) for the proportions of study subjects who experienced constipation, comparing various amylin-based therapies to placebo

[Five studies (12 treatments, 21 pairwise comparisons), No heterogeneity and inconsistency (tau^2^ = 0.0015; tau = 0.0389; I^2^ = 1.7%), non-significant Q]


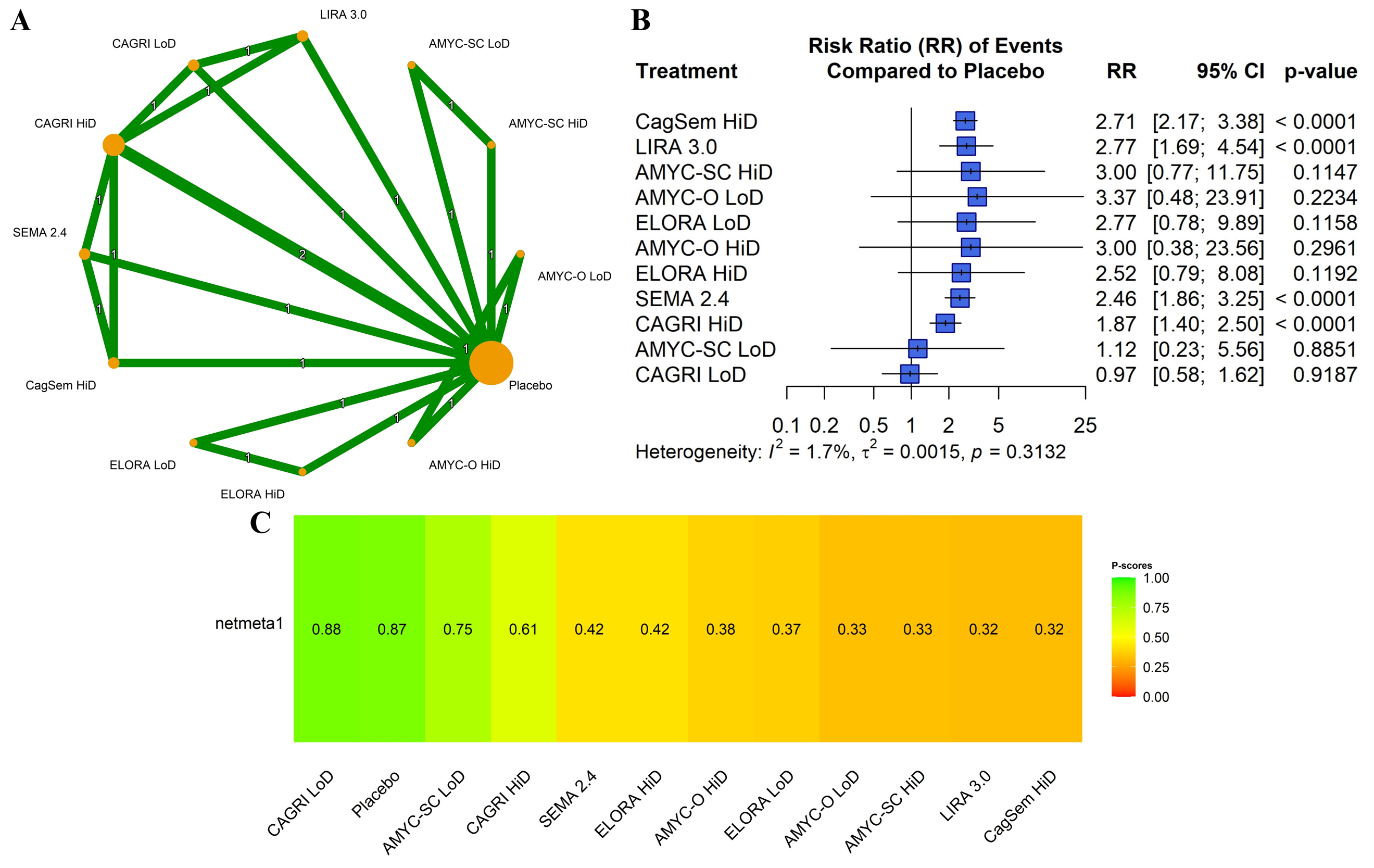


**Figure S35.** League table showing head-to-head comparisons among the interventions for the proportions of study subjects who experienced constipation

**
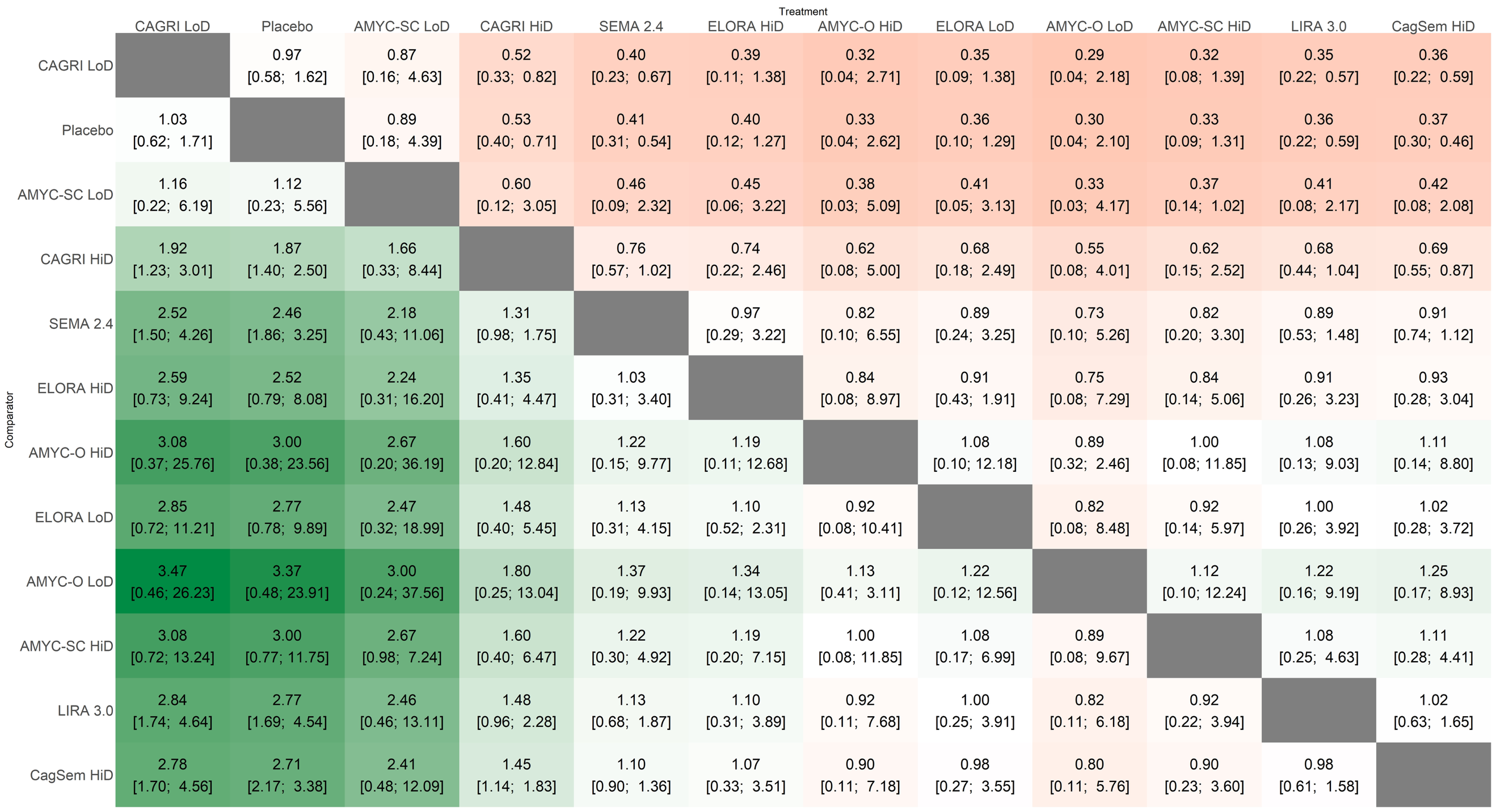
**

**Figure S36.** Netsplit estimates for separate direct and indirect evidence for the proportions of study subjects who experienced constipation

**
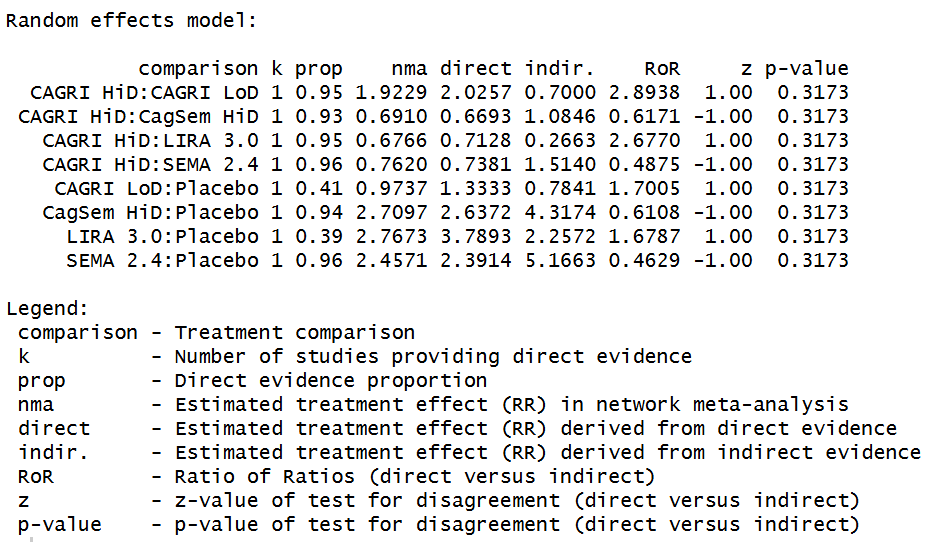
**

**Figure S37.** Network diagram (A), network meta-analysis forest plot (B), P score (C) for the proportions of study subjects with adverse events leading to discontinuation, comparing various amylin-based therapies to placebo

[Six studies (13 treatments, 24 pairwise comparisons), mild heterogeneity and inconsistency (tau^2^ = 0; tau = 0; I^2^ = 0%), non-significant Q]


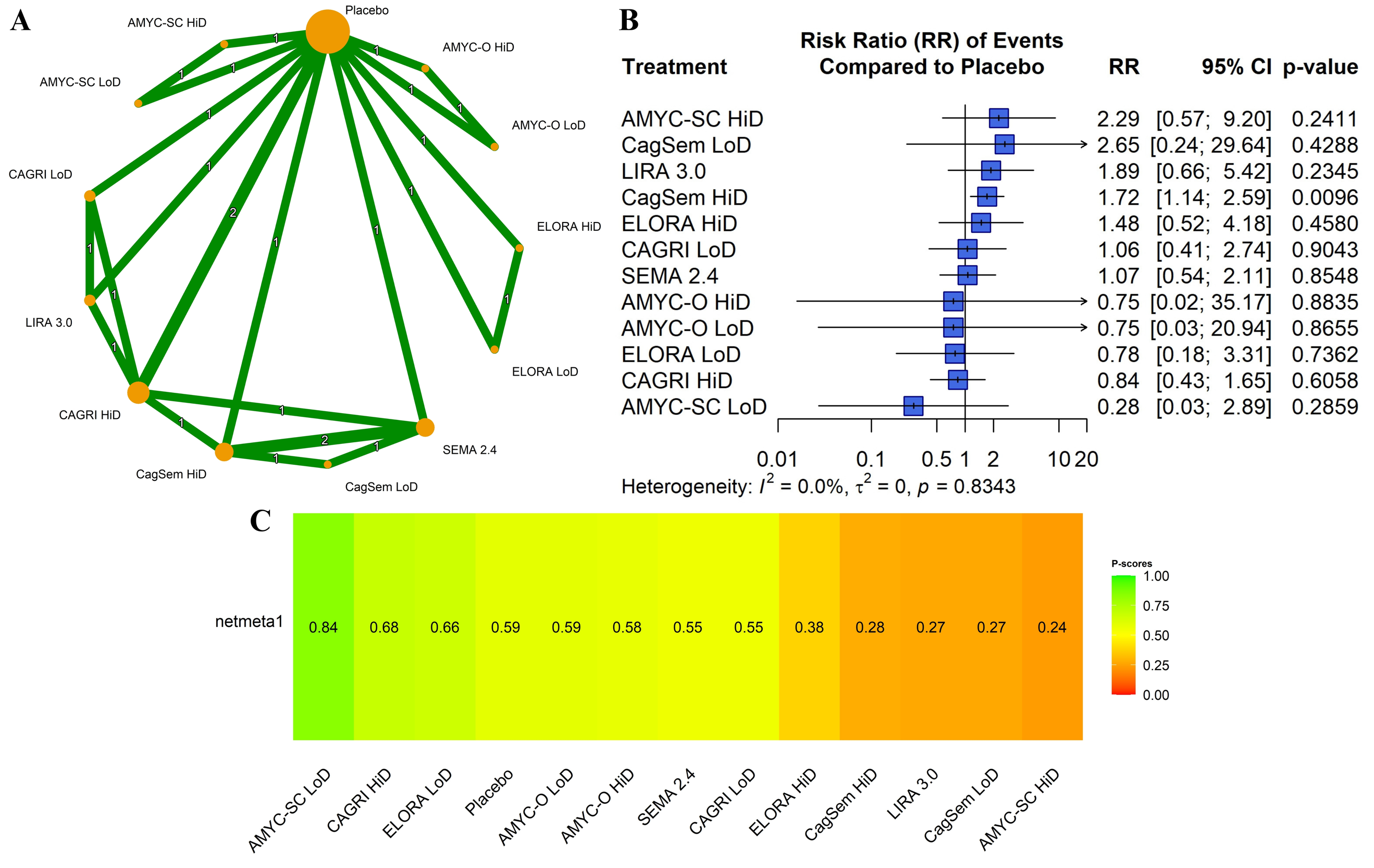


**Figure S38.** League table showing head-to-head comparisons among the interventions for the proportions of study subjects with adverse events leading to discontinuation

**
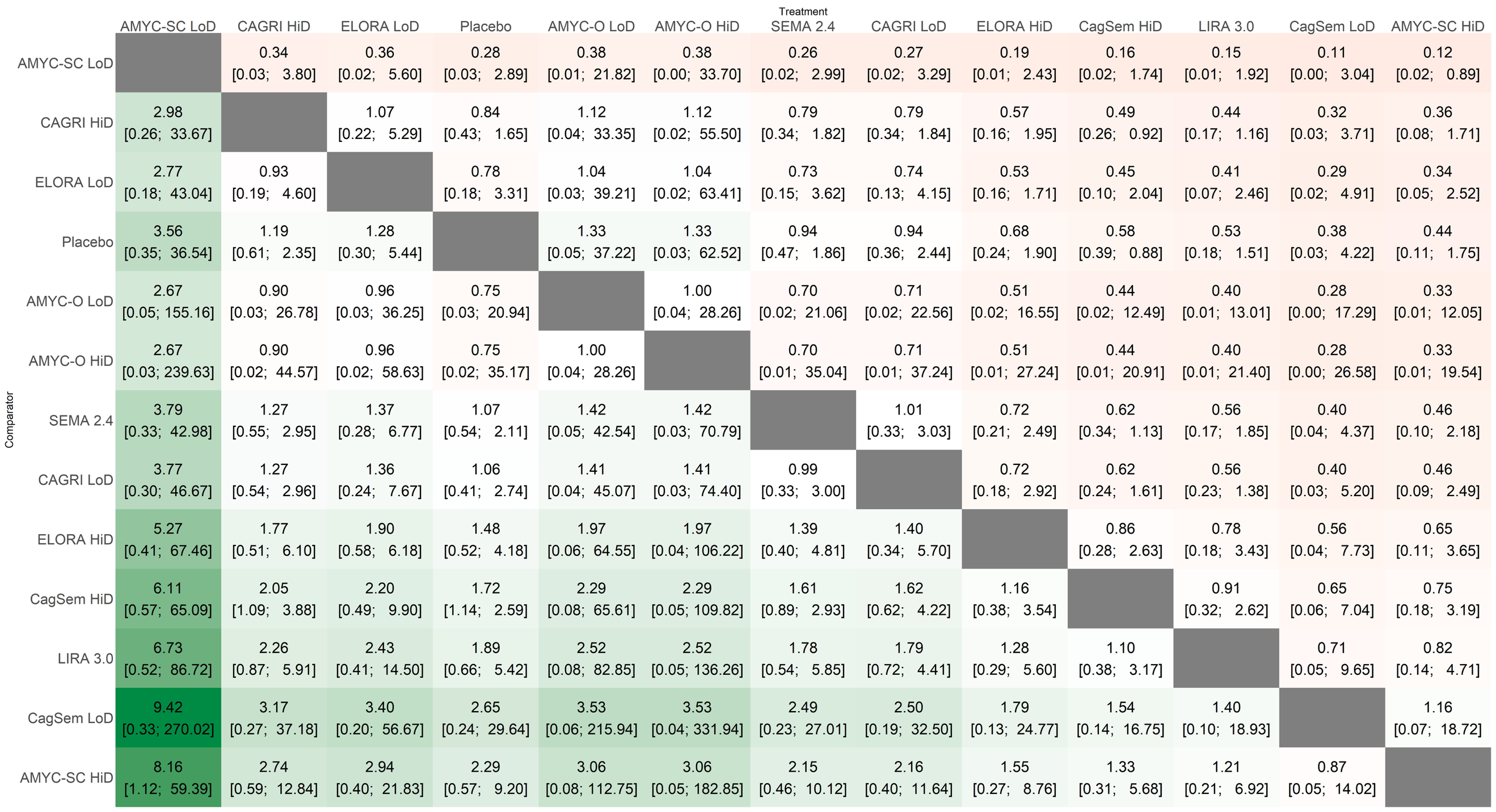
**

**Figure S39.** Netsplit estimates for separate direct and indirect evidence for the proportions of study subjects with adverse events leading to discontinuation

**
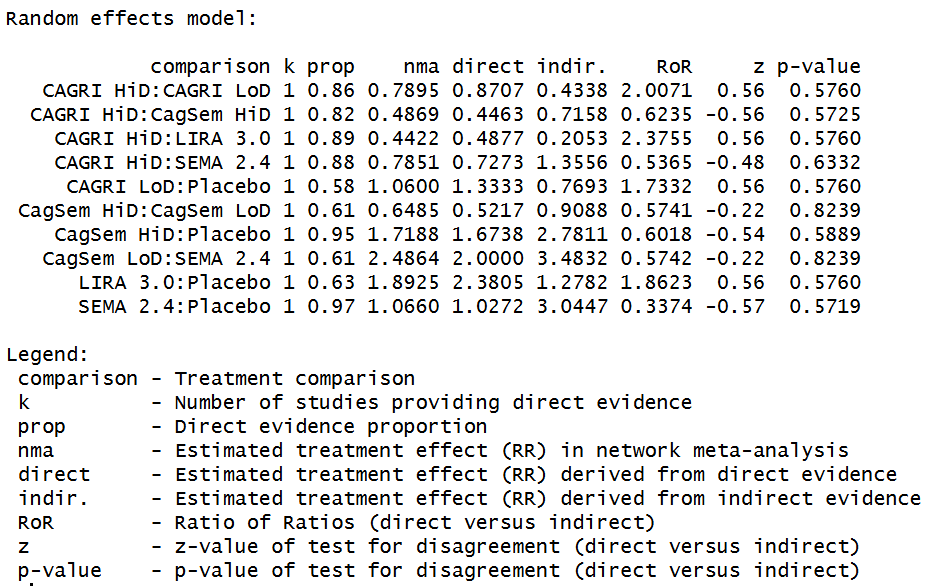
**
