# Supplementary material for: Novel Amylin‐Based Therapies for Weight Management in Adults With Overweight or Obesity Without Diabetes: A Network Meta‐Analysis
Source: Endocrinol Diabetes Metab. 2026 May 22;9(3):e70247. doi: 10.1002/edm2.70247 (PMC13240113; doi:10.1002/edm2.70247)
Supplement: Supplementary file 1 — Table S1: Search strings. Table S2: Results from individual studies' efficacy outcomes. Table S3: Results from individual studies' safety outcomes. Figure S1: (A) Risk of bias summary: Review authors' judgements about each risk of bias item for each included study; (B) risk of bias graph: Review authors' judgements about each risk of bias item presented as percentages across all included studies. Figure S2: Comparison‐adjusted funnel plots for the interventions for the percent change in body weight. Figure S3: Netsplit estimates for separate direct and indirect evidence for the percent change in body weight. Figure S4: Network diagram (A), network meta‐analysis forest plot (B), P score (C) for the absolute change in body weight, comparing various amylin‐based therapies to placebo. Figure S5: League table showing head‐to‐head comparisons among the interventions for absolute changes in body weight (kg). Figure S6: Netsplit estimates for separate direct and indirect evidence for absolute changes in body weight (kg). Figure S7: Network diagram (A), network meta‐analysis forest plot (B), P score (C) for the change in body mass index, comparing various amylin‐based therapies to placebo. Figure S8: League table showing head‐to‐head comparisons among the interventions for changes in body mass index. Figure S9: Netsplit estimates for separate direct and indirect evidence for changes in body mass index. Figure S10: Network diagram (A), network meta‐analysis forest plot (B), P score (C) for the change in waist circumference, comparing various amylin‐based therapies to placebo. Figure S11: League table showing head‐to‐head comparisons among the interventions for changes in waist circumference. Figure S12: Netsplit estimates for separate direct and indirect evidence for changes in waist circumference. Figure S13: Network diagram (A), network meta‐analysis forest plot (B), P score (C) for the proportions of study subjects who lost ≥ 5% of their baseline body weight, comparing var [file edm270247-sup-0001-FigureS40@Figure_S40._CINeMA.pdf › edm270247-sup-0001-FigureS40@Figure_S40._CINeMA.pdf]

Body Weight Percentage Change

Download Report Reset

Comparison	Number of Studies	Within-study bias	Reporting bias	Indirectness	Imprecision	Heterogeneity	Incoherence	Confidence rating	Reason(s) for downgrading
				Mixed evidence					
AMYC-O HiD vs AMYC-O LoD	1	Major concerns	Low risk	No concerns	Major concerns	No concerns	No concerns	Very low	| Within-study bias | Imprecision |
AMYC-O HiD vs Placebo	1	Major concerns	Low risk	No concerns	No concerns	No concerns	No concerns	Very low	| Within-study bias |
AMYC-O LoD vs Placebo	1	Major concerns	Low risk	No concerns	No concerns	No concerns	No concerns	Very low	| Within-study bias |
AMYC-SC HiD vs AMYC-SC LoD	1	Some concerns	Low risk	No concerns	No concerns	No concerns	No concerns	Low	| Within-study bias |
AMYC-SC HiD vs Placebo	1	Some concerns	Low risk	No concerns	No concerns	No concerns	No concerns	Low	| Within-study bias |
AMYC-SC LoD vs Placebo	1	Some concerns	Low risk	No concerns	No concerns	No concerns	No concerns	Low	| Within-study bias |
CAGRI HiD vs CAGRI LoD	1	Some concerns	Low risk	No concerns	No concerns	No concerns	No concerns	Low	| Within-study bias |
CAGRI HiD vs CagSem HiD	1	No concerns	Low risk	No concerns	No concerns	No concerns	No concerns	Moderate	|
CAGRI HiD vs LIRA 3.0	1	Some concerns	Low risk	No concerns	No concerns	Major concerns	No concerns	Very low	| Within-study bias | Heterogeneity |
CAGRI HiD vs Placebo	2	Some concerns	Low risk	No concerns	No concerns	No concerns	No concerns	Low	| Within-study bias |
CAGRI HiD vs SEMA 2.4	1	No concerns	Low risk	No concerns	No concerns	No concerns	No concerns	Moderate	|
CAGRI LoD vs LIRA 3.0	1	Some concerns	Low risk	No concerns	No concerns	Major concerns	No concerns	Very low	| Within-study bias | Heterogeneity |
CAGRI LoD vs Placebo	1	Some concerns	Low risk	No concerns	No concerns	No concerns	No concerns	Low	| Within-study bias |
CagSem HiD vs CagSem LoD	1	Some concerns	Low risk	No concerns	No concerns	Major concerns	No concerns	Very low	| Within-study bias | Heterogeneity |
CagSem HiD vs Placebo	1	No concerns	Low risk	No concerns	No concerns	No concerns	No concerns	Moderate	|
CagSem HiD vs SEMA 2.4	2	No concerns	Low risk	No concerns	No concerns	No concerns	No concerns	Moderate	|
CagSem LoD vs SEMA 2.4	1	Some concerns	Low risk	No concerns	Major concerns	No concerns	No concerns	Very low	| Within-study bias | Imprecision |
ELORA HiD vs ELORA LoD	1	Some concerns	Low risk	No concerns	No concerns	No concerns	No concerns	Low	| Within-study bias |
ELORA HiD vs Placebo	1	Some concerns	Low risk	No concerns	No concerns	No concerns	No concerns	Low	| Within-study bias |
ELORA LoD vs Placebo	1	Some concerns	Low risk	No concerns	No concerns	No concerns	No concerns	Low	| Within-study bias |
LIRA 3.0 vs Placebo	1	Some concerns	Low risk	No concerns	No concerns	No concerns	No concerns	Low	| Within-study bias |
Placebo vs SEMA 2.4	1	No concerns	Low risk	No concerns	No concerns	No concerns	No concerns	Moderate	|
				Indirect evidence					
AMYC-O HiD vs AMYC-SC HiD	--	Major concerns	Low risk	No concerns	No concerns	No concerns	No concerns	Very low	| Within-study bias |
AMYC-O HiD vs AMYC-SC LoD	--	Some concerns	Low risk	No concerns	Major concerns	No concerns	No concerns	Very low	| Within-study bias | Imprecision |
AMYC-O HiD vs CAGRI HiD	--	Some concerns	Low risk	No concerns	No concerns	Major concerns	No concerns	Very low	| Within-study bias | Heterogeneity |
AMYC-O HiD vs CAGRI LoD	--	Some concerns	Low risk	No concerns	No concerns	No concerns	No concerns	Low	| Within-study bias |
AMYC-O HiD vs CagSem HiD	--	Some concerns	Low risk	No concerns	No concerns	Major concerns	No concerns	Very low	| Within-study bias | Heterogeneity |
AMYC-O HiD vs CagSem LoD	--	Some concerns	Low risk	No concerns	Major concerns	No concerns	No concerns	Very low	| Within-study bias | Imprecision |
AMYC-O HiD vs ELORA HiD	--	Major concerns	Low risk	No concerns	No concerns	Major concerns	No concerns	Very low	| Within-study bias | Heterogeneity |
AMYC-O HiD vs ELORA LoD	--	Some concerns	Low risk	No concerns	Major concerns	No concerns	No concerns	Very low	| Within-study bias | Imprecision |
AMYC-O HiD vs LIRA 3.0	--	Some concerns	Low risk	No concerns	No concerns	Major concerns	No concerns	Very low	| Within-study bias | Heterogeneity |
AMYC-O HiD vs SEMA 2.4	--	Some concerns	Low risk	No concerns	Major concerns	No concerns	No concerns	Very low	| Within-study bias | Imprecision |
AMYC-O LoD vs AMYC-SC HiD	--	Some concerns	Low risk	No concerns	No concerns	No concerns	No concerns	Low	| Within-study bias |
AMYC-O LoD vs AMYC-SC LoD	--	Some concerns	Low risk	No concerns	No concerns	Major concerns	No concerns	Very low	| Within-study bias | Heterogeneity |
AMYC-O LoD vs CAGRI HiD	--	Some concerns	Low risk	No concerns	Major concerns	No concerns	No concerns	Very low	| Within-study bias | Imprecision |
AMYC-O LoD vs CAGRI LoD	--	Some concerns	Low risk	No concerns	No concerns	Major concerns	No concerns	Very low	| Within-study bias | Heterogeneity |
AMYC-O LoD vs CagSem HiD	--	Some concerns	Low risk	No concerns	No concerns	No concerns	No concerns	Low	| Within-study bias |
AMYC-O LoD vs CagSem LoD	--	Some concerns	Low risk	No concerns	Major concerns	No concerns	No concerns	Very low	| Within-study bias | Imprecision |
AMYC-O LoD vs ELORA HiD	--	Some concerns	Low risk	No concerns	No concerns	No concerns	No concerns	Low	| Within-study bias |
AMYC-O LoD vs ELORA LoD	--	Some concerns	Low risk	No concerns	Major concerns	No concerns	No concerns	Very low	| Within-study bias | Imprecision |
AMYC-O LoD vs LIRA 3.0	--	Some concerns	Low risk	No concerns	No concerns	Major concerns	No concerns	Very low	| Within-study bias | Heterogeneity |
AMYC-O LoD vs SEMA 2.4	--	Some concerns	Low risk	No concerns	Major concerns	No concerns	No concerns	Very low	| Within-study bias | Imprecision |
AMYC-SC HiD vs CAGRI HiD	--	Some concerns	Low risk	No concerns	No concerns	No concerns	No concerns	Low	| Within-study bias |
AMYC-SC HiD vs CAGRI LoD	--	Some concerns	Low risk	No concerns	No concerns	No concerns	No concerns	Low	| Within-study bias |
AMYC-SC HiD vs CagSem HiD	--	Some concerns	Low risk	No concerns	No concerns	Major concerns	No concerns	Very low	| Within-study bias | Heterogeneity |
AMYC-SC HiD vs CagSem LoD	--	Some concerns	Low risk	No concerns	No concerns	No concerns	No concerns	Low	| Within-study bias |
AMYC-SC HiD vs ELORA HiD	--	Some concerns	Low risk	No concerns	No concerns	Major concerns	No concerns	Very low	| Within-study bias | Heterogeneity |
AMYC-SC HiD vs ELORA LoD	--	Some concerns	Low risk	No concerns	No concerns	No concerns	No concerns	Low	| Within-study bias |
AMYC-SC HiD vs LIRA 3.0	--	Some concerns	Low risk	No concerns	No concerns	No concerns	No concerns	Low	| Within-study bias |
AMYC-SC HiD vs SEMA 2.4	--	Some concerns	Low risk	No concerns	No concerns	No concerns	No concerns	Low	| Within-study bias |
AMYC-SC LoD vs CAGRI HiD	--	Some concerns	Low risk	No concerns	No concerns	Major concerns	No concerns	Very low	| Within-study bias | Heterogeneity |
AMYC-SC LoD vs CAGRI LoD	--	Some concerns	Low risk	No concerns	No concerns	No concerns	No concerns	Low	| Within-study bias |
AMYC-SC LoD vs CagSem HiD	--	Some concerns	Low risk	No concerns	Major concerns	No concerns	No concerns	Very low	| Within-study bias | Imprecision |
AMYC-SC LoD vs CagSem LoD	--	Some concerns	Low risk	No concerns	Major concerns	No concerns	No concerns	Very low	| Within-study bias | Imprecision |
AMYC-SC LoD vs ELORA HiD	--	Some concerns	Low risk	No concerns	Major concerns	No concerns	No concerns	Very low	| Within-study bias | Imprecision |
AMYC-SC LoD vs ELORA LoD	--	Some concerns	Low risk	No concerns	Major concerns	No concerns	No concerns	Very low	| Within-study bias | Imprecision |
AMYC-SC LoD vs LIRA 3.0	--	Some concerns	Low risk	No concerns	No concerns	Major concerns	No concerns	Very low	| Within-study bias | Heterogeneity |
AMYC-SC LoD vs SEMA 2.4	--	Some concerns	Low risk	No concerns	Major concerns	No concerns	No concerns	Very low	| Within-study bias | Imprecision |
CAGRI HiD vs CagSem LoD	--	Some concerns	Low risk	No concerns	No concerns	Major concerns	No concerns	Very low	| Within-study bias | Heterogeneity |
CAGRI HiD vs ELORA HiD	--	Some concerns	Low risk	No concerns	No concerns	No concerns	No concerns	Low	| Within-study bias |
CAGRI HiD vs ELORA LoD	--	Some concerns	Low risk	No concerns	Major concerns	No concerns	No concerns	Very low	| Within-study bias | Imprecision |
CAGRI LoD vs CagSem HiD	--	Some concerns	Low risk	No concerns	No concerns	No concerns	No concerns	Low	| Within-study bias |
CAGRI LoD vs CagSem LoD	--	Some concerns	Low risk	No concerns	No concerns	No concerns	No concerns	Low	| Within-study bias |
CAGRI LoD vs ELORA HiD	--	Some concerns	Low risk	No concerns	No concerns	No concerns	No concerns	Low	| Within-study bias |
CAGRI LoD vs ELORA LoD	--	Some concerns	Low risk	No concerns	No concerns	Major concerns	No concerns	Very low	| Within-study bias | Heterogeneity |
CAGRI LoD vs SEMA 2.4	--	No concerns	Low risk	No concerns	No concerns	No concerns	No concerns	Moderate	|
CagSem HiD vs ELORA HiD	--	Some concerns	Low risk	No concerns	Major concerns	No concerns	No concerns	Very low	| Within-study bias | Imprecision |
CagSem HiD vs ELORA LoD	--	Some concerns	Low risk	No concerns	No concerns	Major concerns	No concerns	Very low	| Within-study bias | Heterogeneity |
CagSem HiD vs LIRA 3.0	--	Some concerns	Low risk	No concerns	No concerns	No concerns	No concerns	Low	| Within-study bias |
CagSem LoD vs ELORA HiD	--	Some concerns	Low risk	No concerns	No concerns	Major concerns	No concerns	Very low	| Within-study bias | Heterogeneity |
CagSem LoD vs ELORA LoD	--	Some concerns	Low risk	No concerns	Major concerns	No concerns	No concerns	Very low	| Within-study bias | Imprecision |
CagSem LoD vs LIRA 3.0	--	Some concerns	Low risk	No concerns	No concerns	Major concerns	No concerns	Very low	| Within-study bias | Heterogeneity |
CagSem LoD vs Placebo	--	Some concerns	Low risk	No concerns	No concerns	No concerns	No concerns	Low	| Within-study bias |
ELORA HiD vs LIRA 3.0	--	Some concerns	Low risk	No concerns	No concerns	No concerns	No concerns	Low	| Within-study bias |
ELORA HiD vs SEMA 2.4	--	No concerns	Low risk	No concerns	No concerns	No concerns	No concerns	Moderate	|
ELORA LoD vs LIRA 3.0	--	Some concerns	Low risk	No concerns	No concerns	Major concerns	No concerns	Very low	| Within-study bias | Heterogeneity |
ELORA LoD vs SEMA 2.4	--	Some concerns	Low risk	No concerns	Major concerns	No concerns	No concerns	Very low	| Within-study bias | Imprecision |
LIRA 3.0 vs SEMA 2.4	--	Some concerns	Low risk	No concerns	No concerns	No concerns	No concerns	Low	| Within-study bias |

CINeMA is distributed, in the hope that it will be useful but without any warranty, under the GPL license. By using CINeMA you accept the following DISCLAIMER
